# Study protocol: A randomized controlled trial of chemoradiotherapy versus chemotherapy as neoadjuvant therapy for resectable pancreatic cancer (CSGO-HBP-027)

**DOI:** 10.1371/journal.pone.0345459

**Published:** 2026-03-26

**Authors:** Yoshito Tomimaru, Hirofumi Akita, Keisuke Tamari, Sakae Maeda, Hiroshi Wada, Yoshifumi Iwagami, Chikato Koga, Naoki Hama, Akira Tomokuni, Shogo Kobayashi, Tadafumi Asaoka, Masanori Tsujie, Masahiro Tanemura, Masahiro Murakami, Yoshiteru Katsura, Junzo Shimizu, Masashi Yamashita, Shigeru Sakamoto, Satomi Okamura, Tomomi Yamada, Yuichiro Doki, Hidetoshi Eguchi

**Affiliations:** 1 Department of Gastroenterological Surgery, Graduate School of Medicine, The University of Osaka, Suita, Japan; 2 Department of Radiation Oncology, Graduate School of Medicine, The University of Osaka, Suita, Japan; 3 Department of Gastroenterological Surgery, Ikeda City Hospital, Ikeda, Japan; 4 Department of Surgery, Japan Community Health Care Organization Osaka Hospital, Osaka, Japan; 5 Department of Gastroenterological Surgery, Kansai Rosai Hospital, Amagasaki, Japan; 6 Department of Gastroenterological Surgery, Kindai University Nara Hospital, Ikoma, Japan; 7 Department of Gastroenterological Surgery, National Hospital Organization Osaka National Hospital, Osaka, Japan; 8 Department of Gastroenterological Surgery, Osaka General Medical Center, Osaka, Japan; 9 Department of Gastroenterological Surgery, Osaka International Cancer Institute, Osaka, Japan; 10 Department of Gastroenterological Surgery, Osaka Police Hospital, Osaka, Japan; 11 Department of Gastroenterological Surgery, Osaka Rosai Hospital, Osaka, Japan; 12 Department of Gastroenterological Surgery, Rinku General Medical Center, Izumisano, Japan; 13 Department of Hepato-Biliary-Pancreatic Surgery, Sakai City Medical Center, Sakai, Japan; 14 Department of Surgery, Suita Municipal Hospital, Suita, Japan; 15 Department of Gastroenterological Surgery, Toyonaka Municipal Hospital, Toyonaka, Japan; 16 Department of Gastroenterological Surgery, Higashiosaka City Medical Center, Higashiosaka, Japan; 17 Department of Medical Innovation, The University of Osaka Hospital, Suita, Japan; Shandong Cancer Hospital and Institute Shandong First Medical University and Shandong Academy of Medical Sciences: Shandong Cancer Hospital and Institute, CHINA

## Abstract

Despite advances in multimodal treatment, the long-term survival of patients with resectable pancreatic ductal adenocarcinoma (PDAC) remains poor. In Japan, neoadjuvant chemotherapy with gemcitabine and S-1 (GS) has demonstrated a survival benefit compared with upfront surgery. The addition of radiotherapy to chemotherapy may further improve outcomes by enhancing local tumor control and increasing R0 resection rates; however, no randomized trial has directly compared GS alone with GS plus radiotherapy (GS-RT) in patients with resectable PDAC. The CSGO-HBP-027 trial is a multicenter, randomized phase II/III study designed to evaluate whether neoadjuvant GS-RT improves survival compared with GS alone. This trial will enroll 200 patients with resectable PDAC, who will be randomized in a 1:1 ratio to receive either two cycles of GS or two cycles of GS with concurrent radiotherapy (50.4 Gy in 28 fractions), followed by surgery scheduled 3–8 weeks after completion of neoadjuvant therapy. The primary endpoint is overall survival, and secondary endpoints include resection rate, R0 resection rate, histological tumor response, progression-free survival, and safety. Overall survival will be compared between the two treatment arms using the stratified log-rank test, with adjusted hazard ratios estimated using the Cox proportional hazards model. This trial was registered with the Japan Registry of Clinical Trials (jRCTs051250150). The CSGO-HBP-027 trial will provide evidence on whether neoadjuvant GS-RT improves survival in patients with resectable PDAC compared with GS alone. The results of this study are expected to clarify the optimal neoadjuvant treatment strategy for resectable PDAC and to provide high-quality evidence regarding the clinical value of adding radiotherapy to GS-based neoadjuvant therapy.

## Introduction

Pancreatic ductal adenocarcinoma (PDAC) is one of the most aggressive and lethal malignancies, with a 5-year survival rate <20% globally [1–3]. The majority of patients with PDAC are diagnosed at an advanced stage due to the lack of early symptoms and effective screening methods. Only approximately 20% of patients diagnosed with PDAC are considered eligible for curative resection at the time of diagnosis, and recurrence is common, even after successful surgery, and long-term survival remains unsatisfactory [4]. Therefore, surgery alone is no longer considered sufficient, and multidisciplinary treatment strategies have been increasingly explored. In Japan, the Prep-02/JSAP05 trial showed that the median overall survival (OS) in patients with resectable and borderline resectable PDAC is significantly longer with neoadjuvant chemotherapy using a combination of gemcitabine and S-1 (GS) than with upfront surgery (37.0 vs. 26.6 months; hazard ratio = 0.73, p = 0.018) [5]. Based on this evidence, the Japanese clinical practice guidelines now recommend GS as a standard neoadjuvant regimen for resectable PDAC [6].

There has also been growing interest in incorporating radiotherapy into the neoadjuvant setting based on the concept that radiotherapy may enhance local tumor control, increase R0 resection rates, and reduce micrometastases in patients with PDAC [7,8]. The Dutch randomized controlled trial PREOPANC-1 demonstrated that neoadjuvant chemoradiotherapy with gemcitabine significantly improves OS, the R0 resection rate, and long-term survival compared with upfront surgery in patients with resectable and borderline resectable PDAC [9,10]. This trial provided the first high-level evidence supporting neoadjuvant chemoradiotherapy in patients with resectable and borderline resectable PDAC. Our group also conducted a prospective study that suggested that the addition of radiotherapy to GS (GS-RT) may further improve oncological outcomes [11]. However, few randomized controlled trials have directly compared neoadjuvant chemoradiotherapy to chemotherapy alone in patients with resectable PDAC. Therefore, to address this gap, we initiated a multicenter, randomized phase II/III trial (CSGO-HBP-027) to compare GS-RT and GS alone. The primary aim is to determine whether GS-RT improves OS and other clinically relevant outcomes, thereby establishing a new standard of care for resectable PDAC. The potential survival benefit of GS-RT may be mediated through improved local tumor control, a higher rate of margin-negative resection, and a reduction in local recurrence.

## Methods

### Study design

This is a multicenter, open-label, randomized controlled trial (CSGO-HBP-027) designed to compare the efficacy and safety of neoadjuvant chemoradiotherapy (GS-RT arm) with neoadjuvant chemotherapy alone (GS arm) in patients with resectable PDAC. The trial is being coordinated by the Clinical Study Group of Osaka University-Hepato-Biliary-Pancreatic group (CSGO-HBP) and includes 15 participating institutions (Table 1). Patients who meet all the inclusion criteria and none of the exclusion criteria are eligible for enrollment (Table 2). Resectable PDAC is defined based on the National Comprehensive Cancer Network (NCCN) guidelines [12]. The investigators will enter the required information for each consenting patient into the web-based registration system Research Electronic Data Capture (REDCap) [13]. The REDCap system will automatically assess patient eligibility and determine treatment allocation for patients who meet all eligibility criteria. Stratified permuted block randomization will be applied using the baseline serum CA19−9 level (<370 vs. ≥ 370 U/mL) as a stratification factor prior to treatment initiation. The study protocol was approved by the ethics committee of The University of Osaka Clinical Research Review Board (Certification No. CRB5180007) and was registered with Japan Registry of Clinical Trials (jRCTs051250150).

**Table 1:** Participating Institutions.

Name of institution
Ikeda City Hospital Japan Community Health Care Organization Osaka Hospital Kansai Rosai Hospital Kindai University Nara Hospital National Hospital Organization Osaka National Hospital Osaka General Medical Center Osaka International Cancer Institute Osaka Police Hospital Osaka Rosai Hospital Rinku General Medical Center Sakai City Medical Center Suita Municipal Hospital The University of Osaka Hospital Toyonaka Municipal Hospital Higashiosaka City Medical Center

**Table 2:** Eligibility criteria for the study.

**Inclusion criteria** 1) Histologically and/or cytologically confirmed PDAC. 2) Age ≥ 18 years at the time of informed consent. 3) Eastern Cooperative Oncology Group performance status (ECOG PS) of 0–1. 4) Radiologically diagnosed resectable PDAC according to the NCCN Clinical Practice Guidelines in Oncology, Pancreatic Adenocarcinoma, version 2.2025: Resectability must be assessed by multidetector CT with a slice thickness ≤ 5 mm. Additional examinations, such as MRI, ultrasonography, PET/CT, and staging laparoscopy, may be performed as needed. Resectable PDAC is defined by the following imaging criteria: i) No tumor contact with the celiac axis, superior mesenteric artery, or common hepatic artery; ii) No tumor contact with the superior mesenteric vein or portal vein, or ≤180° contact without vein contour irregularity; iii) No tumor contact with the aorta or inferior vena cava. 5) No prior treatment for PDAC; protocol treatment must be the initial therapy. 6) Expected survival ≥ 6 months from the time of informed consent. 7) No evidence of direct tumor invasion into the gastrointestinal tract. 8) Adequate function of major organs (bone marrow, liver, kidney, lung), defined as: White blood cell count ≥ 3,500/mm³; Neutrophil count ≥ 2,000/mm³; Platelet count ≥ 100,000/mm³; Hemoglobin ≥ 9.0 g/dL; Serum total bilirubin ≤ 2.0 mg/dL (≤ 3.0 mg/dL in patients after biliary drainage for obstructive jaundice); AST and ALT ≤ 150 U/L; Serum creatinine ≤ 1.2 mg/dL and creatinine clearance ≥ 60 mL/min (estimated by the Cockcroft–Gault equation if necessary). 9) Written informed consent obtained from the patient prior to study enrollment.
**Exclusion criteria** 1) Unresectable PDAC, defined as any of the following: Presence of distant organ metastasis; Peritoneal dissemination or positive peritoneal cytology; Para-aortic lymph node metastasis; Tumor contact >180° with the portal vein or superior mesenteric vein; Tumor contact with the aorta, celiac axis, superior mesenteric artery, or common hepatic artery. 2) Patients with pulmonary fibrosis, interstitial pneumonia, a history of either condition, or CT findings suggestive of these conditions, or with severe pulmonary emphysema, residual inflammatory changes, or markedly impaired pulmonary function in pre-treatment testing. 3) Patients with active infection, excluding cholangitis and viral hepatitis. 4) Patients with severe comorbidities, such as heart failure, renal failure, hepatic failure, hemorrhagic peptic ulcer, paralytic ileus, bowel obstruction, or uncontrolled diabetes mellitus. 5) Patients with moderate-to-severe ascites or pleural effusion. 6) Patients with active double cancer (synchronous malignancy or metachronous malignancy within 2 years). However, carcinoma in situ or intramucosal carcinoma lesions considered cured by local therapy are not regarded as active double cancers. 7) Patients receiving flucytosine, phenytoin, or warfarin potassium. 8) Pregnant or breastfeeding women, women who are pregnant or may become pregnant, and men whose partners wish to become pregnant. 9) Patients with a history of severe drug hypersensitivity. 10) Patients currently participating in another interventional clinical study. 11) Any condition deemed unsuitable for study participation by the investigators.

Abbreviations: ALT, alanine aminotransferase; AST, aspartate aminotransferase; CT, computed tomography; MRI, magnetic resonance imaging; NCCN, National Comprehensive Cancer Network; PDAC, pancreatic ductal adenocarcinoma; PET/CT, positron emission tomography–computed tomography.

### Treatment protocols

**Fig 1:**
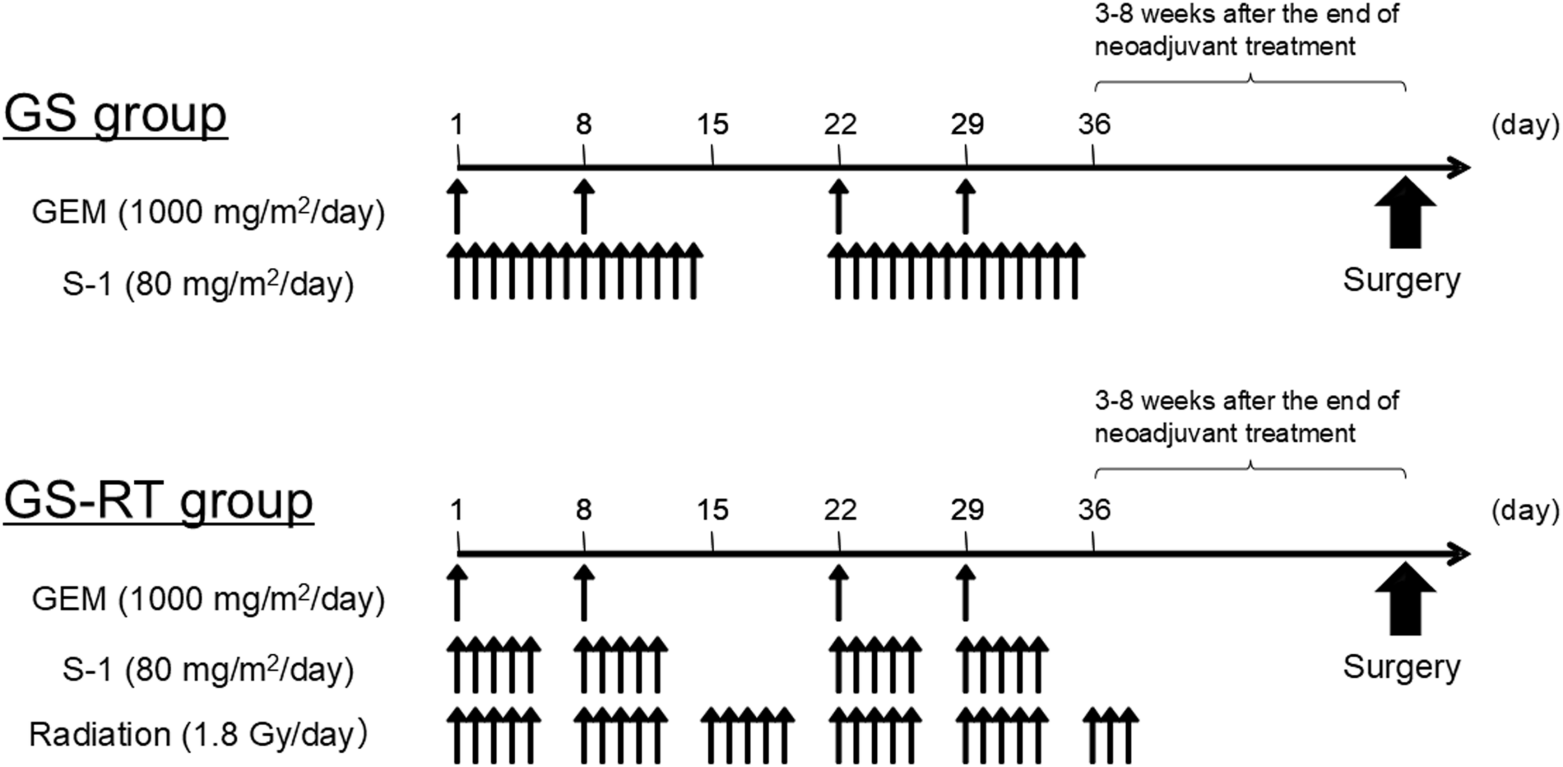
Treatment protocols planned for the study. Arrows indicate administration time points within each treatment arm. GEM, gemcitabine; GS, gemcitabine and S-1; GS-RT, gemcitabine and S-1 with concurrent radiotherapy.

Eligible patients will be randomly assigned in a 1:1 ratio to the GS or GS-RT treatment arm. The regimens of neoadjuvant GS and GS-RT were determined based on previous studies [5,11,14]. The treatment protocols are visualized in Fig 1. Briefly, patients randomized to the GS arm will receive two cycles of neoadjuvant chemotherapy with 1000 mg/m² intravenous gemcitabine on days 1, 8, 22, and 29 and 80 mg/m²/day oral S-1 administered in two divided doses after breakfast and dinner on days 1−14 and 22−35. In contrast, patients randomized to the GS-RT arm will receive two cycles of neoadjuvant chemotherapy with 1000 mg/m² intravenous gemcitabine on days 1, 8, 22, and 29 and 80 mg/m²/day oral S-1 administered on days 1−5, 8−12, 22−26, and 29−33 with concurrent radiotherapy at a total dose of 50.4 Gy in 28 fractions (1.8 Gy/day, 5 days per week, excluding weekends and holidays) to the pancreatic primary tumor and involved regional lymph nodes, including elective nodal irradiation. The radiotherapy will be delivered using 3D-chemoradiotherapy or intensity modulated radiation therapy with ≥6 MV photons. Target volumes will be defined on contrast-enhanced computed tomography (CT)/magnetic resonance imaging. Depending on the individual tumor site and its extension, patients in both groups will undergo curative-intent pancreatectomy with regional node dissection. Surgery will be performed approximately 3–8 weeks after completion of neoadjuvant GS or GS-RT provided that no disease progression or contraindication to surgery is identified. The protocol does not define postoperative treatment; however, adjuvant chemotherapy with S-1 is recommended based on the Japanese clinical practice guidelines [6].

### Endpoints

The primary endpoint is OS, defined as the time from randomization to death from any cause. Secondary endpoints are resection rate, defined as the proportion of patients who receive resection among those assigned to each group; R0 resection rate, defined as the proportion of patients with histologically confirmed margin-negative resection among patients with resection; histological tumor response evaluated using standardized pathological grading systems; progression-free survival (PFS), defined as the time from randomization to disease progression or death; and adverse events, defined as the incidence of grade 3 or higher treatment-related toxicities evaluated according to CTCAE v5.0 [15,16].

### Sample size and statistical considerations

We assume 3-year survival rates of 35% in the GS-treated group and 55% in the GS-RT-treated group based on previous studies [11,14]. With a follow-up period of 3 years, two-sided significance level of α = 0.05, and power of 1–β = 0.80, the required sample size for detecting a significant difference between the two groups by the log-rank test is calculated to be 91 patients per group. Considering an anticipated dropout rate of approximately 10%, the target sample size (number of participants to be randomized) is 100 patients per group, for a total of 200 patients.

Kaplan-Meier curves will be plotted for OS and PFS, and comparisons will be made between groups using the stratified log-rank test. Adjusted hazard ratios and 95% confidence intervals will be estimated using the Cox proportional hazards model. Subgroup analyses to examine the effects of specific characteristics before neoadjuvant therapy will be performed in the same manner. Binary endpoints, including resection rate, R0 resection rate, histological response, and grade ≥3 adverse events, will be analyzed using logistic regression models adjusted for the stratification factor, baseline CA19−9 level (<370 vs. ≥ 370 U/mL), with estimation of odds ratios and 95% confidence intervals. Missing data will not be imputed. For time-to-event endpoints, patients without an event at the end of the observation period will be censored at the date of last confirmed survival or non-progression status. For secondary endpoints other than time-to-event outcomes, handling of patients lost to follow-up will depend on the timing of discontinuation. For resection rate, R0 resection rate, and histological response, patients lost to follow-up before surgery will be excluded from the analysis, those lost during surgery will be considered as non-resected cases, and those lost after surgery will be included in the analysis. For grade ≥3 adverse events, all patients, including those lost to follow-up, will be included in the analysis. No interim analysis is planned for this study. Multiplicity across secondary endpoints will not be adjusted for. In addition to the primary analysis, a supplementary analysis will be performed for the population defined according to the treatment actually allocated. All statistical tests will be two-sided, and a p-value < 0.05 will be considered statistically significant. All analyses will be conducted using JMP available at the time the analyses are performed (SAS Institute Inc., Cary, NC, USA).

### Participant timeline

**Fig 2:**
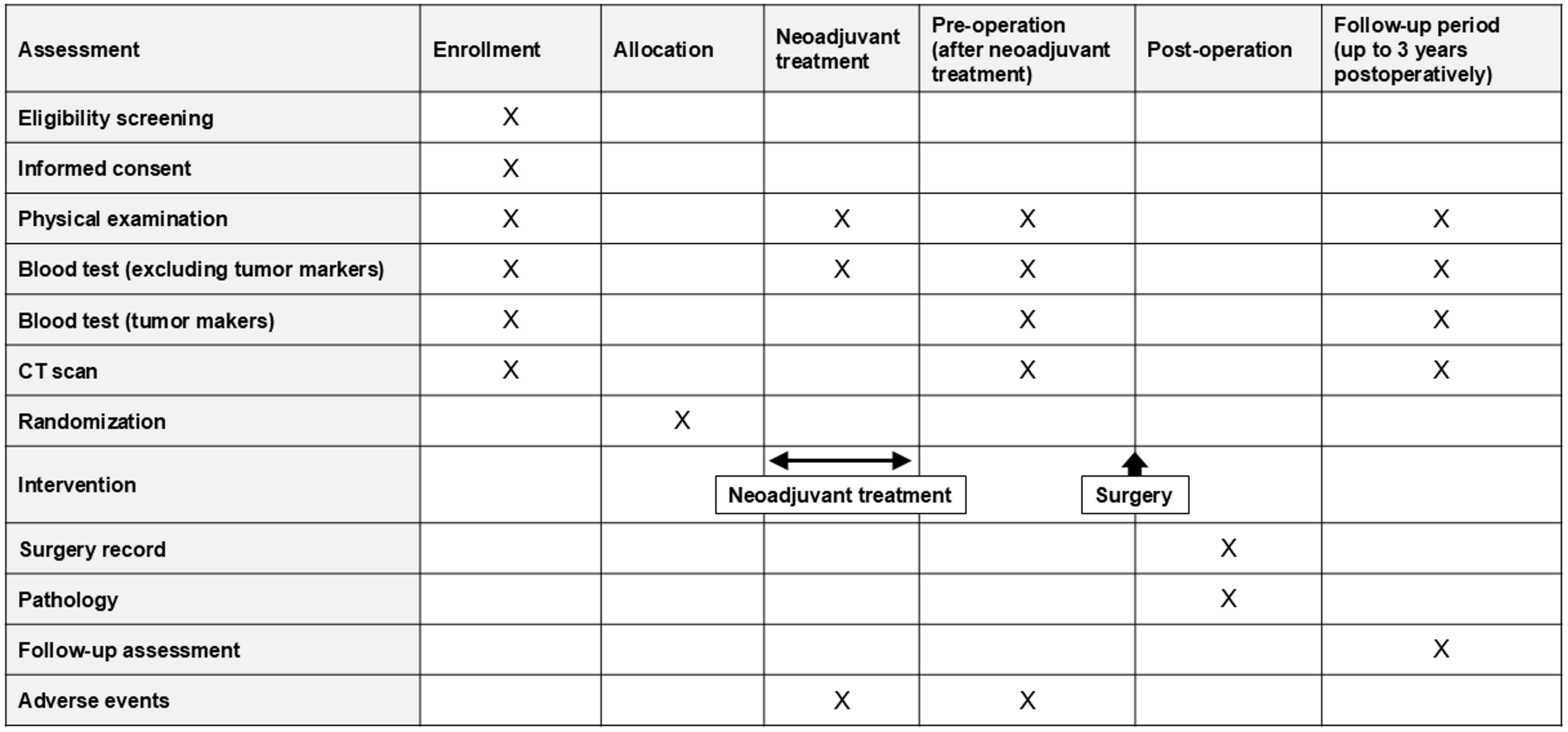
Participant timeline. This figure summarizes participant timeline throughout the study period, including enrollment, neoadjuvant gemcitabine and S-1 (GS or GS-RT) treatment, preoperative evaluation, surgery, and postoperative follow-up.GS, gemcitabine and S-1; GS-RT, gemcitabine and S-1 with concurrent radiotherapy.

After completion of the neoadjuvant treatment, patients will undergo curative-intent surgery followed by postoperative follow-up for up to 3 years. The time schedule of enrollment, interventions, and assessments is presented in Fig 2.

### Study Progress

Recruitment for this study began on 1 December 2025 and is still ongoing. The recruitment is expected to be completed on 30 November 2027. Follow-up for all participants will continue for up to 3 years after the registration of the final participant. Data analysis will begin following the completion of the 3-year follow-up, and results are expected to be available by 2031.

## Discussion

This CSGO-HBP-027 trial is a multicenter randomized controlled trial designed to optimize neoadjuvant treatment in patients with resectable PDAC. Although both chemotherapy and chemoradiotherapy have been reported to be effective, no large trials have directly compared the two strategies for resectable PDAC by assigning two arms with nearly identical chemotherapeutic regimens. Therefore, the results of this study will be helpful for determining the optimal neoadjuvant strategy and provide an advantage in evaluating the effect of radiotherapy in patients with resectable PDAC.

However, several reports have compared neoadjuvant chemotherapy and chemoradiotherapy in PDAC patients. First, the ESPAC5 trial, a four-arm randomized phase II study, demonstrated that neoadjuvant therapy with chemotherapy alone or chemoradiotherapy improved OS in patients with borderline resectable PDAC. Interestingly, though the included patients exhibited borderline resectable PDAC, patients treated with chemotherapy had better survival than those treated with chemoradiotherapy. However, the chemotherapy regimens were gemcitabine and capecitabine or mFOLFIRINOX in the chemotherapy arm, whereas capecitabine alone was used in the chemoradiotherapy arm, so the independent benefit of radiotherapy could not be clearly determined. Similarly, the Dutch PREOPANC-2 trial compared chemotherapy and chemoradiotherapy and showed no significant difference in outcomes [17]. However, this trial also included both resectable and borderline resectable PDAC patients and, again, the chemotherapy regimens differed between the two groups; thus, the incremental benefit of radiotherapy remained uncertain. In Japan, the JASPAC 04 study recently demonstrated no significant prognostic difference between preoperative chemotherapy and chemoradiotherapy [18]. To the best of our knowledge, this has been the only study to compare chemotherapy and chemoradiotherapy only among patients with resectable PDAC. In this study, the chemotherapy arm used GS, whereas the chemoradiotherapy arm used S-1 alone. Therefore, although the chemotherapy regimens in the arms were more similar than in the previous trials, they were still not identical, and the true additive effect of radiotherapy could not be fully evaluated. Furthermore, the GABARNANCE trial, a randomized phase II/III study in patients with borderline resectable PDAC, compared neoadjuvant gemcitabine and nab-paclitaxel with concurrent chemoradiotherapy using S-1 [19] and demonstrated no significant difference in OS between the two groups. Similar to other trials, the chemotherapy regimens differed between the treatment arms, which made it challenging to determine the specific role of radiotherapy. Thus, no previous reports have compared chemotherapy and chemoradiotherapy using the same chemotherapy regimen, and only one study has focused exclusively on patients with resectable PDAC. In contrast, the study presented here will focus only on resectable PDAC. Furthermore, in the previous trials, chemotherapy in the chemoradiotherapy arm consisted of a single agent, such as gemcitabine, capecitabine, or S-1 alone, which is generally weaker than the regimen used in the chemotherapy arm, whereas GS-RT in the study described here combines two agents, making the regimens nearly equivalent between the chemotherapy and chemoradiotherapy arms. This is noteworthy because, although resectable PDAC is considered a systemic disease, as suggested by the previously reported incidence of postoperative distant metastasis, it is thought to be more localized than borderline resectable PDAC in terms of local tumor progression. Therefore, in trials comparing chemoradiotherapy with chemotherapy for resectable PDAC, it would be desirable to design a chemoradiotherapy regimen that does not attenuate the effect of chemotherapy and could facilitate identifying the most optimal neoadjuvant regimen. The results would also contribute to evaluating the additive effect of radiotherapy. Accordingly, the design of the present study not only allows determination of the optimal neoadjuvant strategy for resectable PDAC, but it enables evaluation of the incremental effect of radiotherapy in this specific population.

Our study includes multicenter participation, which will aid in collecting generalizable data. Moreover, the participating institutions are closely affiliated with each other through personnel exchange, and the surgeons have received standardized training in surgical techniques with regular video-based surgical conferences, ensuring little variation in operative procedures among the centers. Similarly, radiotherapy is standardized across institutions through personnel exchange and shared protocols. These features help overcome one of the typical disadvantages of multicenter studies. However, there are also some limitations to the planned study. First, the study will be conducted only in Japan, where GS is the standard neoadjuvant regimen. Therefore, the results may not be directly applicable to other countries where different regimens, such as FOLFIRINOX, are commonly used. In addition, the open-label design may introduce bias in perioperative management or reporting of adverse events, but this is unavoidable because of the clear differences in preoperative treatments. That said, we consider this limitation acceptable by adopting OS as the primary endpoint, which is an objective measure and, therefore, less prone to such bias; however, caution is warranted when interpreting the results.

In summary, the CSGO-HBP-027 trial has the potential to redefine neoadjuvant treatment strategies for resectable PDAC. If GS-RT shows a survival benefit over GS alone, chemoradiotherapy could become the new standard neoadjuvant treatment for resectable PDAC. If no significant difference is found, the results will still provide high-quality evidence supporting GS alone as an effective standard of care. Therefore, the findings are expected to have a direct impact on clinical practice.

## Supporting information

S1 Checklist SPIRIT checklist. (DOCX)

S1 File Study protocol English, revised after acceptance. (DOCX)

S2 File Study protocol Japanese, revised after acceptance. (DOCX)
