# Supplementary material for: Study protocol: A randomized controlled trial of chemoradiotherapy versus chemotherapy as neoadjuvant therapy for resectable pancreatic cancer (CSGO-HBP-027)
Source: PLoS One. 2026 Mar 26;21(3):e0345459. doi: 10.1371/journal.pone.0345459 (PMC13020802; doi:10.1371/journal.pone.0345459)
Supplement: S1 File — (DOCX) [file pone.0345459.s002.docx]

**Study Protocol**

**A Randomized Controlled Trial of Chemoradiotherapy Versus Chemotherapy as Neoadjuvant Therapy for Resectable Pancreatic Cancer (CSGO-HBP-027)**

**Overall Administrator:**

**********

**Protocol Number:** CSGO-HBP-027

**Date of Creation: February 26, 2026 Version 1.3**

**1. Abbreviations and Definitions**

**1.1. Abbreviations**

| **Abbreviation** | **Full Term** |
| --- | --- |
| ALT | Alanine aminotransferase |
| AST | Aspartate aminotransferase |
| ALP | Alkaline phosphatase |
| GEM | Gemcitabine |

**1.2. Definitions of Terms**

**(1) Overall Administrator**

Person who oversees and manages the implementation of clinical research among those conducting clinical research as stipulated by the Clinical Research Act.

**(2) Principal Investigator**

Physician who, among those conducting clinical research as stipulated by the Clinical Research Act, is responsible for overseeing the tasks related to clinical research at the implementing medical institution.

**(3) Sub-investigator**

Physician who, at the implementing medical institution, shares the tasks related to clinical research under the guidance of the principal investigator.

**(4) Monitoring**

Investigation conducted by a person designated by the overall administrator to ensure that clinical research is being conducted appropriately from the perspective of ensuring the reliability of clinical research and protecting research participants, by checking the progress of this study and whether this study is being conducted in accordance with the Clinical Research Act, its enforcement regulations, and the research protocol.

**(5) Research Collaborator**

Pharmacists, nurses, and other medical professionals who, at the implementing medical institution, cooperate with the tasks related to the research of the principal investigator or sub-investigators under their guidance.

**2. Background of the Study**

**2.1. Target Disease Name**

Pancreatic Cancer

**2.2. Concept of the Target Disease**

Pancreatic cancer is a disease with a poor prognosis, with a 5-year survival rate of 11.8% [1], as early diagnosis is difficult and it is often advanced at the time of diagnosis. The only curative treatment for pancreatic cancer is surgical resection, but only about 20% of patients are eligible [2]. Even in resected cases, the treatment outcomes are extremely poor due to early local recurrence and distant metastatic recurrence. Therefore, there is an expectation for multidisciplinary treatment centered on surgery, rather than surgery being the sole treatment.

**2.3. Standard Treatment for Resectable Pancreatic Cancer**

According to the Pancreatic Cancer Clinical Practice Guidelines (2022 edition) [3], surgical resection is considered the standard treatment for resectable pancreatic cancer if the patient's condition allows. However, pancreatic cancer has a high postoperative recurrence rate and poor prognosis even after resection. Therefore, the combination of adjuvant therapy in addition to resection has been attempted, and the prognostic improvement effect of neoadjuvant chemotherapy for resectable pancreatic cancer has been proven in randomized controlled trials, establishing these multidisciplinary treatments as standard therapy. A randomized controlled trial conducted in Japan (Prep-02 trial) showed that the overall survival rate in the group that received neoadjuvant chemotherapy with gemcitabine hydrochloride (GEM) and tegafur-gimeracil-oteracil potassium combination (S-1) was significantly better compared to the upfront surgery group [4]. Therefore, the pancreatic cancer clinical practice guidelines propose the use of GEM+S-1 combination therapy (GS therapy) as neoadjuvant chemotherapy for resectable pancreatic cancer. On the other hand, while neoadjuvant chemoradiotherapy, which is the subject of this study, is proposed for borderline resectable pancreatic cancer in the pancreatic cancer clinical practice guidelines, it is not mentioned as a treatment for resectable pancreatic cancer. However, a prospective study (CSGO-HBP-003) [5] from multiple institutions (Osaka University Digestive Surgery Collaborative Research Group) centered on Osaka University Digestive Surgery has investigated the treatment outcomes of neoadjuvant chemoradiotherapy (GS-RT therapy) combining GS therapy with radiation therapy. Although a direct comparison is difficult due to the single-arm study design, favorable results have been reported, suggesting its potential usefulness even for resectable pancreatic cancer. Furthermore, a recent comparative study of the outcomes of GS-RT therapy and GS therapy based on prognostic information of patients enrolled in two trials (CSGO-HBP-003 and CSGO-HBP-015 [6]) conducted by the Osaka University Digestive Surgery Collaborative Research Group showed that the outcomes of GS-RT therapy were significantly better than those of GS therapy (3-year survival rate: approximately 55% vs. approximately 35%) (manuscript submitted for publication). This indicates the possibility that neoadjuvant chemoradiotherapy could become a standard treatment for resectable pancreatic cancer.

**2.4. Investigational Drugs in This Study**

GEM is an anticancer drug classified as an antimetabolite. It is metabolized to triphosphate in cells, inhibiting DNA synthesis. Furthermore, triphosphate concentrations are maintained for a long time in cells, exhibiting strong cytotoxic effects against solid tumors. GEM is widely used worldwide as a first-line anticancer drug for advanced pancreatic cancer. A Phase I trial was conducted in Japan, and its indication for pancreatic cancer was approved in April 2001. In addition to pancreatic cancer, it is indicated for non-small cell lung cancer, biliary tract cancer, urothelial cancer, unresectable or recurrent breast cancer, ovarian cancer that has progressed after chemotherapy, and recurrent or refractory malignant lymphoma. S-1 is an oral anticancer drug that combines tegafur, a prodrug of 5-FU, with gimeracil and oteracil potassium as modulators. It was developed with the aim of increasing the blood concentration of 5-FU to enhance its antitumor effect and to reduce the associated gastrointestinal toxicity. In a preliminary Phase II study (n=19) for advanced pancreatic cancer with distant metastasis, the response rate was 21.1%, time to progression (TTP) was 77 days, and median survival time (MST) was 169 days. In a later Phase II study (n=40), the response rate was 37.5%, TTP was 113 days, and MST was 281 days, leading to its approval for pancreatic cancer in August 2006. It is also indicated for gastric cancer, colorectal cancer, head and neck cancer, non-small cell lung cancer, unresectable or recurrent breast cancer, pancreatic cancer, biliary tract cancer, and postoperative drug therapy for hormone receptor-positive and HER2-negative breast cancer with a high risk of recurrence. It is known from preclinical studies that 5-FU, a metabolite of S-1, and GEM inhibit DNA synthesis through different pathways and have synergistic effects [7]. Many clinical trials have reported the safety and relatively good response rates (10-20%) and survival times (median: 7-10 months) of 5-FU and GEM combination therapy for pancreatic cancer [8] [9].

**2.5. Significance of Conducting This Study**

As mentioned above, an updated comparison of prognostic information from patients enrolled in two trials (CSGO-HBP-003 and CSGO-HBP-015) conducted by the Osaka University Digestive Surgery Collaborative Research Group showed that the outcomes of GS-RT therapy were significantly better than those of GS therapy (3-year survival rate: approximately 55% vs. approximately 35%). Given the potential for neoadjuvant chemoradiotherapy to become a standard treatment for resectable pancreatic cancer, this study will conduct a randomized controlled trial to compare the efficacy of GS therapy and GS-RT therapy.

**3. Research Objectives and Endpoints**

**3.1. Research Objective**

To prospectively evaluate the efficacy of GS-RT therapy as neoadjuvant treatment for resectable pancreatic cancer, using GS therapy as a control.

**3.2. Primary Endpoint**

Overall Survival (OS)

**[Rationale for Primary Endpoint Setting]**

Overall survival was chosen as it is the true endpoint of cancer treatment.

**3.3. Secondary Endpoints**

(1) Resection rate (= number of resected cases / number of allocated cases × 100)

(2) R0 resection rate (= number of R0 resected cases / number of resected cases × 100)

(3) Histopathological effect of neoadjuvant treatment

(4) Progression-Free Survival (PFS)

(5) Adverse events of Grade 3 or higher

**[Rationale for Secondary Endpoints Setting]**

(1) This was set to compare the number of cases that underwent resection between groups, as it is anticipated that some patients, despite being eligible for resection and enrolled in this study, may be unable to proceed with planned resection due to worsening condition during neoadjuvant treatment (e.g., progression of pancreatic cancer or adverse events from neoadjuvant therapy), or may have to abort resection during surgery due to findings such as progression of pancreatic cancer identified intraoperatively. (2) This was set to compare the number of cases that achieved R0 resection between groups.

(3) This was set to compare the histopathological effects of neoadjuvant treatment between groups.

(4) This was set to compare progression before surgery and recurrence after surgery between groups.

(5) This was set to evaluate safety.

**4. Study Design**

**4.1. Study Design**

A multi-center, randomized, open-label, parallel-group comparative study with the following two groups: (1) Chemotherapy (GS) group

(2) Chemoradiotherapy (GS-RT) group

For allocation, stratified randomization will be applied using pre-treatment CA19-9 values (<370, ≥370 U/mL) as a stratification factor.

**[Rationale for Study Design Setting]**

Randomized allocation will be performed to minimize bias in patient characteristics between groups and enhance comparability. Pre-treatment CA19-9 values are considered a factor influencing efficacy evaluation as they reflect tumor burden, hence they were chosen as a stratification factor.

**4.2. Planned Number of Study Participants**

A total of 200 participants will be randomized. Chemotherapy group: 100 cases Chemoradiotherapy group: 100 cases

**[Rationale for Planned Number of Study Participants Setting]**

The prognostic information of patients enrolled in two studies (CSGO-HBP-003 and CSGO-HBP-015) for pancreatic cancer, conducted by the Osaka University Digestive Surgery Collaborative Research Group, was referenced. Based on these studies, assuming a 3-year survival rate of 35% for the chemotherapy group and 55% for the chemoradiotherapy group, with a follow-up period of 3 years, a significance level α = 0.05 (two-sided), and a power of 1-β = 0.80, the sample size required for statistical significance by the log-rank test was calculated to be 91 cases per group. Therefore, considering a dropout rate of approximately 10%, the target number of cases (number of randomized study participants) was set at 100 cases per group, totaling 200 cases for both groups. The sample size calculation was performed using SAS version 9.4.

**4.3. Planned Study Period**

(1) Planned Research Period From the jRCT publication date to August 31, 2031

(2) Planned Enrollment Period From the jRCT publication date to August 31, 2027

(3) Planned Observation Period From the jRCT publication date to August 31, 2030

All cases will be followed up for three years after the registration date of the last enrolled case.

**5. Selection of Study Participants**

Patients who meet all inclusion criteria and do not violate any exclusion criteria will be selected as study participants.

**5.1. Inclusion Criteria**

(1) Patients with histologically or cytologically confirmed conventional pancreatic adenocarcinoma.

(2) Age 18 years or older at the time of informed consent.

(3) ECOG PS of 0-1.

(4) Patients judged to have resectable pancreatic cancer based on imaging studies (definition of resectable pancreatic cancer follows NCCN Guidelines Version 2.2025). The possibility of curative resection will be evaluated using CT scans with a slice thickness of at least 5mm or less. MRI, ultrasonography, PET/CT, laparoscopy, etc., will be performed as necessary. Patients with the following imaging findings will be diagnosed with resectable pancreatic cancer:

① No tumor contact or invasion of the portal vein/superior mesenteric vein, or contact/invasion is less than 180 degrees and amenable to concomitant resection.

② No contact or invasion of the inferior vena cava.

③ No tumor contact or invasion of the aorta, celiac artery, common hepatic artery, or superior mesenteric artery.

(5) Patients receiving this treatment as their first treatment for pancreatic cancer.

(6) Patients expected to survive for 6 months or more from the time of informed consent.

(7) Patients with no tumor invasion into the gastrointestinal tract.

(8) Patients with preserved major organ function (bone marrow, liver, kidney, lung, etc.): ・White blood cell count: ≥ 3,500/mm³, Neutrophil count: ≥ 2,000/mm³, Platelet count: ≥ 100,000/mm³, Hemoglobin: ≥ 9.0 g/dL ・Total bilirubin: ≤ 2.0 mg/dL (However, for cases undergoing biliary drainage for obstructive jaundice, ≤ 3.0 mg/dL) ・AST (GOT), ALT (GPT): ≤ 150 U/L ・Serum creatinine: ≤ 1.2 mg/dL, Creatinine clearance: ≥ 60 mL/min (estimation by Cockcroft-Gault formula is acceptable)

(9) Patients who can provide written informed consent for participation in this study.

**[Rationale for Inclusion Criteria Setting]**

Criteria (1) to (7) were set to select appropriate study participants for evaluating the efficacy of neoadjuvant treatment. Criterion (8) was set to minimize the risk to study participants. Criterion (9) was set to ensure that the study is conducted with participants who have given proper consent.

**5.2. Exclusion Criteria**

(1) Cases of unresectable pancreatic cancer meeting the following:

① Distant organ metastasis

② Positive peritoneal dissemination, positive peritoneal cytology

③ Para-aortic lymph node metastasis

④ Contact or invasion of the portal vein/superior mesenteric vein of 180 degrees or more

⑤ Contact or invasion of any of the aorta, superior mesenteric artery, celiac artery, or common hepatic artery

(2) Patients with pulmonary fibrosis or interstitial pneumonia, a history thereof, or CT findings suggestive of these conditions. Patients with severe pulmonary emphysema or old inflammatory changes, or significantly impaired respiratory function in pre-treatment respiratory function tests (e.g., %VC ≤ 50%, FEV1 < 1L).

(3) Patients with active infection (excluding cholangitis and viral hepatitis).

(4) Patients with severe comorbidities (e.g., heart failure, renal failure, liver failure, hemorrhagic peptic ulcer, paralytic ileus, intestinal obstruction, uncontrolled diabetes).

(5) Patients with moderate or severe ascites or pleural effusion (requiring drainage, etc.).

(6) Patients with active synchronous or metachronous (disease-free interval within 2 years) multiple primary cancers. However, carcinoma in situ or mucosal carcinoma equivalent lesions considered cured by local treatment are not considered active multiple primary cancers.

(7) Patients using flucytosine, phenytoin, or warfarin potassium.

(8) Women who are pregnant, potentially pregnant, wish to become pregnant, or are breastfeeding. Men who wish to father a child.

(9) Patients with severe drug hypersensitivity.

(10) Patients currently participating in other interventional studies.

(11) Patients deemed unsuitable as study participants by the principal investigator or sub-investigator for any other reason.

**[Rationale for Exclusion Criteria Setting]**

Criterion (1) was set to exclude study participants inappropriate for evaluating the efficacy of neoadjuvant treatment. Criteria (2) to (5), (8), and (9) were set to ensure the safety of study participants. Criterion (6) was set because it could affect the evaluation of efficacy. Criterion (7) was set because these are contraindicated or cautionary concomitant drugs with S-1, to ensure the safety of study participants. Criterion (10) was set because it could have unpredictable effects on this study and the ongoing interventional study. Criterion (11) was set to exclude individuals deemed unsuitable for this study by the principal investigator or sub-investigator for reasons other than those listed above.

**6. Investigational Medicinal Products, etc.**

**6.1. Overview of Investigational Medicinal Products, etc.**

This study will use the following investigational drugs:

| **Regarding the Pharmaceutical and Medical Device Act (PMD Act)** | □ Unapproved ■ Off-label □ Approved |
| --- | --- |
| **Generic Name** | Gemcitabine Hydrochloride for Injection |
| **Brand Name** | Gemzar for Injection 200mg, Gemzar for Injection 1g, etc. |
| **Manufacturer/Distributor** | Eli Lilly Japan K.K., etc. |
| **Approval Number** | 21300AMY00405, 21300AMY00404, etc. |
| **Storage Method** | Room temperature |

| **Regarding the Pharmaceutical and Medical Device Act (PMD Act)** | □ Unapproved ■ Off-label □ Approved |
| --- | --- |
| **Generic Name** | Tegafur, Gimeracil, Oteracil Potassium Combination |
| **Brand Name** | TS-1 Combination OD Tablet T20, TS-1 Combination OD Tablet, etc. |
| **Manufacturer/Distributor** | Taiho Pharmaceutical Co., Ltd., etc. |
| **Approval Number** | 22500AMX00075000, 22500AMX00076000, etc. |
| **Storage Method** | Room temperature |

**6.2. Ensuring the Quality of Investigational Medicinal Products, etc.**

The overall administrator shall implement the following:

1. If information is obtained that the quality of the medicinal products, etc., used in the clinical study is poor, verification will be conducted, and the certified clinical research review committee will be reported on the measures to be taken, such as suspension of the clinical study. Records of this will also be created.
2. If it is determined that recall of the medicinal products, etc., is necessary due to reasons such as poor quality of the medicinal products, etc., used in the clinical study, the certified clinical research review committee will be promptly reported, and the following tasks will be performed: (a) Promptly notify the principal investigator of the discontinuation of use and recall of the medicinal products, etc. (b) Create and retain recall processing records detailing the content of the recall, the results of the root cause investigation, and corrective actions.

The principal investigator shall implement the following:

1. Conduct appropriate management of medicinal products, etc., based on their approved specifications.
2. If notified by the overall administrator, promptly instruct sub-investigators, etc., to discontinue use and recall the medicinal products, etc.

**7. Application Method of Investigational Medicinal Products, etc. to Study Participants**

In this study, GEM and S-1 administration and radiation therapy will be treated as protocol treatments, while surgery, post-treatment, supportive care, and concomitant therapies will be conducted as standard medical practice. Protocol treatment will commence within 21 days of the randomization date.

**7.1. Application Method of Investigational Medicinal Products, etc.**

**7.1.1. GEM**

(1) **GS Therapy Group**

Administer intravenously at a dose of 1,000 mg/m² based on body surface area on days 1, 8, 22, and 29. Refer to 7.1.4 and 7.1.5 for administration initiation/continuation criteria and dose reduction criteria (the same applies hereafter).

(2) **GS-RT Therapy Group**

Administer intravenously at a dose of 1,000 mg/m² based on body surface area on days 1, 8, 22, and 29.

**7.1.2. S-1**

(1) **GS Therapy Group**

Administer orally at a dose of 80 mg/m² based on body surface area, twice daily after breakfast and dinner, for one week during the GEM administration week. That is, oral administration on days 1-14 and 22-35. The 80 mg/m² dosage is as follows:

| **Body Surface Area** | **Standard Daily Dose** | **Morning - Evening** |
| --- | --- | --- |
| Less than 1.25 m² | 80 mg/day | 40 mg - 40 mg |
| 1.25 m² to less than 1.50 m² | 100 mg/day | 50 mg - 50 mg |
| 1.50 m² or more | 120 mg/day | 60 mg - 60 mg |

(2) **GS-RT Therapy Group** Administer orally at a dose of 80 mg/m² based on body surface area, twice daily after breakfast and dinner, for five days during the GEM administration week. That is, oral administration on days 1-5, 8-12, 22-26, and 29-33. The 80 mg/m² dosage is as follows:

| **Body Surface Area** | **Standard Daily Dose** | **Morning - Evening** |
| --- | --- | --- |
| Less than 1.25 m² | 80 mg/day | 40 mg - 40 mg |
| 1.25 m² to less than 1.50 m² | 100 mg/day | 50 mg - 50 mg |
| 1.50 m² or more | 120 mg/day | 60 mg - 60 mg |

**7.1.3. Rationale for Application Method of Investigational Medicinal Products, etc.**

Details of GS therapy were determined based on the Prep-02 trial [4] and CSGO-HBP-015 trial [6]. Details of GS-RT therapy were determined based on the CSGO-HBP-003 trial [5].

**7.1.4. Criteria for Initiation and Continuation of Neoadjuvant Chemotherapy**

(1) **Criteria for Chemotherapy Initiation on Day 1 and Day 22**

For chemotherapy initiation on Day 1 and Day 22, GEM and S-1 will be administered after confirming that laboratory values and clinical findings within 24 hours prior to GEM administration meet all the continuation criteria below.

**Criteria for Chemotherapy Initiation on Day 1 and Day 22**

| **Item** | **Day 1 Initiation Criteria** | **Day 22 Initiation Criteria** |
| --- | --- | --- |
| WBC count | ≥ 3,500/mm³ | ≥ 3,000/mm³ |
| Neutrophil count | ≥ 2,000/mm³ | ≥ 1,500/mm³ |
| Platelet count | ≥ 100,000/mm³ | ≥ 75,000/mm³ |
| AST | ≤ 150 U/L | ≤ 150 U/L |
| ALT | ≤ 150 U/L | ≤ 150 U/L |
| Total Bilirubin | ≤ 2.0 mg/dL | ≤ 2.0 mg/dL |
| Serum Creatinine | ≤ 1.2 mg/dL | ≤ 1.2 mg/dL |
| Diarrhea, Stomatitis | Grade 1 or less | Grade 1 or less |
| Other non-hematologic toxicities (excluding alopecia, diarrhea) | Grade 1 or less | Grade 2 or less |

(2) **Criteria for Continuation of Neoadjuvant Chemotherapy within a Course**

For chemotherapy administration on Day 8 and Day 29 within a course, GEM and S-1 will be administered after confirming that laboratory values and clinical findings within 24 hours prior to GEM administration meet all the in-course continuation criteria below. Additionally, examinations will be performed as needed during the course to confirm that the in-course continuation criteria are met before continuing administration.

**In-course Administration Continuation Criteria (Day 8, 29)**

| **Item** | **In-course Administration Continuation Criteria** |
| --- | --- |
| WBC count | ≥ 2,000/mm³ |
| Neutrophil count | ≥ 1,000/mm³ |
| Platelet count | ≥ 75,000/mm³ |
| Serum Creatinine | < 1.2 mg/dL |
| Diarrhea, Stomatitis | Grade 1 or less |
| Other non-hematologic toxicities (excluding alopecia, diarrhea) | Grade 2 or less |

**7.1.5. Dose Reduction Criteria**

Based on the occurrence of adverse events within the current course, administration may be resumed after a one-step dose reduction, following the dose modification criteria below and confirming that the above administration continuation criteria are met. If, in a case where the dose has already been reduced once according to the criteria below, adverse events requiring further dose reduction are observed, no further reduction will be made, and the study for that case will be terminated.

**Dose Modification Criteria**

| **Item** | **GEM** | **S-1** |
| --- | --- | --- |
| Neutrophil count < 500/mm³ (Grade 4) | 1-step reduction | 1-step reduction |
| Febrile neutropenia: Infection with Grade 3-4 neutropenia (with fever ≥ 38.5°C) | 1-step reduction | 1-step reduction |
| Infection with Grade 3 or 4 neutrophils (e.g., cholangitis) | 1-step reduction | 1-step reduction |
| Platelet count < 25,000/mm³ (Grade 4) | 1-step reduction | 1-step reduction |
| Serum Creatinine: 1.2-1.5 mg/dL | No reduction | 1-step reduction |
| Serum Creatinine: ≥ 1.5 mg/dL | Discontinue | Discontinue |
| Grade 3 or higher stomatitis, diarrhea | No reduction | 1-step reduction |
| Grade 3 or higher non-hematologic toxicities (excluding nausea/vomiting, anorexia, alopecia, fatigue) | 1-step reduction | 1-step reduction |
| Delayed recovery from adverse events, leading to postponement or skipping of GEM administration | 1-step reduction | No reduction |

(1) **GEM Dose Reduction Criteria**

| **Standard Dose** | **1-step Reduced Dose** |
| --- | --- |
| 1,000 mg/m² | 800 mg/m² |

(2) **S-1 Dose Reduction Criteria**

| **Body Surface Area** | **Standard Daily Dose** | **1-step Reduced Daily Dose** |
| --- | --- | --- |
| Less than 1.25 m² | 80 mg/day | 60 mg/day |
| 1.25 m² to less than 1.50 m² | 100 mg/day | 80 mg/day |
| 1.50 m² or more | 120 mg/day | 100 mg/day |

**7.2. Radiation Therapy**

**7.2.1. Radiation Dose and Irradiation Method**

Radiation therapy will be administered at 1.8 Gy once daily, for a total of 50.4 Gy over 28 consecutive days, excluding weekends (Saturday, Sunday, holidays). If continuous irradiation is not possible due to holidays, etc., irradiation will be postponed to ensure the total dose. However, postponement of radiation therapy will be limited to two weeks. In cases of clear disease progression (including worsening clinical symptoms), irradiation will be discontinued.

(1) **Start Time and Interruption Period, etc.**

Radiation therapy will start within 3 days after the start of chemotherapy. The start of radiation therapy can be on any day of the week. No planned interruption period will be set. If irradiation is not possible due to public holidays, it will be postponed to the next treatment day, and the dose will not be changed.

(2) **Dose and Fractionation**

1.8 Gy per fraction, once daily, 5 days a week, for a total of 28 fractions, total dose of 50.4 Gy, total treatment period of 38 days, and an allowable total treatment period of 60 days.

(3) **Radiation Therapy Equipment**

X-ray generating equipment of 6 MV or higher will be used.

(4) **Target Volume**

**Gross Tumor Volume (GTV):**

Set with reference to contrast-enhanced CT or MRI images. GTV primary: Primary tumor. GTV node: Lymph nodes with a short diameter of 1 cm or more.

**Clinical Target Volume (CTV):** CTV primary: GTV primary + 0.5 cm, considering subclinical extension. CTV node: Same as GTV node. CTV ENI: Posterior pancreatic tissue, para-aortic/celiac axis/common hepatic artery/superior mesenteric artery lymph node regions. ・Posterior pancreatic tissue: Dorsal boundary is 1 cm dorsal to the anterior vertebral body. ・Para-aortic lymph node region: 1 cm radius around the aorta, cranial to 0.5 cm cranial to the celiac artery root, caudal to the inferior border of the left renal vein. ・Celiac axis, common hepatic artery, superior mesenteric artery: 0.5 cm radius around these structures. The long axis of the superior mesenteric artery extends from its root to the branching point of the middle colic artery.

**Internal Target Volume (ITV):** ITV: CTV plus an appropriate margin considering respiratory motion (can be omitted for respiratory-gated irradiation).

**Planning Target Volume (PTV):** PTV: ITV plus an appropriate margin considering patient setup reproducibility errors, etc. (for respiratory-gated, CTV + margin).

(5) **Irradiation Method**

50.4 Gy will be delivered to the PTV using conventional fractionation (1.8 Gy × 5 fractions/week). A multi-leaf collimator (MLC) will be used for field shaping. Multi-port irradiation with 3 or more ports or rotational irradiation (3D-CRT, IMRT, VMAT) will be used.

(6) **Dose Distribution Calculation**

a) Target Reference Point/Prescription Point For 3D-CRT: Intersection on the central axis of the beam. For IMRT: PTV D95.

b) Dose Homogeneity within PTV and Dose Constraints for Organs at Risk ・PTV: D98% > 90% and D2% < 110% ・Spinal cord: Dmax < 45 Gy ・Kidney: V18Gy < 35% ・Liver: V30Gy < 40% and V20Gy < 67%

c) Dose Distribution Map Treatment planning will be performed using treatment planning CT. A dose distribution map of the transverse plane including the target reference point will be saved. Heterogeneity correction will be applied for monitor unit calculation. The treatment planning system and calculation algorithm will be recorded.

(7) **Treatment Planning CT**

Imaging range: Sufficiently includes the liver to both kidneys. Contrast agent: Use if possible. Slice thickness: ≤ 5 mm. Respiration: Image during natural breathing. Two phases (natural inspiration and natural expiration) or 4D-CT are recommended for respiratory motion evaluation.

(8) **Image Registration**

Image registration using the treatment device will be performed at the start of treatment and when the irradiation method is changed. It is recommended to perform image registration before each treatment and irradiate (IGRT).

**7.2.2. Radiation Irradiation/Interruption Criteria and Resumption Criteria**

The criteria for starting and continuing radiation irradiation within a course are as follows. If these criteria are not met, irradiation will be suspended until recovery, and resumed after confirming that the irradiation criteria are met. In addition, if the attending physician determines that irradiation is not possible, irradiation will be suspended. During radiation irradiation suspension, S-1 and GEM administration will also be suspended. After resuming radiation irradiation, the planned irradiation dose will be administered to ensure the total dose (50.4 Gy / 28 fractions). The suspension of radiation irradiation will be limited to a maximum of two weeks.

**In-course Radiation Therapy Irradiation Criteria**

| **Item** | **Radiation Irradiation Criteria** | **Resumption Dose after Recovery** |
| --- | --- | --- |
| WBC count | ≥ 1,000/mm³ (Grade 3 or less) | Prescribed dose |
| Neutrophil count | ≥ 500/mm³ (Grade 3 or less) | Prescribed dose |
| Platelet count | ≥ 25,000/mm³ | Prescribed dose |
| AST, ALT | < 150 IU | Prescribed dose |
| Radiation-induced dermatitis | Grade 1 or less | Prescribed dose |
| Other non-hematologic toxicities (excluding alopecia, diarrhea) | Grade 2 or less | Prescribed dose |

**7.2.3. Radiation Dose Reduction**

As a general rule, radiation dose reduction will not be performed.

**7.2.4. Administration Pattern of GEM+S-1 Combination Radiation Therapy**

**Example 1) Day 8: Administration withheld due to Grade 2 thrombocytopenia, recovered on Day 10.**

If the in-course administration continuation criteria are met within 3 days, including Day 8, administration will be resumed. However, the prescribed administration schedule will not be changed.

**Example 2) Day 8: Administration withheld due to Grade 2 thrombocytopenia, not recovered on Day 10.**

If the in-course administration continuation criteria are not met within 3 days, including Day 8, administration will be resumed on Day 22 as scheduled, after confirming that the initiation criteria are met.

**Example 3) Day 22: Administration withheld due to Grade 3 thrombocytopenia, recovered on Day 24.**

If the in-course administration continuation criteria are met within 3 days, including Day 22, administration will be resumed. However, the prescribed administration schedule for TS-1 will not be changed. Also, GEM administration for weeks 4 and 5 will be spaced at least 7 days apart.

**Example 4) Day 22: All treatments, including radiation, suspended due to Grade 3 leukopenia, recovered on Day 36.**

If the radiation irradiation criteria or Day 22 chemotherapy initiation criteria are met within 2 weeks after radiation therapy suspension, radiation and chemotherapy administration will be resumed according to the prescribed administration schedule. However, radiation therapy suspension will be limited to a maximum of 2 weeks.

**7.3. Surgery**

Curative surgery is planned, in principle, within 3 to 8 weeks from the last day of anticancer drug administration. The eligibility criteria for surgery are as follows. If a long time is required for eligibility recovery and the treating physician determines that surgery is dangerous, the protocol will be terminated, and subsequent treatment will be left to the discretion of the attending physician. Even if the planned chemotherapy cannot be performed due to patient preference or toxicity, surgery may be performed after confirming that the following surgical eligibility criteria are met.

**Surgical Eligibility Criteria**

Surgery is eligible if all of the following are met based on pre-operative imaging findings, measurement data, etc.: No unresectable factors on imaging.

① PS 0-1 (ECOG grade) ② WBC count: ≥ 2,000/mm³ ③ Neutrophil count: ≥ 1,000/mm³ ④ Platelet count: ≥ 100,000/mm³ ⑤ Total bilirubin: ≤ 2.0 mg/dL (However, for cases undergoing biliary drainage for obstructive jaundice, ≤ 3.0 mg/dL) ⑥ No signs of infection such as elevated body temperature, WBC, CRP. However, cases with signs of infection or elevated body temperature, WBC, CRP believed to be caused by the tumor are eligible.

For surgical procedures, patients will be re-explained, and written consent will be obtained using the prescribed form of each facility.

**Evaluation of Curative Resection Possibility**

Curative resection is considered possible if resectable pancreatic cancer or borderline resectable pancreatic cancer is diagnosed based on CT scans with a slice thickness of at least 5mm or less (definition of resectable pancreatic cancer and borderline resectable pancreatic cancer follows NCCN Guidelines Version 2.2025). MRI, ultrasonography, PET/CT, laparoscopy, etc., will be performed as necessary. The judgment of whether curative resection is possible based on imaging examinations will be left to the diagnostic criteria of radiologists, surgeons, and internists at each facility.

**7.4. Post-treatment**

Postoperative adjuvant therapy recommends oral S-1 in accordance with the Pancreatic Cancer Clinical Practice Guidelines (2022 edition), but no specific postoperative adjuvant chemotherapy is stipulated, respecting patient conditions and the attending physician's judgment. However, its content will be reported in the case report form. If protocol treatment is discontinued, no post-treatment is stipulated, but its content will be reported in the case report form.

**7.5. Supportive Care and Concomitant Therapies**

**7.5.1. Permitted Concomitant Therapies**

Concomitant use of therapeutic drugs for complications such as hypertension and diabetes, and for symptomatic treatment of adverse events, is permitted.

**7.5.2. Prohibited Concomitant Therapies**

During protocol treatment, until tumor progression or secondary cancer is confirmed, concomitant therapies other than GEM and S-1, hormone therapy other than steroid therapy, and immunotherapy will not be performed. Investigational drugs will also not be used concomitantly.

**7.5.3. Recommended Supportive Care**

The following supportive therapies are recommended. If administered, the drug name, daily dose, administration method, duration of administration, and reason for use should be recorded in the case report form.

**1) Leukopenia (Neutropenia):**

Leukopenia typically develops 1-2 weeks after the start of administration, and continued administration can lead to severe infections. In cases of severe leukopenia (neutropenia), careful observation is required. If accompanied by fever, investigate for infection foci and administer antibiotics. Concomitant use of G-CSF is permitted within insurance coverage, but GEM and TS-1 administration must always be suspended (no simultaneous co-administration). Prophylactic administration is not performed.

**Examples of G-CSF Use (Insurance Coverage):**

- **Initiation Timing:**
  - Neutrophil count < 1,000/mm³ with fever (generally ≥ 38°C).
  - When a neutrophil count < 500/mm³ is observed.
  - If, in the previous course, a neutrophil count < 1,000/mm³ with fever (generally ≥ 38°C) or a neutrophil count < 500/mm³ was observed, and a neutrophil count < 1,000/mm³ is observed after the same chemotherapy regimen.
- **Dosage:**
  - Filgrastim (Gran): 50 µg/m² once daily subcutaneously, or 100 µg/m² once daily intravenously.
  - Nartograstim (Neu-up): 1 µg/kg once daily subcutaneously, or 2 µg/kg intravenously.
  - Lenograstim (Neutrogin): 2 µg/kg once daily subcutaneously, or 5 µg/kg once daily intravenously.
- **Discontinuation Timing:**
  - Discontinue administration when the neutrophil count reaches > 5,000/mm³ after passing the nadir.
  - If the neutrophil count recovers to > 2,000/mm³, there are no signs suggestive of infection, and patient safety can be ensured based on the response to the drug, consider discontinuing or reducing the dose of the drug.

**2) Liver Dysfunction:**

Basic bed rest and administration of hepatoprotective agents are recommended. In cases of fulminant progression, there is no particularly effective treatment, but for similar drugs, steroid therapy, G.I. therapy, plasma exchange, and special amino acid infusions have been performed.

**3) Severe Renal Dysfunction:**

In cases of renal failure, carefully manage fluid balance, correct electrolytes, and perform renal dialysis if necessary, while awaiting recovery of urine output. After urine output recovers, a diuretic phase often occurs, so exercise extra caution.

**4) Severe Enteritis/Dehydration:**

In cases accompanied by dehydration, administer sufficient intravenous fluids and antidiarrheal agents such as opium tincture or codeine phosphate. For severe diarrhea, consider administering loperamide. The onset of diarrhea is often accompanied by leukopenia, and severe diarrhea can trigger severe infections, so early intervention is necessary.

**5) Nausea/Vomiting:**

For nausea and vomiting, consider administering antiemetics. For anticipatory nausea and vomiting, administer minor tranquilizers such as diazepam.

**6) Stomatitis:**

For stomatitis, administer allopurinol gargle or steroids.

**7) Infection:**

For infections, administer antibiotics with an appropriate antibacterial spectrum against the causative pathogen.

**8. Observations, Examinations, and Evaluations at Each Time Point**

The principal investigator or sub-investigator shall collect data according to the following observation, examination, and evaluation schedule.

**8.1. Observation, Examination, and Evaluation Schedule**

**8.1.1. At Registration**

Based on the results of the following examinations performed within one month prior to registration, eligibility criteria and exclusion criteria will be determined, and subjects deemed eligible will be registered. It is permissible to use the results of examinations performed as part of routine clinical practice before obtaining consent.

- Physical examination (ECOG PS, etc.)
- Blood tests
- CT scan

**8.1.2. Before GEM Administration**

The following will be performed within 24 hours prior to GEM administration to determine the initiation or continuation of chemotherapy.

- Physical examination (ECOG PS, etc.)
- Blood tests (excluding tumor markers)

**8.1.3. From End of Preoperative Treatment to Immediately Before Surgery**

The following examinations and evaluations will be performed to assess suitability for surgery (refer to Section 7.3).

- Physical examination (ECOG PS, etc.)
- Blood tests
- CT scan

**8.1.4. Post-Surgery**

The following evaluations will be performed.

- Surgical record
- Pathological findings

**8.1.5. Post-Surgery Follow-up (Every 3 Months)**

The following examinations and evaluations will be performed.

- Physical examination
- Blood tests
- CT scan
- Follow-up survey

**8.2. Study Calendar**

|  | **At Registration (within 1 month prior to registration)** | **Before GEM Administration (within 24 hours prior to administration)** | **End of Preoperative Treatment ~ Immediately Before Surgery** | **Post-Surgery** | **Post-Surgery Follow-up (Every 3 months)c** |
| --- | --- | --- | --- | --- | --- |
| **Allowable Range** |  | Day before administration |  |  | ±1 month |
| **Subject Background** | ◎ |  |  |  |  |
| **Physical Examination (ECOG PS, etc.)** | ◎ | ◎ | ◎ |  | ◎ |
| **Chemotherapy/Radiotherapy** |  |  |  |  |  |
| **Blood Tests (excluding tumor markers)** | ◎ | ◎ | ◎ |  | ◎ |
| **Blood Tests (tumor markers)** | ◎ |  | ◎ |  | ◎ |
| **CT Scan** | ◎ |  | ◎ |  | ◎ |
| **Surgical Record** |  |  |  | ◎ |  |
| **Pathological Findings** |  |  |  | ◎ |  |
| **Follow-up Survey** |  |  |  |  | ◎ |
| **Adverse Events** |  |  |  |  |  |

◎: Mandatory item a: Examination is mandatory, but only adverse events observed should be recorded in the case report form. b: If multiple examinations are performed during this period, the data closest to the surgery date should be recorded in the case report form. c: Evaluation will be performed every 3 months for 3 years from the date of surgery. Thereafter, evaluation will be performed at intervals consistent with routine clinical practice.

**9. Procedures for Observation, Examination, and Evaluation**

**9.1. Obtaining Consent**

The principal investigator or sub-investigator shall obtain consent from the research subjects according to the procedures described in Section 15.4.1 before performing the examinations required for this study.

**9.2. Registration and Allocation**

**9.2.1. Registration and Allocation Procedures**

The principal investigator or sub-investigator shall register research subjects according to the following procedures:

(1) The principal investigator or sub-investigator shall enter the necessary information for patients who have given consent into the web registration system and register them. Regarding input into the web registration system, a research collaborator may act on behalf of the principal investigator or sub-investigator under their instruction.

(2) The web registration system will determine the patient's eligibility. If eligible, the treatment group for that research subject will be determined. For allocation, stratified randomization will be applied, using pre-treatment CA19-9 values (<370, ≥370 U/mL) as a stratification factor.

(3) The principal investigator or sub-investigator shall confirm the registration decision result and treatment group on the web registration system.

(4) The principal investigator or sub-investigator shall initiate the assigned treatment for the research subject within 21 days after full registration.

**9.2.2. Creation and Storage of Allocation Table**

The allocation manager shall create the allocation procedure and manage the allocation information of the research subjects. Allocation information shall be stored in a secure location, inaccessible to all parties, including research subjects, principal investigators, and sub-investigators.

**9.3. Subject Background**

The following items will be confirmed based on physical examination, CT scan results, etc.

- Sex
- Age
- Diagnosis method
- Histological classification
- TNM classification (UICC 8th edition)
- Main primary site, size of main primary site
- Height, weight, body surface area (calculated by DuBois formula from height and weight)
- Presence or absence of obstructive jaundice
- Presence or absence of comorbidities other than the above
- Presence or absence of biliary drainage

**9.4. ECOG-PS**

ECOG-PS will be evaluated based on physical examination results.

**9.5. Chemotherapy**

The following items will be confirmed.

- Administration date
- Dose

**9.6. Radiation Therapy**

The following items will be confirmed.

- Radiation irradiation date
- Total irradiation dose per week

**9.7. Surgical Record**

The following items will be confirmed.

- Tumor diameter
- Surgery date
- Surgical procedure
- Concomitant resection site
- Number of dissected lymph nodes
- Blood loss
- Operation time
- Presence or absence of curative resection
- Presence and content of postoperative complications of Clavien-Dindo Grade 3 or higher
- Presence or absence of reoperation, date of reoperation

**9.8. Pathological Findings**

- Tumor diameter
- pTNM classification (UICC 8th edition)
- Number of metastatic positive lymph nodes
- Histological residual tumor grade
- Histological effect (Evans classification)

**Evans Classification [10]**

- **Grade I:** Characteristic cytologic changes of malignancy are present, but little (< 10%) or no tumor cells are destroyed.
- **Grade IIa:** Destruction of 10%-50% of tumor cells.
- **Grade IIb:** Destruction of 51%-90% of tumor cells.
- **Grade III:** Few (< 10%) viable-appearing tumor cells are present.
- **Grade IV:** No viable tumor cells are present. It is desirable that the search and determination be made, in principle, on the largest cross-section passing through the center of the lesion.

**9.9. Blood Tests**

The following items will be measured according to the standard procedures of each participating medical institution.

- **Tumor Markers:** CA19-9, CEA, DUPAN II
- **Others:** Hemoglobin, white blood cells, neutrophil count, platelet count, total bilirubin, albumin, AST, ALT, ALP, blood urea nitrogen, creatinine

**9.10. Post-Treatment**

The following items will be confirmed.

- Presence or absence of post-treatment
- Type of post-treatment
- Date of decision to implement post-treatment
- Name of drug treatment
- Surgical procedure
- Total irradiation dose
- Start date of post-treatment, end date of post-treatment

**9.11. Follow-up Survey**

The following items will be confirmed based on physical examination, tumor markers, CT scan results, etc. If a research subject does not attend a scheduled visit, their survival status will be confirmed by phone or other means whenever possible.

- Life or death
- Date of death or most recent confirmed survival date
- Cause of death
- Presence or absence of progression
- Site
- Date of progression determination or most recent date of no progression determination
- Reason for progression determination

**9.12. Criteria for Discontinuation and Completion for Each Research Subject**

**9.12.1. Discontinuation of Protocol Treatment for Each Research Subject**

The principal investigator or sub-investigator shall discontinue protocol treatment for a research subject if any of the following are found to apply after the subject's registration. Even after discontinuation of protocol treatment, follow-up will continue for all cases until 3 years after the registration date of the last registered case, unless Section 9.12.2 applies.

(1) If the research subject requests discontinuation of protocol treatment.

(2) If disease progression is confirmed.

(3) If the physician determines that continuation of treatment is difficult due to the onset or exacerbation of complications.

(4) If the physician determines that continuation of treatment is difficult due to adverse events.

(5) If the research subject transfers to another hospital.

(6) If the research subject dies.

(7) If chemotherapy is postponed for more than 3 weeks due to adverse events.

(8) If radiation therapy is postponed for more than 2 weeks due to adverse events (GS-RT group only).

(9) If it is found that the case was ineligible after registration.

(10) Other cases where the principal investigator or sub-investigator determines that continuation of protocol treatment is not possible.

**9.12.2. Discontinuation of the Study for Each Research Subject**

The principal investigator or sub-investigator shall discontinue the study for a research subject if any of the following are found to apply after the subject's registration.

(1) If the research subject requests discontinuation of the study.

(2) If it is found that the case was ineligible.

(3) If it is found that future necessary observations and examinations are impossible due to the research subject's circumstances, such as relocation.

(4) Other cases where the principal investigator or sub-investigator determines that the study should be discontinued.

**9.12.3. Procedure for Discontinuation for Each Research Subject**

If the principal investigator or sub-investigator finds that a discontinuation criterion applies, they shall immediately explain this to the research subject and take measures such as alternative treatment if necessary. The principal investigator or sub-investigator shall perform the observations, examinations, and evaluations planned in this research protocol to the extent possible and record the results in the case report form along with the reason for discontinuation.

**9.12.4. Completion for Each Research Subject**

The completion of all planned observations, examinations, and evaluations described in this research protocol shall constitute the completion for that research subject.

**10. Adverse Events**

**10.1. Definition of Adverse Event**

An adverse event is any unfavorable or unintended sign (including abnormal laboratory findings), symptom, or disease occurring in a research participant, regardless of its causal relationship to the study. If a condition present before the start of the study worsens after administration, it will also be treated as an adverse event. In this study, information on adverse events of Grade 3 or higher according to CTCAE v5.0 will be recorded in the case report form.

**10.2. Adverse Event Collection Period**

Information on adverse events occurring from the start date of protocol treatment until the date of surgery will be recorded in the case report form.

**10.3. Adverse Event Ascertainment**

**10.3.1. Confirmation of Subjective Symptoms and Objective Findings**

The principal investigator or sub-investigator will confirm the presence or absence of subjective symptoms from the research participant, in addition to objective findings. In the case of outpatient visits, subjective symptoms and objective findings that occurred outside of the visit will also be elicited from the research participant.

**10.4. Adverse Event Evaluation**

The principal investigator or sub-investigator will record the following in the case report form for adverse events observed during the collection period. If multiple adverse events are observed, each event will be recorded separately.

(1) Adverse event term

(2) Date of onset

(3) Severity

(4) Causal relationship to the study

**10.4.1. Adverse Event Term**

Each event will be described by its diagnosis. Signs (including abnormal laboratory findings, abnormal electrocardiogram findings) and symptoms accompanying that diagnosis will not be listed as separate adverse events. If the diagnosis is unclear, the relevant sign or symptom will be described as an adverse event as appropriate.

**10.4.2. Date of Onset**

The date of onset of an adverse event will be determined according to the following criteria:

**Adverse Event Onset Date**

- **For signs, symptoms, or diseases (diagnosed conditions):**
  - Record the date when the research participant, principal investigator, or sub-investigator first noticed the signs or symptoms of the adverse event.
- **For asymptomatic diseases:**
  - Record the date when diagnostic tests were performed and the diagnosis was confirmed.
  - Even if old findings are observed from laboratory results, or if the approximate time of onset can be estimated, record the date when the diagnosis was confirmed.
- **For exacerbation of pre-existing conditions:**
  - Record the date when the research participant, principal investigator, or sub-investigator first noticed the exacerbation of the disease or symptoms.
- **For abnormalities found in tests after the start of the study drug/product:**
  - Record the date of the test when a clinically significant abnormal laboratory value was observed.
- **For abnormalities observed in tests at the start of the study drug/product, which subsequently worsened in later tests:**
  - Record the date of the test when a clear increase, decrease, or change was observed based on medical judgment from the trend of laboratory values.

**10.4.3. Severity**

The severity of adverse events will be evaluated according to CTCAE v5.0 Japanese translation JCOG/JSCO version.

**10.4.4. Causal Relationship to the Study**

The causal relationship between the conduct of this study and an adverse event will be classified as follows. If a causal relationship is determined to be "unrelated," the reason for this determination will be recorded in the case report form.

- **Related:** There is a clear temporal correlation (including the course after discontinuation of application). Or, other factors such as the underlying disease, co-morbidities, concomitant medications/therapies are also suspected, but the adverse event could also be attributed to the application of the investigational product(s) in this study.
- **Unrelated:** There is no clear temporal correlation with the application of the investigational product(s) in this study. Or, the adverse event is sufficiently attributable to other factors such as the underlying disease, co-morbidities, concomitant medications/therapies.

**10.5. Actions in Case of Adverse Event Occurrence**

**10.5.1. Management of Research Participants** Upon the occurrence of an adverse event, the principal investigator and sub-investigators will provide appropriate emergency treatment, ensure the safety of the research participant, and, if necessary, arrange for diagnosis and treatment by a specialist physician to resolve the event and investigate its cause.

**10.5.2. Follow-up of Adverse Events** Adverse events that occur during the study period will be followed up as long as possible until they resolve or are deemed clinically unnecessary. The method of follow-up, such as hospitalization or outpatient visits, frequency of outpatient visits, and laboratory tests, will be determined by the principal investigator or sub-investigator based on the type and severity of the adverse event.

**10.6. Expected Adverse Events in This Study**

**10.6.1. GM** The following adverse reactions are listed in the package insert for GM (Gemzar) (revised December 2024 (2nd edition)). The principal investigator or sub-investigator will confirm the latest package insert before administration.

**(1) Serious Adverse Reactions**

1. **Myelosuppression:** Leukopenia (72.6%, with a decrease to <2000/μL in 17.5%), neutropenia (69.2%, with a decrease to <1000/μL in 32.1%), thrombocytopenia (41.4%, with a decrease to <50,000/μL in 4.2%), anemia [hemoglobin decrease (66.5%, with a decrease to <8.0g/dL in 13.1%), erythrocyte decrease (52.6%)] etc. may occur. Fatal cases due to sepsis, thought to be caused by severe leukopenia, have been reported.
2. **Interstitial Pneumonia (1.0%):** If interstitial pneumonia or acute exacerbation is suspected, immediately discontinue treatment with this drug and provide appropriate treatment such as steroid therapy. Fatal cases thought to be caused by interstitial pneumonia have been reported.
3. **Anaphylaxis (0.2%):** Symptoms such as dyspnea, hypotension, and rash may occur.
4. **Myocardial Infarction (0.2%)**
5. **Congestive Heart Failure (frequency unknown)**
6. **Pulmonary Edema (frequency unknown)**
7. **Bronchospasm (frequency unknown)**
8. **Adult Respiratory Distress Syndrome (ARDS) (frequency unknown)**
9. **Renal Failure (frequency unknown)**
10. **Hemolytic Uremic Syndrome (0.2%):** If signs of microangiopathic hemolytic anemia, such as rapid hemoglobin decrease accompanied by thrombocytopenia, elevated bilirubin, elevated creatinine, elevated BUN, and elevated LDH, are observed, discontinue administration. Renal failure may be irreversible even after discontinuation, and dialysis may be required.
11. **Severe Skin Disorders (frequency unknown):** Severe skin disorders such as Toxic Epidermal Necrolysis (TEN), Stevens-Johnson Syndrome, erythema, blisters, and desquamation may occur.
12. **Hepatic Dysfunction, Jaundice (frequency unknown):** Serious hepatic dysfunction such as elevated AST, ALT, Al-P, and jaundice may occur.
13. **Leukoencephalopathy (including Reversible Posterior Leukoencephalopathy Syndrome) (frequency unknown):** If symptoms such as hypertension, convulsions, headache, visual abnormalities, and impaired consciousness are observed, discontinue administration and provide appropriate treatment.

**(2) Other Adverse Reactions**

| **System/Organ Class** | **≥10%** | **1% to <10%** | **<1%** | **Frequency Unknown** |
| --- | --- | --- | --- | --- |
| **Cardiovascular** |  | Tachycardia, Blood pressure elevation | Hypotension, Angina pectoris, Palpitations, Ventricular extrasystoles, Paroxysmal supraventricular tachycardia, ECG abnormalities (ST elevation) |  |
| **Respiratory** |  | Dyspnea, Hypercapnia (Note 1), Hypoxemia, Cough | PIE (Pulmonary Infiltrates with Eosinophilia) syndrome, Wheezing, Sputum, Shortness of breath |  |
| **Renal** | Decreased total protein, Electrolyte abnormalities, Decreased albumin | Elevated BUN, Proteinuria, Hematuria, Elevated creatinine | Oliguria |  |
| **Gastrointestinal** | Anorexia, Nausea/Vomiting | Diarrhea, Constipation, Stomatitis, Gastric discomfort | Gingivitis |  |
| **Hepatic** | Elevated AST, Elevated ALT, Elevated LDH, Elevated Al-P | Elevated bilirubin, Decreased A/G ratio, Elevated γ-GTP, Urobilinuria |  |  |
| **Psychiatric/Neurological** |  | Headache, Dizziness, Insomnia, Paresthesia (Note 2) | Lethargy, Numbness |  |
| **Skin** | Rash | Alopecia (Note 2), Pruritus | Urticaria |  |
| **Injection Site** |  | Injection site reactions (Phlebitis, Pain, Erythema) |  |  |
| **Vascular** |  | Peripheral vasculitis (Note 2) | Peripheral gangrene |  |
| **Other** | Fatigue, Fever, Thrombocytosis | Weight loss, Glycosuria positive, Eosinophilia, Arthralgia (Note 2), Chills, Taste alteration (Note 2), Epistaxis, Malaise (Note 2), Edema, Elevated CRP, Weight gain, Pain (Note 2), Hot flashes, Chest discomfort | Fundus hemorrhage, Decreased body temperature, Tinnitus, Eye discharge, Asthenia, Facial edema | Influenza-like symptoms (malaise, asthenia, fever, headache, chills, myalgia, sweating, rhinitis, etc.), Radiation recall reaction |

Note 1) Frequency in 11 cases from a clinical trial for pancreatic cancer. Note 2) Observed in over 30% of cases in domestic clinical trials of this drug in combination with paclitaxel.

**10.6.2. S-1**

The following adverse reactions are listed in the package insert for S-1 (TS-1) (revised January 2025 (4th edition)). The principal investigator or sub-investigator will confirm the latest package insert before administration.

**(1) Serious Adverse Reactions**

1. **Myelosuppression, Hemolytic Anemia:** Severe myelosuppression such as pancytopenia, agranulocytosis (symptoms: fever, sore throat, malaise, etc.) (both frequency unknown), leukopenia (46.7%), anemia (frequency unknown), thrombocytopenia (15.7%), and hemolytic anemia (frequency unknown) may occur.
2. **Disseminated Intravascular Coagulation (DIC) (0.4%):** If abnormalities are observed in blood tests such as platelet count, serum FDP levels, and plasma fibrinogen concentration, discontinue administration and provide appropriate treatment.
3. **Severe Hepatic Dysfunction such as Fulminant Hepatitis:** Severe hepatic dysfunction such as fulminant hepatitis (including those due to reactivation of hepatitis B virus) (frequency unknown) may occur.
4. **Dehydration:** Severe diarrhea may occur, leading to dehydration (frequency unknown). If such symptoms appear, discontinue administration and provide appropriate treatment such as fluid replacement.
5. **Severe Enteritis (0.5%):** Hemorrhagic enteritis, ischemic enteritis, necrotic enteritis, etc. may occur. If symptoms such as severe abdominal pain or diarrhea appear, discontinue administration and provide appropriate treatment.
6. **Interstitial Pneumonia:** Interstitial pneumonia (0.3%) (Note 1) (initial symptoms: cough, shortness of breath, dyspnea, fever, etc.) may occur. If abnormalities are observed, discontinue administration, perform examinations such as chest X-ray, and provide appropriate treatment such as steroid therapy.
7. **Myocardial Infarction, Angina Pectoris, Arrhythmia, Heart Failure:** Myocardial infarction, angina pectoris, arrhythmia (including ventricular tachycardia, etc.), and heart failure (all frequency unknown) may occur. If chest pain, syncope, palpitations, ECG abnormalities, shortness of breath, etc. are observed, discontinue administration and provide appropriate treatment.
8. **Severe Stomatitis (frequency unknown), Gastrointestinal Ulcer (0.5%), Gastrointestinal Hemorrhage (0.3%), Gastrointestinal Perforation (frequency unknown):** If abnormalities are observed, discontinue administration, perform necessary examinations such as abdominal X-ray, and provide appropriate treatment.
9. **Acute Renal Failure, Nephrotic Syndrome (frequency unknown)**
10. **Toxic Epidermal Necrolysis (TEN), Stevens-Johnson Syndrome (both frequency unknown)**
11. **Neuropsychiatric Disorders including Leukoencephalopathy:** Leukoencephalopathy (with main symptoms such as impaired consciousness, cerebellar ataxia, dementia-like symptoms), impaired consciousness, disorientation, somnolence, memory impairment, extrapyramidal symptoms, speech disorder, quadriplegia, gait disturbance, urinary incontinence, sensory impairment (all frequency unknown), etc. may occur.
12. **Acute Pancreatitis (frequency unknown):** If abdominal pain, elevated serum amylase levels, etc. appear, discontinue administration and provide appropriate treatment.
13. **Rhabdomyolysis:** Rhabdomyolysis (frequency unknown) characterized by myalgia, weakness, elevated CK, and elevated blood and urine myoglobin may occur. Also, be aware of the onset of acute renal failure due to rhabdomyolysis.
14. **Anosmia:** Olfactory disorder (0.1%) may occur, leading to anosmia (frequency unknown).
15. **Lacrimal Duct Obstruction (frequency unknown):** Cases requiring surgical intervention have been reported. If symptoms such as epiphora appear, provide appropriate treatment including ophthalmological examination.
16. **Liver Cirrhosis (prolonged prothrombin time, decreased albumin, decreased cholinesterase, etc.) (frequency unknown)**

**(2) Other Adverse Reactions**

| **System/Organ Class** | **≥5%** | **0.1% to <5%** | **Frequency Unknown** |
| --- | --- | --- | --- |
| **Blood** | Leukopenia, Neutropenia, Thrombocytopenia, Erythrocytopenia, Hemoglobin decrease, Hematocrit decrease, Lymphocytopenia | Bleeding tendency (subcutaneous hemorrhage, epistaxis, coagulation factor abnormalities), Eosinophilia, Leukocytosis |  |
| **Hepatic** | Elevated AST, Elevated ALT, Elevated bilirubin, Elevated Al-P | Jaundice, Urine urobilinogen positive |  |
| **Renal** |  | Elevated BUN, Elevated creatinine, Proteinuria, Hematuria |  |
| **Gastrointestinal** | Anorexia, Nausea/Vomiting, Diarrhea, Stomatitis, Taste alteration | Intestinal obstruction, Ileus, Abdominal pain, Abdominal distension, Epigastric pain, Gastritis, Borborygmi, White stool, Constipation, Cheilitis, Labial inflammation, Glossitis, Dry mouth |  |
| **Skin** | Pigmentation | Erythema, Desquamation, Flushing, Blisters, Hand-foot syndrome (Note 2), Skin ulcer, Dermatitis, Alopecia, Nail abnormalities, Paronychia, Herpes simplex, Dry/rough skin | Photosensitivity, DLE-like rash |
| **Hypersensitivity** | Rash | Pruritus |  |
| **Psychiatric/Neurological** | General malaise | Numbness, Headache, Heaviness in head, Dizziness | Dizziness, Peripheral neuropathy |
| **Cardiovascular** |  | Hypotension, Hypertension, ECG abnormalities, Raynaud's phenomenon | Palpitations |
| **Ocular** |  | Epiphora (Note 3), Conjunctivitis, Keratitis, Corneal erosion, Eye pain, Decreased visual acuity, Dry eyes | Corneal ulcer, Corneal opacity, Limbal stem cell deficiency |
| **Other** | Elevated LDH, Decreased total protein, Decreased albumin | Fever, Generalized hot sensation, Rhinitis, Pharyngitis, Sputum, Glycosuria, Elevated blood glucose, Edema, Myalgia, Elevated CK, Arthralgia, Electrolyte abnormalities (elevated serum sodium, decreased serum sodium, elevated serum potassium, decreased serum potassium, elevated serum calcium, decreased serum calcium, elevated serum chloride, decreased serum chloride), Weight loss | Elevated serum amylase |

Frequencies are calculated from clinical trials of monotherapy up to approval. Note 1) In a post-marketing surveillance study for non-small cell lung cancer, interstitial pneumonia was 0.7% (11/1669 cases), and lung disorders such as radiation pneumonitis, dyspnea, and respiratory failure were 0.7% (12/1669 cases). Note 2) In previously treated breast cancer, the incidence of hand-foot syndrome was high at 21.8%. Note 3) In a post-marketing clinical trial for unresectable or recurrent gastric cancer treated with TS-1 monotherapy, the incidence of epiphora was high at 16.0%.

**10.6.3. Radiation Therapy**

The following are generally known adverse reactions associated with radiation therapy for pancreatic cancer, and these adverse reactions may rarely appear several years after treatment: Myelosuppression, nausea/vomiting, decreased appetite, gastrointestinal (stomach, duodenum, small intestine, large intestine, etc.) ulcers/hemorrhage/stricture, renal/hepatic dysfunction, skin disorders, etc.

**11. Diseases and Other Conditions**

**11.1. Definition of Diseases and Other Conditions**

"Diseases and other conditions" refers to any disease, disability, or death, or infection suspected to be caused by the conduct of this study, as well as abnormal clinical laboratory values and various symptoms.

**11.2. Reporting Procedures for Diseases and Other Conditions to the Certified Clinical Research Review Board, etc.**

If a disease or other condition occurs, the attending physician will promptly inform the principal investigator. The principal investigator will report the first report, to the extent known at that time, to the overall administrator and the administrator of the implementing medical institution within the periods specified in the following items. When the overall administrator becomes aware of the matters listed below, they will report them to the Certified Clinical Research Review Board within the specified period for each, and notify other principal investigators of the same. The notified principal investigators will promptly report the content to the administrator of their implementing medical institution.

1. Among the following, those suspected to be caused by the conduct of this study and are unexpected: **7 days** 
   a. Death
   b. Diseases or other conditions that may lead to death
2. The following matters: **15 days** 
   a. Among the following, those suspected to be caused by the conduct of this study (excluding those listed in 1):
   (1) Death
   (2) Diseases or other conditions that may lead to death
   b. Among the following, those suspected to be caused by the conduct of this study and are unexpected:
   (1) Diseases or other conditions requiring hospitalization or extension of hospitalization for treatment
   (2) Disability
   (3) Diseases or other conditions that may lead to disability
   (4) Diseases or other conditions that are serious in a manner equivalent to (1) to (3) and death and diseases or other conditions that may lead to death
   (5) Congenital diseases or abnormalities in future generations
3. Among the following, those suspected to be caused by the conduct of this study (excluding those listed in 2.b and those that occurred in specific clinical research where a Safety and Efficacy Evaluation Committee was established): **30 days** 
   a. Diseases or other conditions requiring hospitalization or extension of hospitalization for treatment
   b. Disability
   c. Diseases or other conditions that may lead to disability
   d. Diseases or other conditions that are serious in a manner equivalent to a to c and death and diseases or other conditions that may lead to death
   e. Congenital diseases or abnormalities in future generations
4. Those suspected to be caused by the conduct of this study (other than 1-3 above): When submitting the periodic report to the Certified Clinical Research Review Board as per Article 17, Paragraph 1 of the Act.

**● Diseases and Other Conditions Subject to Reporting to the Certified Clinical Research Review Board**

| **Disease/Condition** | **Unexpected** | **Expected** |
| --- | --- | --- |
| a. Death | 7 days | 15 days |
| b. Diseases or other conditions that may lead to death | 7 days | 15 days |
| c. Diseases or other conditions requiring hospitalization or extension of hospitalization for treatment | 15 days | 30 days |
| d. Disability | 15 days | 30 days |
| e. Diseases or other conditions that may lead to disability | 15 days | 30 days |
| f. Diseases or other conditions that are serious in a manner equivalent to c to e and death and diseases or other conditions that may lead to death | 15 days | 30 days |
| g. Congenital diseases or abnormalities in future generations | 15 days | 30 days |
| h. Other diseases or conditions | Periodic Report | Periodic Report |

If there is a change in the reported content of a disease or other condition, such as a change in outcome, the principal investigator or sub-investigator will notify the administrator of the implementing medical institution and the overall administrator, and the overall administrator will report it to the Certified Clinical Research Review Board.

**11.3. Reporting Procedures for Serious Diseases and Other Conditions to the Minister of Health, Labour and Welfare**

When the overall administrator becomes aware of the matters in 11.2, items 1 and 2.b, they will report them to the Minister of Health, Labour and Welfare via the jRCT disease/condition reporting screen within the period specified in the table in that section.

**12. Data Management**

**12.1. Data Management Plan**

The data manager will perform database entry, data cleaning, query generation, and dataset creation for statistical analysis based on the case report forms. For missing values, blank values, abnormal values suspected of being errors, etc., the data manager will inquire with the principal investigator or sub-investigator. The data manager will confirm the response or data correction content from the principal investigator or sub-investigator.

**12.2. Central Monitoring**

Central monitoring will be conducted according to the monitoring procedures.

**12.3. Case Report Forms**

The principal investigator or sub-investigator will create case report forms for all research participants from whom informed consent has been obtained. An EDC (Electronic Data Capture) system will be used for case report forms. The data manager will provide training to the principal investigator and sub-investigators on the use of the EDC system. The principal investigator or sub-investigator will directly record data into the EDC system when creating case report forms. Changes or corrections to case report forms will be recorded as an audit trail, documenting information before and after the change or correction, the person who made the change or correction, the date of the change or correction, and the reason for it. The principal investigator will confirm that the case report forms are accurately and completely prepared and will sign (electronically) the relevant sections of the case report forms. The principal investigator bears full responsibility for the accuracy and reliability of all data recorded in the case report forms.

**12.4. Data Directly Recorded in Case Report Forms**

The following data, when directly recorded in the case report form, constitute source data:

- Causal relationship between the study and adverse events
- Comments from the principal investigator or sub-investigator
- Reasons for discontinuation of protocol treatment for each research participant
- Reasons for discontinuation for each research participant
- Other data not recorded in medical records

**13. Statistical Matters**

**13.1. Definition of Analysis Population**

In this study, the analysis population will consist of research participants from the total enrolled cases who received at least one dose of chemotherapy or chemoradiotherapy and underwent at least one evaluation.

**13.2. Data Handling**

Data handling will be as follows. The overall administrator and the head of statistical analysis will discuss and decide on the analytical handling of all research participant data before data lock.

**13.2.1. Handling of Measurements Outside Prescribed Tolerances**

For data deviating from the prescribed tolerance range for evaluation items, the overall administrator and the head of statistical analysis will discuss and decide on their acceptance or rejection. If multiple data points exist within the relevant range, the absolute difference in days from the prescribed evaluation date will be calculated, and the data point with the smallest absolute difference will be adopted as the data for that evaluation period. If the absolute differences are the same, individual consideration (per evaluation item) will be given. Data from the post-surgery follow-up period are excluded from this handling.

**13.2.2. Handling of Outliers**

The handling of outliers will be discussed and decided before analysis. Depending on the variable, consideration will be given to appropriate variable transformations or the application of statistical methods that are not significantly affected by outliers.

**13.2.3. Handling of Missing Values**

Missing data will not be imputed.

**13.2.4. Handling of Lost to Follow Up**

Patients lost to follow-up will be treated as censored at the date of last confirmed survival or non-progression for time-to-event analyses. For secondary endpoints other than time-to-event outcomes, handling of lost to follow-up cases will depend on the timing of discontinuation. For resection rate, R0 resection rate, and histological response, patients lost to follow-up before surgery will be excluded from the analysis, those lost during surgery will be considered as non-resected cases, and those lost after surgery will be included in the analysis. For grade ≥3 adverse events, all patients, including those lost to follow-up, will be included in the analysis.

**13.3. Analysis Methods**

The treatment groups below will be determined for each research participant based on the treatment actually administered. Unless otherwise specified for the analysis target, the "analysis population" defined in 13.1 will be used.

P-value < 0.05 will be considered statistically significant. Multiplicity across secondary endpoints will not be adjusted.

All statistical analyses will be conducted using JMP (latest version at the timing of analysis).

**13.3.1. Baseline Characteristics of Research Participants**

- **Analysis Item:** Baseline characteristics of research participants
- **Analysis Method:** Demographic and other characteristics will be summarized using descriptive statistics by treatment group. For continuous variables, mean, standard deviation, minimum, median, and maximum will be calculated. For categorical variables, frequency and percentage will be calculated.

**13.3.2. Primary Endpoint**

- **Analysis Item:** Overall Survival (OS)
- **Analysis Method:** Overall survival is defined as the period from the date of randomization to the date of death from any cause. For surviving cases where death was not observed during the observation period, data will be censored at the last confirmed survival date. As the primary analysis, Kaplan-Meier curves will be generated for each treatment group. Based on the Kaplan-Meier survival function, the 3-year survival rate and median survival time will be estimated. For this, the 95% confidence interval for the time-point survival rate will use Greenwood's formula and the complementary log-log transformation, and the 95% confidence interval for the median survival time will use the Brookmeyer-Crowley method. A log-rank test will be performed to compare survival curves between groups. Furthermore, an estimation of the hazard ratio for the chemoradiotherapy group versus the chemotherapy group using a Cox proportional hazards model, adjusted for pre-treatment CA19-9 values (<370, ≥370 U/mL), and a stratified log-rank test will be conducted. Proportional hazards assumptions will be tested using Schoenfeld residuals. As a supplementary analysis, the same analysis as above will be performed for the "analysis population" where treatment groups are determined based on the assigned treatment. As a subgroup analysis, for each subgroup defined by baseline factors (sex, age, etc.), ECOG-PS, surgical records (tumor size, surgical procedure, etc.), the adjusted hazard ratio will be estimated and the 95% confidence interval calculated, similar to the primary analysis. A forest plot will also be created.

**13.3.3. Secondary Endpoints**

- **Analysis Item:** Resection Rate
- **Analysis Method:** Resection rate is defined as the proportion of resected cases in the analysis population. Point estimates and 95% confidence intervals for the resection rate will be calculated for each treatment group. Additionally, the point estimate of the odds ratio for the resection rate, 95% confidence interval, and two-sided p-value will be calculated using a logistic regression model adjusted for pre-treatment CA19-9 values (<370, ≥370 U/mL).
- **Analysis Item:** R0 Resection Rate
- **Analysis Population:** Resected cases within the analysis population
- **Analysis Method:** R0 resection rate is defined as the proportion of R0 resected cases in the analysis population. Point estimates and 95% confidence intervals for the R0 resection rate will be calculated for each treatment group. Additionally, the point estimate of the odds ratio for the R0 resection rate, 95% confidence interval, and two-sided p-value will be calculated using a logistic regression model adjusted for pre-treatment CA19-9 values (<370, ≥370 U/mL).
- **Analysis Item:** Histological Effect of Preoperative Treatment
- **Analysis Population:** Resected cases within the analysis population
- **Analysis Method:** Point estimates and 95% confidence intervals for the proportion of each Evans classification Grade will be calculated for each treatment group. A chi-square test will be performed for the null hypothesis "the distribution of Evans classification Grades is equal between the chemoradiotherapy group and the chemotherapy group," and a two-sided p-value will be calculated. Furthermore, Evans classification will be divided into I+IIa and IIb+III+IV, and the point estimate of the odds ratio, 95% confidence interval, and two-sided p-value will be calculated using a logistic regression model adjusted for pre-treatment CA19-9 values (<370, ≥370 U/mL).
- **Analysis Item:** Progression-Free Survival (PFS)
- **Analysis Method:** Progression-free survival is defined as the period from the date of randomization to the earlier of the date of confirmed progression or the date of death from any cause. For surviving cases not confirmed to have progressed during the observation period, data will be censored at the last date progression was confirmed absent. Kaplan-Meier curves will be generated for each treatment group. Based on the Kaplan-Meier survival function, the 3-year progression-free rate and median progression-free time will be estimated. For this, the 95% confidence interval for the time-point progression-free rate will use Greenwood's formula and the complementary log-log transformation, and the 95% confidence interval for the median progression-free time will use the Brookmeyer-Crowley method. A log-rank test will be performed to compare survival curves between groups. Furthermore, an estimation of the hazard ratio for the chemoradiotherapy group versus the chemotherapy group using a Cox proportional hazards model, adjusted for pre-treatment CA19-9 values (<370, ≥370 U/mL), and a stratified log-rank test will be conducted.
- **Analysis Item:** Grade 3 or Higher Adverse Events
- **Analysis Method:** Point estimates and 95% confidence intervals for the incidence of Grade 3 or higher adverse events will be calculated for each treatment group. Additionally, the point estimate of the odds ratio, 95% confidence interval, and two-sided p-value will be calculated using a logistic regression model adjusted for pre-treatment CA19-9 values (<370, ≥370 U/mL). The number of occurrences and incidence rate will be tabulated by adverse event and severity for each treatment group. A similar tabulation will be performed for adverse events for which a causal relationship with the study cannot be ruled out.

**13.4. Criteria for Interim Analysis and Early Termination**

No interim analysis will be conducted in this study.

**13.5. Changes to the Statistical Analysis Plan**

If changes or additions to the analysis arise after the start of this study, the overall administrator will consider their validity and impact on the evaluation of this study, consult with the head of statistical analysis, revise the research protocol, and explain the circumstances leading to the change in the analysis plan in the final report of this study.

**14. Quality Control and Quality Assurance**

**14.1. Quality Control Policy**

This study will be conducted in compliance with the Clinical Research Act and its enforcement regulations. Therefore, a quality control policy will be established to meet the requirements of these laws and regulations.

**14.2. Quality Objectives**

We will ensure the reliable creation and storage of documents required by the Clinical Research Act and adhere to provisions regarding the protection of research participants. A dropout rate (including deviations) of 10% will be allowed for the number of research participants whose primary endpoint can be analyzed.

**14.3. Monitoring**

To ensure the reliability of clinical research and the protection of research participants, the overall administrator will designate a monitoring person and establish monitoring procedures to ensure that the clinical research is conducted appropriately, including monitoring the progress of this study and its adherence to laws, regulations, and the research protocol. The monitoring person will conduct monitoring according to the monitoring procedures and create records (monitoring reports) thereof. The principal investigator or the administrator of the implementing medical institution will ensure that the monitoring person has access to the source documents.

**14.4. Response to Inspections by Regulatory Authorities, etc.**

The principal investigator or the administrator of the implementing medical institution will accept requests for inspection from the Certified Clinical Research Review Board or its designated person, and the Minister of Health, Labour and Welfare or their designated person, and will ensure that source documents and other necessary materials are available for viewing.

**14.5. Non-compliance**

**14.5.1. Definition of Non-compliance**

Non-compliance refers to non-adherence to the Clinical Research Act Enforcement Regulations, research protocol, procedures, etc., as well as falsification or fabrication of research data. When managing non-compliance, the following procedures will be followed.

**14.5.2. Non-compliance Management Procedures**

The principal investigator will record all non-compliance identified in this study. When a sub-investigator becomes aware of non-compliance, they will promptly report it to the principal investigator. When the principal investigator becomes aware of non-compliance, they will promptly report it to the overall administrator and the administrator of the implementing medical institution. The overall administrator will promptly inform other principal investigators (excluding the one who reported the non-compliance) about the occurrence of non-compliance and will periodically report the status of non-compliance and subsequent actions to the Certified Clinical Research Review Board.

**14.5.3. Serious Non-compliance**

If particularly serious non-compliance is identified, the overall administrator will promptly seek the opinion of the Certified Clinical Research Review Board. Particularly serious non-compliance refers to anything that affects the human rights or safety of clinical research participants, or the progress or reliability of research results. Examples include non-adherence to selection/exclusion criteria, discontinuation criteria, or prohibited concomitant therapies. It does not include cases where the research protocol was not followed due to unavoidable medical reasons to avoid immediate danger to clinical research participants. In cases exemplified below, regardless of the research content, it is considered to fall under serious non-compliance: ① If informed consent was not obtained. ② If permission from the administrator of the implementing medical institution was not obtained. ③ If the opinion of the Certified Clinical Research Review Board was not sought. ④ If a health hazard occurred to a research participant due to deviation from the research plan. ⑤ If research data was falsified or fabricated. ⑥ Other cases deemed serious non-compliance by the Certified Clinical Research Review Board. The overall administrator will post the materials from seeking the opinion of the Certified Clinical Research Review Board on jRCT.

**15. Ethical Considerations**

**15.1. Regulations to be Observed**

This study will be conducted in accordance with the ethical principles based on the Declaration of Helsinki, the Clinical Research Act, its enforcement regulations, and other related notifications. The principal investigator and sub-investigators will conduct protocol treatment in compliance with this research protocol.

**15.2. Approval by the Certified Clinical Research Review Board and the Administrator of the Implementing Medical Institution**

This study will be implemented after the Certified Clinical Research Review Board reviews and approves its appropriateness, and after approval by the administrator of the implementing medical institution, and after publication on jRCT.

**15.3. Financial Burden on Research Participants in This Study**

Since this study will be conducted within the scope of standard medical care, there will be no increased financial burden on research participants due to participation in the study. No honoraria or other payments will be made to research participants.

**15.4. Informed Consent Document and Consent of Research Participants**

**15.4.1. Consent Acquisition Procedure**

When obtaining consent from research participants, the overall administrator will prepare an informed consent document and obtain approval from the Certified Clinical Research Review Board. The principal investigator or sub-investigator will explain the study to research participants based on the informed consent document approved by the Certified Clinical Research Review Board, provide sufficient time for the research participants to consider, confirm that the research participants fully understand the content of this study, and then request participation in this study. If the research participant consents, their signature will be obtained on the prescribed consent form. The principal investigator or sub-investigator will confirm that the consent form includes the name of the physician who provided the explanation regarding consent, the date of explanation, and the date of consent acquisition. Research participants may withdraw their consent to participate in this study at any time, even after having given consent, at their own free will. If consent is withdrawn, research participants should, if possible, sign the prescribed withdrawal form and submit it to the principal investigator or sub-investigator. A copy of the consent form and withdrawal form will be handed to the research participant, and the original consent form and withdrawal form will be appropriately stored according to the method determined by the implementing medical institution. If the informed consent document is revised with information that may affect the research participant's willingness to participate in the study, for research participants who have already given consent, the principal investigator or sub-investigator will explain the revised informed consent document (approved by the Certified Clinical Research Review Board) and re-obtain consent.

**15.4.2. Matters to be Stated in the Informed Consent Document**

The informed consent document shall include the following:

1. The name of the study and that its implementation has been approved by the administrator of the implementing medical institution and that the implementation plan has been submitted to the Minister of Health, Labour and Welfare.
2. The name or title of the overall administrator, the name and title of the principal investigator, and the name of the implementing medical institution.
3. The purpose and significance of this study.
4. An overview of the investigational product(s).
5. The methods (including the purpose of using samples/information obtained from research participants) and duration of this study.
6. The reason for being selected as a research participant.
7. The burden on research participants and the anticipated risks and benefits.
8. That consent can be withdrawn at any time, even if consent has been given for this study to be implemented or continued.
9. That research participants will not be treated disadvantageously by not consenting to or withdrawing consent for this study to be implemented or continued.
10. The method of disclosing information about this study.
11. That the research protocol and materials related to the methods of this study can be obtained or viewed upon request from research participants, etc., within a scope that does not hinder the protection of personal information of other research participants, etc., and the originality of the study, and the method of obtaining or viewing them.
12. Handling of personal information (including methods to prevent identification of specific individuals).
13. Methods of storage and disposal of samples/information.
14. The status of conflicts of interest related to the research of the research institution, such as funding sources for this study, and conflicts of interest related to the research of researchers, etc., such as personal income.
15. Response to consultations, complaints, and inquiries from research participants, etc., and their relatives.
16. Financial burden or remuneration for research participants, etc.
17. The existence and content of other treatment methods, and matters concerning the anticipated benefits and disadvantages of other treatment methods.
18. The existence and content of compensation for health damage caused by this study.
19. Matters concerning the Certified Clinical Research Review Board that conducts review and opinion services for specific clinical research, and other matters related to the Certified Clinical Research Review Board for the specific clinical research.
20. Other necessary matters concerning the implementation of specific clinical research.

**15.5. Contact Point for Research Participants** Questions from research participants and their relatives will be handled by the principal investigator or sub-investigator.

**15.6. Expected Benefits and Disadvantages for Research Participants**

**15.6.1. Expected Benefits**

Since GS therapy is a treatment performed in standard medical care, benefits equivalent to standard medical care can be obtained. In the GS-RT group, although the chemotherapy administration pattern is slightly different, since GS therapy is performed, benefits almost equivalent to standard medical care can be obtained. This study aims to verify, through a randomized controlled trial, the possibility that combining radiation therapy with GS therapy, as suggested by retrospective comparisons, can enhance the therapeutic effect for resectable pancreatic cancer. If the efficacy and safety of GS-RT therapy for resectable pancreatic cancer are clarified by this study, it can provide a new treatment option for future patients with resectable pancreatic cancer.

**15.6.2. Expected Disadvantages**

The various examinations conducted in this study are within the scope of standard medical care, and there will be no increased burden on research participants due to participation in the study. Since GS therapy is a treatment performed in standard medical care, there are no disadvantages exceeding standard medical care. However, in the GS-RT group, the additional radiation therapy may cause the adverse reactions described in section 10.6. Although a slightly lower dose of S-1 compared to the GS group might potentially weaken the effect of chemotherapy, considering the overall preoperative treatment in the GS-RT group, this difference is considered minor, making such a possibility low.

**15.6.3. Overall Assessment of Benefits and Disadvantages and Measures to Minimize Disadvantages**

For GEM, S-1, and radiation therapy, information on the incidence of adverse reactions and methods for managing them has been accumulated, making it unlikely that disadvantages will outweigh benefits. Furthermore, before participating in this study, research participants will be thoroughly informed, and their intentions will be confirmed.

**15.7. Confidentiality of Research Participants (Protection of Personal Information)**

When handling raw data and consent forms related to the implementation of this study, sufficient consideration will be given to protecting the confidentiality of research participants. Research participants who have given consent will be assigned a research participant identification code. The research participant identification code will consist of numerical symbols, etc., unrelated to information that can identify specific individuals, such as initials or medical record IDs. When creating documents related to this study, such as case report forms, the use of the research participant identification code will prevent the identification of specific individuals. The principal investigator will strictly manage the storage of correspondence tables, etc., to prevent the leakage of personal information such as research participants' names to external parties. When publishing research results, information that can identify research participants will not be included.

**16. Compensation for Health Damage**

In the event that a research participant suffers health damage due to the conduct of this study, the principal investigator, sub-investigators, and the implementing medical institution will provide medical care and other necessary measures to ensure that the research participant can immediately receive appropriate diagnosis, treatment, and necessary care. Costs for treatment will be handled in the same way as standard insurance medical care; no clinical research insurance will be taken out, and no special compensation will be provided.

**17. Suspension or Termination of the Entire Clinical Study**

**17.1. Criteria for Suspension**

The overall administrator will suspend or interrupt the study as necessary if any of the following apply:

(1) If new significant information is obtained that could adversely affect the safety of research participants or the conduct of this study, such as when expected adverse events (diseases, etc.) significantly exceed initial assumptions.

(2) If it is determined to be extremely difficult to achieve the target number of research participants, such as when participant enrollment is significantly slower than planned.

(3) If an opinion is received from the Certified Clinical Research Review Board stating that this study should be suspended.

(4) If other circumstances arise that necessitate the suspension or interruption of this study.

**17.2. Suspension Procedures**

If the overall administrator determines that the suspension criteria are met and decides to suspend the entire study, they will promptly notify the principal investigator. The principal investigator or sub-investigator will explain the study suspension to the research participants, conduct observations, examinations, and evaluations planned in this study to the extent possible, and take medical measures such as alternative treatments as necessary. The overall administrator will submit a suspension notification to the Certified Clinical Research Review Board and report it to the Minister of Health, Labour and Welfare within 10 days from the date of deciding to suspend the entire study.

**17.3. Termination Criteria**

The date on which the overall administrator publishes the summary of the final report by recording it in jRCT will be considered the termination date of this study.

**18. Information Disclosure and Publication of Results**

**18.1. Study Registration**

The overall administrator will register this study in jRCT prior to its implementation. Updates will be made as appropriate in response to changes in the research protocol and study progress.

**18.2. Publication of Research Results**

**18.2.1. Primary Endpoint Report**

The overall administrator will prepare a primary endpoint report within principle one year from the end date of data collection for the primary endpoint, seek the opinion of the Certified Clinical Research Review Board, and notify the principal investigator. The principal investigator will report the content of this notification to the administrator of the implementing medical institution. The overall administrator will publish the report by recording it in jRCT within one month from the date the Certified Clinical Research Review Board expresses its opinion. In this study, the preparation of the primary endpoint report is scheduled to coincide with the preparation of the final report, so the preparation of the final report will be deemed to include the primary endpoint report.

**18.2.2. Final Report**

The overall administrator will prepare a final report and its summary within principle one year from the end date of data collection for all evaluation items. The final report will include at least the following items: (1) Background information of clinical research participants (age, sex, etc.)

(2) Information on the progress status according to the clinical research design (changes in the number of participants, etc.)

(3) Summary of the incidence of diseases and other conditions

(4) Data analysis and results of primary and secondary endpoints

The overall administrator will seek the opinion of the Certified Clinical Research Review Board on the final report and its summary, and notify the principal investigator. The principal investigator will report the content of this notification to the administrator of the implementing medical institution. The overall administrator will publish the summary of the final report, the research protocol, and the statistical analysis plan (if prepared) by recording them in jRCT within one month from the date the Certified Clinical Research Review Board expresses its opinion.

**18.2.3. Publication in Academic Societies, etc.** The results obtained from this study will be promptly presented at academic conferences and published in journals. When publishing, necessary measures will be taken to protect the human rights and interests of research participants and their relatives. Presenters at academic conferences and authors of papers will be determined based on their contribution to this study.

**19. Change Management**

**19.1. Changes to Documents Approved by the Certified Clinical Research Review Board**

If changes occur to documents approved by the Certified Clinical Research Review Board, the overall administrator will apply for the change to the Certified Clinical Research Review Board. If an opinion is expressed by the Certified Clinical Research Review Board, the principal investigator will be promptly notified of the content of that opinion. The principal investigator will promptly report the content to the administrator of the implementing medical institution.

**19.2. Changes to the Implementation Plan**

When the overall administrator changes the implementation plan (excluding minor changes specified by the Ordinance of the Ministry of Health, Labour and Welfare), they will seek the opinion of the Certified Clinical Research Review Board stated in the implementation plan and submit the revised implementation plan and Form 2 notification in advance.

**19.3. Minor Changes to the Implementation Plan**

When the overall administrator makes minor changes to the implementation plan in accordance with Article 42 of the Clinical Research Act Enforcement Regulations, they will notify the Certified Clinical Research Review Board stated in the implementation plan and report it to the Minister of Health, Labour and Welfare within 10 days from the date of the change.

**20. Conflict of Interest**

**20.1. Funding Sources for This Study**

This study will be funded by research grants from **********. No financial support has been received from specific companies or organizations.

**20.2. Conflict of Interest Management**

This study will establish a conflict of interest management plan after factual confirmation at each implementing medical institution, and will conduct appropriate management after seeking the opinion of the Certified Clinical Research Review Board. The conflict of interest status for this study is as shown in Appendix 1.

**21. Periodic Reporting to the Certified Clinical Research Review Board and the Minister of Health, Labour and Welfare**

The overall administrator will report the status of clinical research to the Certified Clinical Research Review Board and the Minister of Health, Labour and Welfare, after reporting the following matters to the administrator of the implementing medical institution. The overall administrator will promptly notify the principal investigator that a periodic report has been submitted to the Certified Clinical Research Review Board. The principal investigator will promptly report the content to the administrator of the implementing medical institution.

**21.1. Periodic Reporting to the Certified Clinical Research Review Board**

**21.1.1. Reporting Items for Periodic Reports**

(1) The number of research participants who participated in this study.

(2) The status of occurrence of diseases and other conditions related to this study and their subsequent course.

(3) The status of occurrence of non-compliance with this Ordinance or the research protocol related to this study and the subsequent actions taken.

(4) Evaluation of the safety and scientific validity of this study.

(5) Matters concerning the involvement of pharmaceutical product manufacturers, etc., in this study.

**21.1.2. Timing of Periodic Reports**

Periodic reports to the Certified Clinical Research Review Board will, in principle, be submitted annually within two months after the expiration of each one-year period, starting from the date the implementation plan was submitted to the Minister of Health, Labour and Welfare.

**21.2. Periodic Reporting to the Minister of Health, Labour and Welfare**

**21.2.1. Reporting Items for Periodic Reports**

(1) The name of the Certified Clinical Research Review Board stated in the implementation plan.

(2) The Certified Clinical Research Review Board's opinion on the continuation of this study.

(3) The number of research participants who participated in this study.

**21.2.2. Timing of Periodic Reports**

Periodic reports to the Minister of Health, Labour and Welfare will be submitted within one month from the date the Certified Clinical Research Review Board expresses its opinion.

**22. Storage and Disposal of Materials and Records**

**22.1. Storage of Source Documents**

"Source documents" refer to original records and data concerning clinical findings, observations, and other activities obtained through the application of investigational products and medical care for research participants. The principal investigator or the administrator of the implementing medical institution will store the following documents, including source documents and study-specific documents, for inspection or audit by the Certified Clinical Research Review Board and regulatory authorities or their designated persons. These documents include the research participant identification code list, medical records, original signed and dated consent forms, and electronic copies of electronic case report forms including audit trails. Furthermore, the principal investigator or the administrator of the implementing medical institution will retain essential documents to be stored for a period of at least 5 years after the date of suspension or termination of this study. If the principal investigator makes corrections to the above documents, records, etc., they will record the name of the person who made the correction and the date of the correction, and store it along with the corrected record.

**22.2. Storage of Record Documents Required by Law**

The overall administrator will appropriately store records and materials related to the deliberation of this study for a period of at least 5 years after the date of suspension or termination of this study, ensuring that leakage, mixing, theft, or loss does not occur. The documents to be stored in this study are as follows:

(1) Documents describing matters that identify research participants.

(2) Documents describing matters related to medical care and examinations for research participants.

(3) Documents describing matters related to participation in this study.

(4) Documents describing matters related to the administration of investigational products to research participants.

(5) Documents describing matters related to the review and opinion services for this study received from the Certified Clinical Research Review Board.

(6) Research protocol, implementation plan, and documents related to explanation to research participants and their consent.

(7) Final report and other documents (or copies) prepared by the overall administrator in accordance with the provisions of the Clinical Research Act Enforcement Regulations.

(8) Documents related to monitoring.

(9) Source documents, etc., excluding (1) to (4) above.

(10) Contracts related to the implementation of this study.

(11) Documents describing the outline of investigational products used in this study.

(12) Other documents necessary for the implementation of this study.

If the overall administrator and the principal investigator make corrections to these records, they will record the name of the person who made the correction and the date of the correction, and store it along with the corrected record.

**22.3. Storage of Samples**

"Samples" refer to biological materials such as blood and tissues collected in accordance with this research protocol. The principal investigator or the administrator of the implementing medical institution will appropriately store samples in accordance with this research protocol or the sample storage procedures.

**22.4. Secondary Use of Samples and Information**

Samples and information obtained in this study may be used for different research purposes (secondary use). This will be stated in the informed consent document, explained to research participants, and consent will be obtained. In the case of secondary use, a new research protocol will be created as necessary, and implemented after obtaining approval from the ethics review committee that should be consulted for that research.

**22.5. Disposal Procedures and Methods**

When disposing of samples and information obtained from research participants in this study, and records and materials related to the deliberation of this study, the principal investigator will take necessary measures to ensure that specific individuals cannot be identified.

The administrator of the implementing medical institution will store samples, information, records, and materials to be stored until notified by the principal investigator that their storage is no longer necessary.

**23. Ownership of Research Results**

If intellectual property rights, such as patent rights, arise as a result of this study, those rights shall belong to **********.

**24. Implementation Structure**

**24.1. Overall Administrator**

**********

**24.2. Principal Investigator**

Refer to Appendix 2 (List of Implementing Medical Institutions and Principal Investigators)

**24.3. Randomization Manager**

**********

**24.4. Responsible Person of Statistical Analysis**

**********

**24.5. Responsible Person of Data Management**

**********

**24.6. Responsible Person of Monitoring**

**********

**24.7. Research Secretariat (Head)**

**********

**25. References**

1. National Cancer Center Research and Development Agency. Cancer Information Service. (Online) (Cited: December 11, 2024.) <https://hbcr-survival.ganjoho.jp/graph?year=2014-2015&elapsed=5&type=c11#h-title>.
2. Japanese Society of Gastroenterology. Digestive Intractable Cancer Series: Pancreatic Cancer.
3. Japan Pancreas Society Pancreatic Cancer Clinical Practice Guidelines Revision Committee. Pancreatic Cancer Clinical Practice Guidelines (2022 Edition). Kanehara Publishing Co., Ltd.
4. Unno M, Motoi F, Matsuyama Y, et al. Randomized phase II/III trial of neoadjuvant chemotherapy with gemcitabine and S-1 versus upfront surgery for resectable pancreatic cancer (Prep-02/JSAP-05). J Clin Oncol 2019;37 (4_suppl):189.
5. Eguchi H, Takeda Y, Takahashi H, et al. A prospective, open-label, multicenter phase 2 trial of neoadjuvant therapy using full-dose gemcitabine and S-1 concurrent with radiation for resectable pancreatic ductal adenocarcinoma. Ann Surg Oncol. 2019;26:4498-4505.
6. Yamada D, Kobayashi S, Takahashi H, et al. Results of a randomized clinical study of gemcitabine plus nab-paclitaxel versus gemcitabine plus S-1 as neoadjuvant chemotherapy for resectable and borderline resectable pancreatic ductal adenocarcinoma (RCT, CSGO-HBP-015). Ann Surg Oncol. 2024;31:4621-33.
7. Ren Q, Kao V, Grem JL. Cytotoxicity and DNA fragmentation associated with sequential gemcitabine and 5-fluoro-2'-deoxyuridine in HT-29 colon cancer cells. Clin Cancer Res. 1998;4:2811-8.
8. Hidalgo M, Castellano D, Paz-Ares L, et al. Phase I-II study of gemcitabine and fluorouracil as a continuous infusion in patients with pancreatic cancer. J Clin Oncol. 1999;17:585-92.
9. Di Costanzo F, Carlini P, Doni L, et al. Gemcitabine with or without continuous infusion 5-FU in advanced pancreatic cancer: a randomised phase II trial of the Italian oncology group for clinical research (GOIRC). Br J Cancer. 2005;93:185-9.
10. Evans DB, Rich TA, Byrd DR, et al. Preoperative chemoradiation and pancreaticoduodenectomy for adenocarcinoma of the pancreas. Arch Surg. 1992;127:1335-9.

**Appendix 1 (Conflict of Interest Matters)**

**Study Name:** A Randomized Controlled Trial of Chemoradiotherapy versus Chemotherapy for Resectable Pancreatic Cancer

**Overall Administrator:** **********

**COI (Involvement in Research) regarding pharmaceutical companies, etc., involved in this study:**

Not applicable

**Disclosable COI between pharmaceutical companies, etc., involved in the target drug of this study and the overall administrator, principal investigators, sub-investigators, etc.:**

Not applicable

**Appendix 2 (List of Implementing Medical Institutions and Principal Investigators)**

| **No.** | **Implementing Medical Institution** | **Location & Phone Number** | **Principal Investigator** | **Position** |
| --- | --- | --- | --- | --- |
| 1 | ********** | ********** | ********** | ********** |
| 2 | ********** | ********** | ********** | ********** |
| 3 | ********** | ********** | ********** | ********** |
| 4 | ********** | ********** | ********** | ********** |
| 5 | ********** | ********** | ********** | ********** |
| 6 | ********** | ********** | ********** | ********** |
| 7 | ********** | ********** | ********** | ********** |
| 8 | ********** | ********** | ********** | ********** |
| 9 | ********** | ********** | ********** | ********** |
| 10 | ********** | ********** | ********** | ********** |
| 11 | ********** | ********** | ********** | ********** |
| 12 | ********** | ********** | ********** | ********** |
| 13 | ********** | ********** | ********** | ********** |
| 14 | ********** | ********** | ********** | ********** |
| 15 | ********** | ********** | ********** | ********** |

First Edition (Created: June 30, 2025)
