# Supplementary material for: Study protocol: A randomized controlled trial of chemoradiotherapy versus chemotherapy as neoadjuvant therapy for resectable pancreatic cancer (CSGO-HBP-027)
Source: PLoS One. 2026 Mar 26;21(3):e0345459. doi: 10.1371/journal.pone.0345459 (PMC13020802; doi:10.1371/journal.pone.0345459)
Supplement: S2 File — (DOCX) [file pone.0345459.s003.docx]

研究計画書

切除可能膵癌に対する術前補助療法としての化学放射線療法と化学療法のランダム化比較試験

A Randomized Controlled Trial of Chemoradiotherapy Versus Chemotherapy as Neoadjuvant Therapy for Resectable Pancreatic Cancer (CSGO-HBP-027)

| 統括管理者 | ********** |
| --- | --- |
| 研究計画書番号 | CSGO-HBP-027 |
| 作成日 | 2026年2月26日 |
| 版数 | 第1.3版 |

版管理

| 版番号 | 作成日・改訂日 |
| --- | --- |
| 第1.0版 | 2025年6月30日 |
| 第1.1版 | 2025年8月12日 |
| 第1.2版 | 2025年9月18日 |
| 第1.3版 | 2026年2月26日 |

**概要**

1. **臨床研究の目的及び内容**

| 研究の目的 | 切除可能膵癌に対する術前治療としてのGS-RT療法の有効性を、GS療法を対照として検証的に検討する。 |
| --- | --- |
| 研究予定期間 | jRCT公表日〜2031年8月31日 |
| 計画研究対象者数 | 200例 |
| 試験デザイン | 多施設共同、無作為割付、非盲検、並行群間比較試験 |
| 対象疾患名 | 膵癌 |
| 選択基準 | 1. 組織診又は細胞診により通常型膵癌であることが確認されている者 2. 同意取得時の年齢が18歳以上 3. ECOG PS が0-1の者 4. 画像診断等により、切除可能膵癌と判断される者   少なくとも5mm厚以下のCTにて根治切除の可能性を評価する。必要に応じてMRI、超音波検査、PET/CT、腹腔鏡検査等を行う。下記のような画像診断所見を有する場合、切除可能膵癌と診断する。   1. 門脈/上腸間膜静脈に腫瘍の接触・浸潤を認めない、もしくは接触・浸潤が180度未満で認められるが合併切除可能である 2. 下大静脈に接触・浸潤していない 3. 大動脈、腹腔動脈、総肝動脈、上腸間膜動脈に腫瘍の接触、浸潤を認めない 4. 膵癌に対する初回治療として今回の治療を受ける者 5. 同意取得時から6か月以上の生存が期待される者 6. 消化管に腫瘍の浸潤を認めない者 7. 主要臓器（骨髄、肝、腎、肺等）の機能が保持されている者   ・白血球数　3,500/mm^3^以上、好中球数：2,000/mm^3^以上、血小板数：100,000/mm^3^以上、ヘモグロビン：9.0g/dL以上  ・総ビリルビン：2.0mg/dL以下  （ただし閉塞性黄疸にて減黄術を受けている症例は3.0mg/dL以下とする。）  ・AST(GOT)、 ALT(GPT)：150U/L以下  ・血清クレアチニン値：1.2mg/dL以下、クレアチニンクリアランス：60mL/min以上（Cockcroft-Gault式による推定も可とする）   1. 本研究への参加について文書による同意が本人から得られる者 |
| 除外基準 | （１） 下記を満たす切除不能膵癌症例   1. 遠隔臓器転移 2. 腹膜播種陽性、腹腔内細胞診陽性 3. 傍大動脈リンパ節転移 4. 180度以上の門脈/上腸間膜静脈への接触・浸潤がある場合 5. 大動脈、上腸間膜動脈、腹腔動脈、総肝動脈のいずれかに接触・浸潤がある場合   （２） 肺線維症又は間質性肺炎ならびにそれら既往歴を有する症例、又は、それを疑うCT所見を有する症例。重度の肺気腫や陳旧性炎症性変化が認められる症例、あるいは治療前呼吸機能検査にて著しく呼吸機能の低下を認める場合(例、％VC50%以下、1秒量１L未満など)  （３） 活動性の感染症（ウイルス性肝炎は除く）を有する者  （４） 重篤な合併症（心不全、腎不全、肝不全、出血性の消化性潰瘍、腸管麻痺、腸閉塞、コントロール不良な糖尿病など）を有する者  （５） 中等度以上（穿刺を必要とするなど）の腹水、胸水を認める者  （６） 活動性の重複癌（同時性重複癌又は無病期間が3年以内の異時性重複癌）を有する者。ただし、局所治療により治癒とされるCarcinoma in situ（上皮内癌）又は粘膜内癌相当の病変は活動性の重複癌と認めない  （７） フルシトシン、フェニトイン、ワルファリンカリウムを使用している者  （８） 妊娠中又は妊娠中の可能性がある、妊娠を希望している、あるいは授乳中である女性。パートナーの妊娠を希望する男性  （９） 重篤な薬剤性過敏症がある者  （１０） 他の介入研究に参加中の者  （１１） その他の理由で、研究責任医師又は研究分担医師が研究対象者として不適当と判断した者 |
| プロトコール治療 | 【ゲムシタビン】  GS療法群、GS-RT療法群ともに、体表面積に合わせ規定された投与量(1,000mg/m^2^)をday1、8、22、29に静脈内投与する。  【S-1】  (1) GS療法群  体表面積に合わせ規定された投与量（80mg/m^2^）を朝食後及び夕食後の1日2回に分けて、GEM投与の週に1週間内服する。つまり、第1～14、 22～35日目に経口投与する。  (2) GS-RT療法群  体表面積に合わせ規定された投与量（80mg/m^2^）を朝食後及び夕食後の1日2回に分けて、GEM投与の週に5日間内服する。つまり、第1～5、8～12、22～26、29～33日目に経口投与する。  【放射線療法：GS-RT療法群】  1日1回1.8Gy、週末（土、日、祝）を除く連続28日間に合計50.4Gy照射する。休日等で連続照射できない場合は、照射を順延し、総照射量を確保する。ただし、放射線治療の順延は2週間までとする。明らかな病態の増悪（臨床症状の悪化を含む）の場合は照射を中止する。 |
| 中止基準 | 【研究対象者ごとのプロトコール治療の中止】  （１）研究対象者からプロトコール治療中止の申し出があった場合  （２）病勢の進行が確認された場合  （３）合併症の発症又は増悪により、医師が治療の継続を困難と判断した場合  （４）有害事象により医師が治療の継続を困難と判断した場合  （５）研究対象者が転院した場合  （６）研究対象者が死亡した場合  （７）有害事象により化学療法が3週間以上順延した場合  （８）有害事象により放射線治療が2週間以上順延した場合（GS-RT群のみ）  （９）登録後、不適格症例であることが判明した場合  （１０） その他、研究責任医師又は研究分担医師がプロトコール治療の継続ができないと判断した場合  プロトコール治療中止後も、次項に該当する場合を除き、全症例で最終登録症例の登録日の３年後まで経過観察を行う。  【研究対象者ごとの研究の中止】  （１）研究対象者から研究中止の申し出があった場合  （２）不適格症例であることが判明した場合  （３）転居等、研究対象者の都合で必要な観察、検査の今後の実施が不可能であることが判明した場合  （４）その他、研究責任医師又は研究分担医師が研究を中止すべきであると判断した場合  【研究全体の中止】  （１）予想される有害事象（疾病等）が計画時の想定を著しく超える場合等、研究対象者の安全性又は本研究の実施に悪影響を及ぼす可能性のある新たな重大な情報を入手した場合  （２）研究対象者の登録が計画と比較して著しく遅い場合等、目標とする研究対象者数を達成することが極めて困難であると判断される場合  （３）認定臨床研究審査委員会から本研究を中止すべき旨の意見を受けた場合  （４）その他、本研究の中止又は中断を必要とする状況が発生した場合 |
| 主要評価項目 | 全生存期間 |
| 副次評価項目 | （１）切除率（=切除例数／割付例数 × 100）  （２）R0切除率（＝R0切除例数／切除例数 × 100）  （３）術前治療の組織学的効果  （４）無増悪生存期間  （５）Grade 3以上の有害事象 |

1. **研究の概略図（シェーマ）**


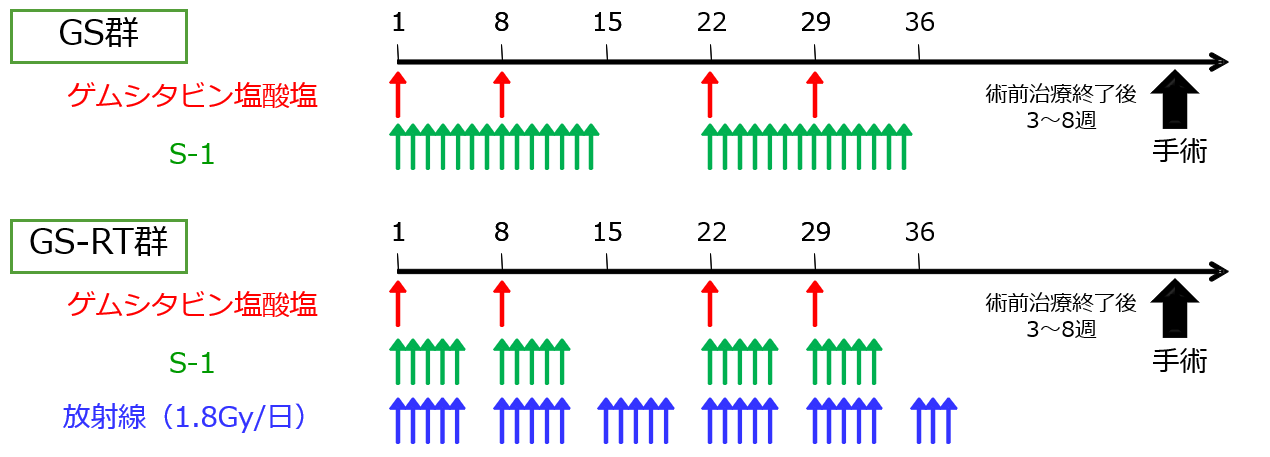


1. **観察・検査・評価のスケジュール**

|  | 登録時  （登録前1か月以内） | GEM投与前^a^  （投与前24時間以内） | 術前治療終了～手術直前^b^ | 手術後 | 手術後フォロー  （3か月毎）^c^ |
| --- | --- | --- | --- | --- | --- |
| 許容範囲 |  | 投与前日中 |  |  | ±1か月 |
| 研究対象者の背景 | ◎ |  |  |  |  |
| 診察（ECOG PS等） | ◎ | ◎ | ◎ |  | ◎ |
| 化学療法・放射線療法 |  |  |  |  |  |
| 血液検査（腫瘍マーカー以外） | ◎ | ◎ | ◎ |  | ◎ |
| 血液検査（腫瘍マーカー） | ◎ |  | ◎ |  | ◎ |
| CT | ◎ |  | ◎ |  | ◎ |
| 手術記録 |  |  |  | ◎ |  |
| 病理所見 |  |  |  | ◎ |  |
| 追跡調査 |  |  |  |  |  |
| 有害事象 |  |  |  |  |  |

◎：必須項目

a: 検査実施は必須だが、有害事象がみられた場合のみ症例報告書に記録する

b: この期間中に複数回検査を実施した場合、手術日に近い方のデータを症例報告書に記録する

c: 手術日から3年間は３か月毎に評価する。それ以降は、通常診療の間隔で評価する。

**目次**

[1. 略語・語句の定義 1](#_Toc223013498)

[1.1. 略語 1](#_Toc223013499)

[1.2. 語句の定義 1](#_Toc223013500)

[2. 研究の背景 2](#_Toc223013501)

[2.1. 対象疾患名 2](#_Toc223013502)

[2.2. 対象疾患の概念 2](#_Toc223013503)

[2.3. 切除可能膵癌に対する標準治療 2](#_Toc223013504)

[2.4. 本研究で検討する医薬品等 3](#_Toc223013505)

[2.5. 本研究を実施する意義 3](#_Toc223013506)

[3. 研究の目的及び評価項目 4](#_Toc223013507)

[3.1. 研究の目的 4](#_Toc223013508)

[3.2. 主要評価項目 4](#_Toc223013509)

[3.3. 副次評価項目 4](#_Toc223013510)

[4. 試験デザイン 5](#_Toc223013511)

[4.1. 試験デザイン 5](#_Toc223013512)

[4.2. 本研究の計画研究対象者数 5](#_Toc223013513)

[4.3. 本研究の予定実施期間 6](#_Toc223013514)

[5. 研究対象者の選定 7](#_Toc223013515)

[5.1. 選択基準 7](#_Toc223013516)

[5.2. 除外基準 7](#_Toc223013517)

[6. 研究の対象となる医薬品等 9](#_Toc223013518)

[6.1. 研究の対象となる医薬品等の概要 9](#_Toc223013519)

[6.2. 研究の対象となる医薬品等の品質の確保 9](#_Toc223013520)

[7. 研究対象者への医薬品等の適用方法 10](#_Toc223013521)

[7.1. 研究対象の医薬品等の適用方法 10](#_Toc223013522)

[7.2. 放射線療法 13](#_Toc223013523)

[7.3. 手術 16](#_Toc223013524)

[7.4. 後治療 17](#_Toc223013525)

[7.5. 支持療法・併用療法 17](#_Toc223013526)

[8. 時点ごとの観察、検査及び評価 20](#_Toc223013527)

[8.1. 観察・検査・評価スケジュール 20](#_Toc223013528)

[8.2. スタディーカレンダー 21](#_Toc223013529)

[9. 観察・検査及び評価の手順 22](#_Toc223013530)

[9.1. 同意取得 22](#_Toc223013531)

[9.2. 登録及び割付 22](#_Toc223013532)

[9.3. 研究対象者の背景 22](#_Toc223013533)

[9.4. ECOG-PS 23](#_Toc223013534)

[9.5. 化学療法 23](#_Toc223013535)

[9.6. 放射線療法 23](#_Toc223013536)

[9.7. 手術記録 23](#_Toc223013537)

[9.8. 病理所見 23](#_Toc223013538)

[9.9. 血液検査 24](#_Toc223013539)

[9.10. 後治療 24](#_Toc223013540)

[9.11. 追跡調査 24](#_Toc223013541)

[9.12. 研究対象者ごとの中止及び終了の基準 25](#_Toc223013542)

[10. 有害事象 27](#_Toc223013543)

[10.1. 有害事象の定義 27](#_Toc223013544)

[10.2. 有害事象の収集期間 27](#_Toc223013545)

[10.3. 有害事象の判定 27](#_Toc223013546)

[10.4. 有害事象の評価 27](#_Toc223013547)

[10.5. 有害事象が発現した場合の措置 28](#_Toc223013548)

[10.6. 本研究で予想される有害事象 29](#_Toc223013549)

[11. 疾病等 35](#_Toc223013550)

[11.1. 疾病等の定義 35](#_Toc223013551)

[11.2. 疾病等の認定臨床研究審査委員会等への報告手順 35](#_Toc223013552)

[11.3. 重篤な疾病等の厚生労働大臣への報告手順 36](#_Toc223013553)

[12. データマネジメント 37](#_Toc223013554)

[12.1. データマネジメント計画 37](#_Toc223013555)

[12.2. 中央モニタリング 37](#_Toc223013556)

[12.3. 症例報告書 37](#_Toc223013557)

[12.4. 症例報告書に直接記録されるデータ 37](#_Toc223013558)

[13. 統計的事項 38](#_Toc223013559)

[13.1. 解析集団の定義 38](#_Toc223013560)

[13.2. データの取り扱い 38](#_Toc223013561)

[13.3. 解析方法 38](#_Toc223013562)

[13.4. 中間解析及び早期中止に関する基準 40](#_Toc223013563)

[13.5. 統計解析計画の変更 40](#_Toc223013564)

[14. 品質管理及び品質保証 41](#_Toc223013565)

[14.1. 品質管理方針 41](#_Toc223013566)

[14.2. 品質目標 41](#_Toc223013567)

[14.3. モニタリング 41](#_Toc223013568)

[14.4. 規制当局等による調査への対応 41](#_Toc223013569)

[14.5. 不適合 41](#_Toc223013570)

[15. 倫理的配慮 43](#_Toc223013571)

[15.1. 遵守すべき諸規則 43](#_Toc223013572)

[15.2. 認定臨床研究審査委員会及び実施医療機関の管理者の承認 43](#_Toc223013573)

[15.3. 本研究における研究対象者の費用負担 43](#_Toc223013574)

[15.4. 同意説明文書及び研究対象者の同意 43](#_Toc223013575)

[15.5. 研究対象者からの相談窓口 44](#_Toc223013576)

[15.6. 研究対象者の予想される利益及び不利益 45](#_Toc223013577)

[15.7. 研究対象者の秘密保持（個人情報の保護） 45](#_Toc223013578)

[16. 健康被害に対する補償 46](#_Toc223013579)

[17. 臨床研究全体の中止又は終了 47](#_Toc223013580)

[17.1. 中止の基準 47](#_Toc223013581)

[17.2. 中止の手順 47](#_Toc223013582)

[17.3. 終了の基準 47](#_Toc223013583)

[18. 研究の情報公開及び結果公表 48](#_Toc223013584)

[18.1. 研究の登録 48](#_Toc223013585)

[18.2. 研究結果の公表 48](#_Toc223013586)

[19. 変更管理 49](#_Toc223013587)

[19.1. 認定臨床研究審査委員会で承認された書類の変更 49](#_Toc223013588)

[19.2. 実施計画の変更 49](#_Toc223013589)

[19.3. 実施計画の軽微な変更 49](#_Toc223013590)

[20. 利益相反 50](#_Toc223013591)

[20.1. 本研究に関する資金源 50](#_Toc223013592)

[20.2. 利益相反管理 50](#_Toc223013593)

[21. 認定臨床研究審査委員会、厚生労働大臣に対する定期報告 51](#_Toc223013594)

[21.1. 認定臨床研究審査委員会に対する定期報告 51](#_Toc223013595)

[21.2. 厚生労働大臣に対する定期報告 51](#_Toc223013596)

[22. 資料及び記録等の保管並びに廃棄方法 52](#_Toc223013597)

[22.1. 原資料の保管 52](#_Toc223013598)

[22.2. 法で定める記録文書の保管 52](#_Toc223013599)

[22.3. 試料の保管 53](#_Toc223013600)

[22.4. 試料・情報の二次利用について 53](#_Toc223013601)

[22.5. 廃棄の手順及び方法 53](#_Toc223013602)

[23. 研究結果の帰属 54](#_Toc223013603)

[24. 実施体制 55](#_Toc223013604)

[24.1. 統括管理者 55](#_Toc223013605)

[24.2. 研究責任医師 55](#_Toc223013606)

[24.3. 割付責任者 55](#_Toc223013607)

[24.4. 統計解析担当責任者 55](#_Toc223013608)

[24.5. データマネジメント担当責任者 55](#_Toc223013609)

[24.6. モニタリング担当責任者 55](#_Toc223013610)

[24.7. 研究事務局（責任者） 56](#_Toc223013611)

[25. 引用文献 57](#_Toc223013612)

#

# 略語・語句の定義

## 略語

| 略語 | 完全型 | 日本語表記 |
| --- | --- | --- |
| ALT | Alanine aminotransferase | アラニンアミノトランスフェラーゼ |
| AST | Aspartate aminotransferase | アスパラギン酸アミノトランスフェラーゼ |
| ALP | Alkaline phosphatase | アルカリホスファターゼ |
| GEM | Gemcitabine | ゲムシタビン |

## 語句の定義

1. 統括管理者

臨床研究法に規定する臨床研究を実施する者のうち、臨床研究の実施を統括管理する者をいう。

1. 研究責任医師

臨床研究法に規定する臨床研究を実施する者のうち、実施医療機関において臨床研究に係る業務を総括する医師をいう。

1. 研究分担医師

実施医療機関において、研究責任医師の指導の下に臨床研究に係る業務を分担する医師をいう。

1. モニタリング

臨床研究に対する信頼性の確保及び研究対象者の保護の観点から臨床研究が適正に行われていることを確保するため、本研究の進捗状況並びに本研究が臨床研究法、同法施行規則及び研究計画書に従って行われているかどうかについて、統括管理者が特定の者を指定して行わせる調査をいう。

1. 研究協力者

実施医療機関において、研究責任医師又は研究分担医師の指導の下にこれらの者の研究に係る業務に協力する薬剤師、看護師その他の医療関係者をいう。

# 研究の背景

## 対象疾患名

膵癌

## 対象疾患の概念

膵癌は早期診断が困難であり、診断時には進行癌であることが多いため、5年生存率は11.8%と予後不良な疾患である [1]。膵癌に対する唯一の根治的治療法は外科的切除であるが、適応となる患者は全体の20%程度と低い [2]。また切除された例でも早期の局所再発や遠隔転移再発のため治療成績は極めて不良である。このため手術を唯一の治療法とするのではなく、手術を主軸とした集学的治療を行うことに期待が寄せられている。

## 切除可能膵癌に対する標準治療

膵癌診療ガイドライン（2022年版） [3]では、切除可能膵癌は患者の状態が問題なければ外科的切除を行うのが標準治療であるとされている。しかし、膵癌は切除を行っても術後再発率が高く予後不良であるため、切除に加えた補助療法の併用が試みられ、切除可能膵癌に対する術前補助化学療法の予後改善効果がランダム化比較試験で証明され、これらの集学的治療が標準療法の位置づけとなった。

本邦で実施されたランダム化比較試験（Prep-02試験）でゲムシタビン塩酸塩（GEM）とテガフール・ギメラシル・オテラシルカリウム配合剤（S-1）を用いた術前化学療法を行った群の全生存率が、upfront surgery群に比較し有意に良好であると示されたことから [4]、膵癌診療ガイドラインでは、切除可能膵癌に対する術前化学療法としてGEM+S-1併用療法（GS療法）を行うことが提案されている。

一方、本研究で検討する術前化学放射線療法については、膵癌診療ガイドラインでは切除可能境界膵癌に対して行うことが提案されているが、切除可能膵癌に対する治療としては言及されていない。しかしながら、これまでに大阪大学消化器外科を中心とした多施設（大阪大学消化器外科共同研究会）からの前向き試験（CSGO-HBP-003） [5]にて、GS療法と放射線治療と併用した術前化学放射線療法（GS-RT療法）の治療成績が検討されており、単群設定の研究デザインであるために比較は困難であるが、良好な成績が報告されていることから、切除可能膵癌に対しても有用性が示唆される治療と言える。さらに最近、大阪大学消化器外科共同研究会にて実施された2つの試験（CSGO-HBP-003及びCSGO-HBP-015 [6]）に登録されていた対象患者の予後情報をもとにGS-RT療法とGS療法の成績を比較検討したところ、GS-RT療法の成績がGS療法よりも有意に良好であった（3年生存率：約55% vs 約35%）（論文投稿中）ことから、術前化学放射線療法が切除可能膵癌に対する標準治療となり得る可能性が示されている。

## 本研究で検討する医薬品等

GEMは代謝拮抗剤に分類される抗癌剤で、細胞内で三リン酸化物に代謝され、DNAの合成を阻害する。また、三リン酸化物濃度は細胞内で長時間維持され、固形癌に対して強い殺細胞作用を示す。

GEMは、進行膵癌に対する第一選択の抗癌剤として世界中で広く用いられており、本邦では第Ⅰ相試験が実施され、膵癌に対する適応が2001年4月に承認された。膵癌の他に、非小細胞肺癌、胆道癌、尿路上皮癌、手術不能又は再発乳癌、がん化学療法後に増悪した卵巣癌、再発又は難治性の悪性リンパ腫に対する適応を有している。

S-1は、5-FUのプロドラッグであるテガフールにモジュレーターであるギメラシルとオテラシルカリウムを配合した、経口の抗癌剤であり、5-FUの血中濃度を高めて抗腫瘍効果を増強すること、及びそれに付随して起こる消化管毒性を軽減することを目的として開発された。

遠隔転移を有する進行膵癌を対象とした前期第Ⅱ相試験（n=19）で、奏効率21.1%、time to progression（TTP）77日、生存期間中央値（MST）169日、後期第Ⅱ相試験（n=40）で、奏効率37.5%、TTP 113日、MST 281日という結果が得られ、膵癌に対する適応が2006年8月に承認された。その他、胃癌、結腸・直腸癌、頭頸部癌、非小細胞肺癌、手術不能又は再発乳癌、膵癌、胆道癌、ホルモン受容体陽性かつHER2陰性で再発高リスクの乳癌における術後薬物療法に対する適応を有している。

S-1の代謝物である5-FUとGEMは異なる経路でDNAの合成を阻害すること、及び相乗効果を有することが前臨床試験で知られている [7]。多くの臨床試験で膵癌に対する5-FUとGEMの併用療法の安全性と比較的良好な奏効割合（10-20％）や生存期間（中央値：7-10ヶ月）が報告されている [8] [9]。

## 本研究を実施する意義

前述のように、これまでに実施された2つの試験（CSGO-HBP-003及びCSGO-HBP-015）に登録されていた対象患者の予後情報をアップデートし、GS-RT療法とGS療法の成績を比較検討したところ、GS-RT療法の成績がGS療法よりも有意に良好であった（3年生存率：約55% vs 約35%）。術前化学放射線療法が切除可能膵癌に対する標準治療となり得る可能性が示されたことから、今回、ランダム化比較試験としてGS療法とGS-RT療法の有効性を比較する。

# 研究の目的及び評価項目

## 研究の目的

切除可能膵癌に対する術前治療としてのGS-RT療法の有効性を、GS療法を対照として検証的に検討する。

## 主要評価項目

全生存期間

【主要評価項目の設定根拠】

全生存期間は癌治療のtrue endpointであることから設定した。

## 副次評価項目

1. 切除率（=切除例数／割付例数 × 100）
2. R0切除率（＝R0切除例数／切除例数 × 100）
3. 術前治療の組織学的効果
4. 無増悪生存期間
5. Grade 3以上の有害事象

【副次評価項目の設定根拠】

（１）切除可能として本研究にエントリーしたものの、術前治療中の容態悪化（膵癌の進行や術前療法による有害事象等）により切除を計画することを断念せざるを得ない患者や、実際に切除を計画し手術を開始したが術中に同定された膵癌の進行などの所見により切除を中止せざるを得ない患者が生じることが想定されるため、切除に至った症例数を群間比較するために設定した。

（２）R0切除が可能であった症例数を群間比較するために設定した。

（３）術前治療による組織学的効果を群間比較するために設定した。

（４）手術実施までの増悪や手術後の再発について群間比較するために設定した。

（５）安全性を評価するために設定した。

# 試験デザイン

## 試験デザイン

下記の２群による、多施設共同、無作為割付、非盲検、並行群間比較試験。

1. 化学療法（GS）群
2. 化学放射線療法（GS-RT）群

割付にあたり、治療開始前CA19-9値（<370、≧370 U/mL）を割付因子とした層別ランダム化を適用する。


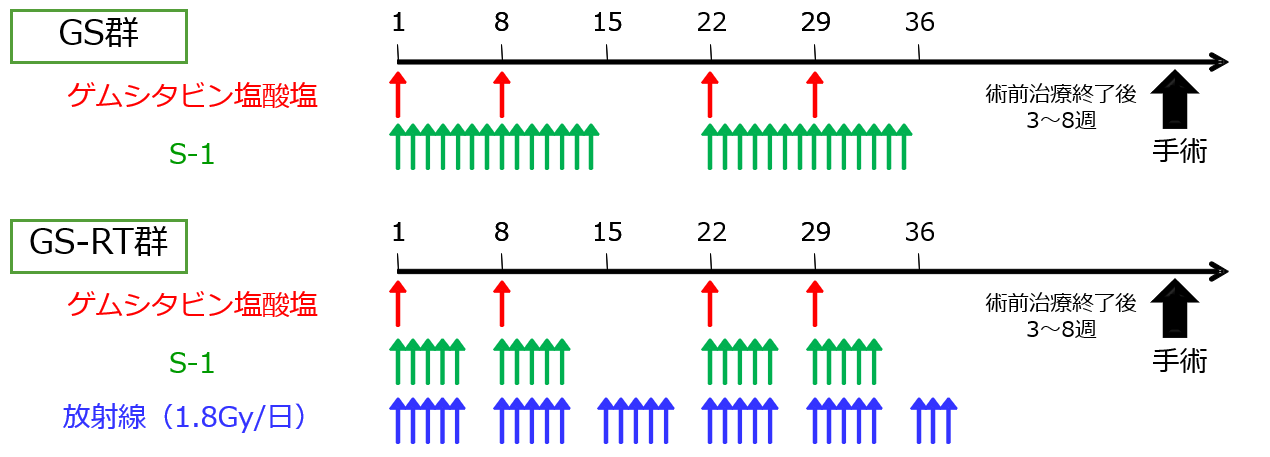


【試験デザインの設定根拠】

群間の患者背景の偏りを最小限とし比較可能性を高めるために、無作為割付を行う。治療開始前CA19-9値は腫瘍量を反映しており有効性評価に影響を及ぼす因子であると考えられることから割付因子とした。

## 本研究の計画研究対象者数

無作為割付する研究対象者数として合計200例

化学療法群：100例

化学放射線療法群：100例

【計画研究対象者数の設定根拠】

大阪大学消化器外科共同研究会により実施された、膵癌に対する2つの試験（CSGO-HBP-003及びCSGO-HBP-015）に登録された患者の予後情報を参考にした。これら試験から、3年生存率について化学療法群を35%、化学放射線療法群を55%と仮定し、フォローアップ期間3年、有意水準α＝0.05（両側）、検出力1-β=0.80と設定したもとで、ログランク検定による統計学的有意差に必要なサンプルサイズを計算すると各群91例であった。そのため、10％程度の脱落を考慮し、目標症例数（無作為割付する研究対象者数）は各群100例、両群合わせて200例と設定した。なお、計画研究対象者数の算出にはSAS version 9.4を用いた。

## 本研究の予定実施期間

1. 研究予定期間

jRCT公表日～2031年8月31日

1. 登録予定期間

jRCT公表日～2027年8月31日

1. 観察予定期間

jRCT公表日～2030年8月31日

全症例で最終登録症例の登録日の３年後まで経過観察を行う。

# 研究対象者の選定

選択基準をすべて満たし、除外基準に抵触しない患者を研究対象者とする。

## 選択基準

1. 組織診又は細胞診により通常型膵癌であることが確認されている者
2. 同意取得時の年齢が18歳以上
3. ECOG PS が0-1の者
4. 画像診断等により、切除可能膵癌と判断される者（切除可能膵癌の定義はNCCNガイドラインVersion 2.2025に準ずる）

少なくとも5mm厚以下のCTにて根治切除の可能性を評価する。必要に応じてMRI、超音波検査、PET/CT、腹腔鏡検査等を行う。下記のような画像診断所見を有する場合、切除可能膵癌と診断する。

1. 門脈/上腸間膜静脈に腫瘍の接触・浸潤を認めない、もしくは接触・浸潤が180度未満で認められるが合併切除可能である
2. 下大静脈に接触・浸潤していない
3. 大動脈、腹腔動脈、総肝動脈、上腸間膜動脈に腫瘍の接触、浸潤を認めない
4. 膵癌に対する初回治療として今回の治療を受ける者
5. 同意取得時から6か月以上の生存が期待される者
6. 消化管に腫瘍の浸潤を認めない者
7. 主要臓器（骨髄、肝、腎、肺等）の機能が保持されている者

・白血球数　3,500/mm^3^以上、好中球数：2,000/mm^3^以上、血小板数：100,000/mm^3^以上、ヘモグロビン：9.0g/dL以上

・総ビリルビン：2.0mg/dL以下

（ただし閉塞性黄疸にて減黄術を受けている症例は3.0mg/dL以下とする。）

・AST(GOT)、 ALT(GPT)：150U/L以下

・血清クレアチニン値：1.2mg/dL以下、クレアチニンクリアランス：60mL/min以上（Cockcroft-Gault式による推定も可とする）

1. 本研究への参加について文書による同意が本人から得られる者

【選択基準の設定根拠】

（１）～（７）は術前治療の有効性評価に適切な研究対象者を選定するために設定した。（８）は研究対象者のリスクを最小化するために設定した。（９）は適切な同意が得られた研究対象者で研究を実施するために設定した。

## 除外基準

1. 下記を満たす切除不能膵癌症例
2. 遠隔臓器転移
3. 腹膜播種陽性、腹腔内細胞診陽性
4. 傍大動脈リンパ節転移
5. 180度以上の門脈/上腸間膜静脈への接触・浸潤がある場合
6. 大動脈、上腸間膜動脈、腹腔動脈、総肝動脈のいずれかに接触・浸潤がある場合
7. 肺線維症又は間質性肺炎ならびにそれら既往歴を有する症例、又は、それを疑うCT所見を有する症例。重度の肺気腫や陳旧性炎症性変化が認められる症例、あるいは治療前呼吸機能検査にて著しく呼吸機能の低下を認める場合(例、％VC50%以下、1秒量１L未満など
8. 活動性の感染症（胆管炎及びウイルス性肝炎は除く）を有する者
9. 重篤な合併症（心不全、腎不全、肝不全、出血性の消化性潰瘍、腸管麻痺、腸閉塞、コントロール不良な糖尿病など）を有する者
10. 中等度以上（穿刺を必要とするなど）の腹水、胸水を認める者
11. 活動性の重複癌（同時性重複癌又は無病期間が2年以内の異時性重複癌）を有する者。ただし、局所治療により治癒とされるCarcinoma in situ（上皮内癌）又は粘膜内癌相当の病変は活動性の重複癌と認めない
12. フルシトシン、フェニトイン、ワルファリンカリウムを使用している者
13. 妊娠中又は妊娠中の可能性がある、妊娠を希望している、あるいは授乳中である女性。パートナーの妊娠を希望する男性
14. 重篤な薬剤性過敏症がある者
15. 他の介入研究に参加中の者
16. その他の理由で、研究責任医師又は研究分担医師が研究対象者として不適当と判断した者

【除外基準の設定根拠】

（１）は術前治療の有効性評価に不適切な研究対象者を除外するために設定した。（２）〜（５）、（８）、（９）は、研究対象者の安全性を確保するために設定した。（６）は有効性の評価に影響を与える可能性があるために設定した。（７）はS-1の併用禁忌薬又は併用注意薬であり研究対象者の安全性を確保するために設定した。（１０）は本研究及び参加中の介入研究に対して予測不能な影響を与える可能性があるために設定した。（１１）は研究責任医師又は研究分担医師が上記以外の理由で本研究には不適当と判断した者を除外するために設定した。

# 研究の対象となる医薬品等

## 研究の対象となる医薬品等の概要

本研究では以下の試験薬を用いる。

| 医薬品医療機器等法における未承認、  適応外、承認内の別 | □　未承認 | ■　適応外 | □　承認内 |
| --- | --- | --- | --- |
| 一般名称 | 注射用ゲムシタビン塩酸塩 | | |
| 販売名 | ジェムザール注射用200㎎、ジェムザール注射用1ｇ 他 | | |
| 製造販売会社名 | 日本イーライリリー株式会社　他 | | |
| 承認番号 | 21300AMY00405、21300AMY00404　他 | | |
| 保管の方法 | 室温保存 | | |

| 医薬品医療機器等法における未承認、  適応外、承認内の別 | □　未承認 | ■　適応外 | □　承認内 |
| --- | --- | --- | --- |
| 一般名称 | テガフール・ギメラシル・オテラシルカリウム配合剤 | | |
| 販売名 | ティーエスワン配合OD錠T20、ティーエスワン配合OD錠　他 | | |
| 製造販売会社名 | 大鵬薬品工業株式会社　他 | | |
| 承認番号 | 22500AMX00075000、22500AMX00076000　他 | | |
| 保管の方法 | 室温保存 | | |

## 研究の対象となる医薬品等の品質の確保

統括管理者は、以下に掲げる事項を実施する。

1　臨床研究に用いる医薬品等の品質が不良である等の情報を得たときには、その検証を行い、臨床研究の停止等の講ずる措置について、認定臨床研究審査委員会に報告する。また、その記録を作成する。

2　臨床研究に用いる医薬品等の品質が不良である等の理由により、医薬品等の回収が必要と判断したときは、速やかに認定臨床研究審査委員会に報告するとともに、以下の業務を行う。

（ア）研究責任医師に対し、医薬品等の使用中止と回収について速やかに通知する。

（イ）回収の内容、原因究明の結果及び改善措置を記載した回収処理記録を作成し、保存する。

研究責任医師は、以下に掲げる事項を実施する。

1　医薬品等の承認事項に基づく適切な管理を行う。

2　統括管理者から通知を受けた場合には、研究分担医師等に対し、医薬品等の使用中止と回収の指示を速やかに行う。

# 研究対象者への医薬品等の適用方法

本研究では、GEMやS-1の投与及び放射線療法をプロトコール治療として扱い、手術、後治療、支持療法・併用療法は通常診療として実施する。

無作為割付日から21日以内にプロトコール治療を開始する。

## 研究対象の医薬品等の適用方法

### GEM

1. GS療法群

体表面積に合わせ規定された投与量(1,000mg/m^2^)をday1、8、22、29に静脈内投与する。投与開始・継続基準、減量基準については7.1.4及び7.1.5参照（以下、同様）。

1. GS-RT療法群

体表面積に合わせ規定された投与量(1,000 mg/m^2^)をday1、8、22、29に静脈内投与する。

### S-1

1. GS療法群

体表面積に合わせ規定された投与量（80mg/m^2^）を朝食後及び夕食後の1日2回に分けて、GEM投与の週に1週間内服する。つまり、第1～14、 22～35日目に経口投与する。80mg/m^2^の投与量は以下の通りである。

| **体表面積** | **基準投与量** | |
| --- | --- | --- |
|  | **１日投与量** | **朝-夕** |
| 1.25m^2^未満 | 80mg/day | 40mg-40mg |
| 1.25 m^2^～1.50 m^2^未満 | 100mg/day | 50mg-50mg |
| 1.50 m^2^以上 | 120mg/day | 60mg-60mg |

1. GS-RT療法群

体表面積に合わせ規定された投与量（80mg/m^2^）を朝食後及び夕食後の1日2回に分けて、GEM投与の週に5日間内服する。つまり、第1～5、8～12、22～26、29～33日目に経口投与する。80mg/m^2^の投与量は以下の通りである。

| **体表面積** | **基準投与量** | |
| --- | --- | --- |
|  | **１日投与量** | **朝-夕** |
| 1.25m^2^未満 | 80mg/day | 40mg-40mg |
| 1.25 m^2^～1.50 m^2^未満 | 100mg/day | 50mg-50mg |
| 1.50 m^2^以上 | 120mg/day | 60mg-60mg |

### 研究対象の医薬品等の適用方法の設定根拠

GS療法の詳細は、Prep-02試験 [4]及びCSGO-HBP-015試験 [6]に基づいて決定した。

GS-RT療法の詳細は、CSGO-HBP-003試験 [5]に基づいて決定した。

### 術前化学療法の投与開始・継続基準

1. 第1日目及び第22日目の化学療法開始基準

第1日目及び第22日目の化学療法開始にあたっては、GEMの投与前24時間以内の検査値及び臨床所見が下記の投与継続基準を全て満たすことを確認の上、GEM及びS-1の投与を行う。

**第1日目及び第22日目の化学療法開始基準**

| **項目** | **第1日目の開始基準** | **第22日目の開始基準** |
| --- | --- | --- |
| 白血球数 | 3,500/mm^3^以上 | 3,000/mm^3^以上 |
| 好中球数 | 2,000/mm^3^以上 | 1,500/mm^3^以上 |
| 血小板数 | 100,000/mm^3^以上 | 75,000/mm^3^以上 |
| AST | 150 U/L以下 | 150 U/L以下 |
| ALT | 150 U/L以下 | 150 U/L以下 |
| 総ビリルビン | 2.0 mg/dL 以下 | 2.0 mg/dL 以下 |
| 血清クレアチニン | 1.2 mg/dL 以下 | 1.2 mg/dL 以下 |
| 下痢、口内炎 | Grade 1 以下 | Grade 1 以下 |
| その他の非血液学的毒性（脱毛・下痢を除く） | Grade 1 以下 | Grade 2 以下 |

1. 術前化学療法のコース内投与継続基準

コース内第8、29日目の化学療法投与においては、GEMの投与前24時間以内の検査値及び臨床所見が下記のコース内継続基準を全て満たすことを確認の上、GEM及びS-1の投与を行う。また、コース途中においても必要に応じて検査等を行い、コース内継続基準を満たしていることを確認して投与を継続する。

**コース内投与継続基準（第8、29日目）**

| **項目** | **コース内投与継続基準** |
| --- | --- |
| 白血球数 | 2,000/mm^3^以上 |
| 好中球数 | 1,000/mm^3^以上 |
| 血小板数 | 75,000/mm^3^以上 |
| 血清クレアチニン | 1.2mg/dL未満 |
| 下痢、口内炎 | Grade1以下 |
| その他の非血液学的毒性  （脱毛・下痢を除く） | Grade2以下 |

### 減量基準

当該コース内の有害事象発現状況により、以下の投与量変更基準に従い、上記投与継続基準を満たしたことを確認後1段階の減量により再開してよい。

以下の基準により、1度減量した症例において、さらに減量を要する有害事象が認められた場合は、さらなる減量は行わず、当該症例の研究を中止する。

**投与量変更基準**

| **項目** | **GEM** | **S-1** |
| --- | --- | --- |
| 好中球数 500/mm^3^未満（Grade4） | 1段階減量 | 1段階減量 |
| 発熱性好中球減少(Febrile neutropenia)  Grade3-4の好中球減少を伴う感染（38.5℃以上の発熱を伴う）が発現 | 1段階減量 | 1段階減量 |
| Grade3-4の好中球減少を伴う感染（Infection with G3 or G4 neutrophils）：胆管炎などが発現 | 1段階減量 | 1段階減量 |
| 血小板数 25,000/mm^3^未満（Grade4） | 1段階減量 | 1段階減量 |
| 血清クレアチニン　　　　1.2～1.5mg/dL未満  　　　　　　　　　　　　1.5mg/dl 以上 | 減量せず | 1段階減量 |
|  | 中止 | |
| Grade3以上の口内炎、下痢 | 減量せず | 1段階減量 |
| Grade3以上の非血液学的毒性（悪心・嘔吐、食欲不振、脱毛、疲労感を除く） | 1段階減量 | 1段階減量 |
| 有害事象の回復の遅延により、GEMの投与を延期又はskipした場合 | 1段階減量 | 減量せず |

1. GEMの減量基準

| **基準投与量** | **1段階減量投与量** |
| --- | --- |
| 1,000 mg/m^2^ | 800 mg/m^2^ |

1. S-1の減量基準

| **体表面積** | **基準投与量** | **1段階減量1日量** |
| --- | --- | --- |
| 1.25m^2^未満 | 80mg/day | 60 mg/day |
| 1.25 m^2^～1.50 m^2^未満 | 100mg/day | 80 mg/day |
| 1.50 m^2^以上 | 120mg/day | 100 mg/day |

## 放射線療法

### 放射線治療の照射量・照射方法

放射線療法は、1日1回1.8Gy、週末（土、日、祝）を除く連続28日間に合計50.4Gy照射する。休日等で連続照射できない場合は、照射を順延し、総照射量を確保する。ただし、放射線治療の順延は2週間までとする。明らかな病態の増悪（臨床症状の悪化を含む）の場合は照射を中止する。

1. 開始時期と休止期間等

放射線治療は化学療法開始後3日以内に開始する。放射線治療の開始は曜日を問わない。予定休止期間は設けない。祝祭日により照射が不可能となった場合は翌治療日に順延し、線量の変更は行わない。

1. 線量と分割法

1回1.8 Gy、1日1回、週5日、計28回、総線量50.4 Gy、総治療期間38日間、許容総治療期間60日とする。

1. 放射線治療装置

6 MV以上のX線発生装置を用いる。

1. 標的体積 (target volume)

肉眼的腫瘍体積(gross tumor volume: GTV)

造影CT又はMRI画像を参考し、設定する。

GTV primary: 原発巣。

GTV node: 　短径1cm以上のリンパ節。

臨床標的体積(clinical target volume: CTV)

CTV primary: subclinical extensionを考慮し、GTV primary＋0.5 cm。

CTV node: GTV nodeと同一。

CTV ENI: 膵臓後面組織、傍大動脈・腹腔動脈幹・総肝動脈・上腸間膜動脈のリンパ節領域。

・膵臓後面組織：背側境界は椎体前面の1cm背側。

・傍大動脈リンパ節領域：大動脈を中心に周囲1cmの範囲、頭側は腹腔動脈根部の0.5cm頭側、尾側は左腎静脈下縁まで。

・腹腔動脈幹、総肝動脈、上腸間膜動脈はこれを中心に周囲0.5cmの範囲とする。上腸間膜動脈の長軸方向はその根部から中結腸動脈分岐部までとする。

体内標的体積（internal target volume；ITV）

ITV: CTVに呼吸性移動を考慮した適切なマージンを加え(呼吸同期照射の場合は省略可)

計画標的体積(planning target volume: PTV)

PTV: ITVに患者固定再現性の誤差などを見込んで適切なマージンを加える(呼吸同期の場合はCTV＋マージン)。

1. 照射方法

PTVに対して通常分割照射（1.8 Gy×5 回/週）で50.4 Gyを投与する。照射野の形成にはMLC (multi-leaf collimator )を用いる。3門以上の多門照射または回転照射とする(3D-CRT、IMRT、VMAT)。

1. 線量分布計算

**a)標的規準点・処方点**

3D-CRTの場合：ビーム中心軸上の交点。

IMRTの場合：PTV D95

**b)** **PTV内の線量均一性・リスク臓器の線量制約**

・PTV：D98% > 90% かつ D2% < 110%

・脊髄：Dmax < 45 Gy

・腎臓：V18Gy < 35%

・肝臓：V30Gy < 40% かつ V20Gy < 67%

**c)線量分布図**

治療計画CTによる治療計画を行う。標的規準点を含む横断面の線量分布図を保存する。モニターユニット値の算出に不均一補正を行う。治療計画装置と計算方法のアルゴリズムを記録する。

1. 治療計画CT

撮影範囲：肝臓から両腎臓までを充分に含む範囲

造影剤：可能なら使用

スライス厚：5 mm以下

呼吸：自然呼吸で撮像する。呼吸性移動評価に自然吸気時・自然呼気時の2相、または4D-CTを推奨。

1. 画像照合

治療開始時及び照射方法の変更時に、治療装置による照合画像を必ず撮影する。毎回治療前の画像照合を行い照射(IGRT)することを推奨する。

### 放射線照射・休止基準及び再開基準

放射線照射のコース開始及びコース内照射基準は次の通りとする。この基準を満たさない場合は、回復するまで休止し、照射基準を満たしたことを確認の上、照射を再開する。その他、主治医が照射可能でないと判断した場合も照射を休止する。放射線照射休止中はS-1及びGEMの投与も休止する。放射線照射再開後は予定照射線量を施行し、総線量（50.4Gy / 28 fractions）を確保する。なお、放射線照射の休止は2週間までとする。

**放射線療法のコース内照射基準**

| **項目** | **放射線照射基準** | **回復後の再開線量** |
| --- | --- | --- |
| 白血球数 | 1,000/mm^3^以上（G3以下） | 規定線量 |
| 好中球数 | 500/mm^3^以上（G3以下） | 規定線量 |
| 血小板数 | 25,000/mm^3^以上 | 規定線量 |
| AST、ALT | 150IU未満 | 規定線量 |
| 放射線に伴う皮膚炎 | Grade 1 以下 | 規定線量 |
| その他の非血液学的毒性  （脱毛、下痢を除く） | Grade 2 以下 | 規定線量 |

### 放射線照射量の減量

原則として放射線照射量の減量は行わないものとする。

### GEM+S-1併用放射線療法の投与パターン

例1）Day 8　Grade 2の血小板減少で投与せず、Day10に回復

day8を含めて3日以内にコース内投与継続基準を満たした場合、投与を再開する。ただし、規定された投与スケジュールの変更は行わない。

例2）Day 8　Grade 2の血小板減少で投与せず、Day10に回復しなかった

day8を含めて3日以内にコース内投与継続基準を満たさなかった場合、スケジュール通り、day22に投与開始基準を満たしていることを確認の上、投与を再開する。

例3）Day 22　Grade 3の血小板減少で投与せず、Day 24に回復

day22を含めて3日以内にコース内投与継続基準を満たした場合、投与を再開する。ただし、TS-1は規定された投与スケジュールの変更は行わない。また、4週目と5週目のGEMの投与は7日間以上間隔をあける。

例4）Day 22　Grade 3の白血球減少で放射線を含めた全ての治療が休止し、Day36に回復

放射線療法休止後、2週間以内に放射線照射基準あるいは第22日目化学療法開始基準を満たした場合、規定された投与スケジュール通りに放射線及び化学療法の投与を再開する。ただし、放射線療法の休止は最大2週間までとする。

## 手術

最終抗癌剤投与日から原則として3～8週間以内に根治手術を予定する。手術適格基準は下記のごとくとする。適格性回復に長時間を要し、治療担当医により手術が危険であると判断された場合は、プロトコール終了とし、以後の治療は担当医の判断に委ねる。患者希望や毒性などにより予定された化学療法を施行できない場合でも、以下の手術適格基準を満たしていることを確認し、手術を施行してもよい。

手術適格基準

術前の画像所見、測定データ等により以下の全てを満たした場合、手術適格とする。

画像検査上、切除不能因子がない

1. PS　0-1　(ECOG　grade)
2. 白血球数：2,000/mm^3^以上
3. 好中球数：1,000/mm^3^以上
4. 血小板数：100,000/mm^3^以上
5. 総ビリルビン：2.0mg/dL以下

（ただし、閉塞性黄疸にて減黄術を受けている症例は3.0mg/dL以下とする。）

1. 体温、WBC、CRP上昇等の感染兆候がない。ただし、腫瘍に起因すると思われる感染兆候もしくは体温、WBC、CRPの上昇例は適格とする。

外科手術に当たっては、あらためて患者への説明をおこない、各施設の所定書式を用いて文書による同意を得る。

根治切除の可能性評価

少なくとも5mm厚以下のCTにて切除可能膵癌、切除可能境界膵癌と判断される場合に根治切除可能と判断する（切除可能膵癌、切除可能境界膵癌の定義はNCCNガイドラインVersion 2.2025に準ずる）。必要に応じてMRI、超音波検査、PET/CT、腹腔鏡検査等を行う。なお、画像検査において、根治切除が可能かどうかの判断は各施設の放射線科・外科・内科医の診断基準に委ねる。

## 後治療

術後補助療法としては膵癌診療ガイドライン（2022年版）に準じたS-1の内服を推奨するが、患者状況や主治医の判断を尊重し、手術後補助化学療法の規定はしない。しかし、その内容は症例報告書にて報告する。プロトコール治療中止の場合、後治療は規定しないが、その内容を症例報告書にて報告する。

## 支持療法・併用療法

### 許容される併用療法

高血圧や糖尿病等の合併症及び有害事象の対症療法を目的とした治療薬の併用は行ってもよいこととする。

### 許容されない併用療法

プロトコール治療中、腫瘍の増悪あるいは二次癌が確認されるまでは、GEM及びS-1以外の抗悪性腫瘍剤、ステロイド療法以外のホルモン療法、免疫療法などの併用療法は行わない。また、治験薬も併用しないこととする。

### 推奨される支持療法

以下の支持療法が推奨される。実施した場合には薬剤名、一日投与量、投与方法、投与期間及び使用理由等を症例報告書に記載する。

1）白血球（好中球）減少症：白血球減少は投与開始後1-2週間で発現し、投与継続すると重篤な感染をおこしやすい。重篤な白血球（好中球）減少をみた場合は注意深く経過観察し、発熱を伴う場合は感染巣の検索を行い、抗生剤を投与する。

保険適応内でG-CSFの併用可とするが、必ずGEM及びTS-1の投与を休止する（同時併用しない）。また、予防的投与は行わない。

**G-CSFの使用例(保険適応)**

| 開始時期 | ・好中球1,000/mm^3^未満で発熱（原則として38度以上）  ・好中球500/mm^3^未満が観察された時点  ・前コースで好中球1,000/mm^3^未満で発熱（原則として38度以上）、好中球500/mm^3^未満が観察された場合、同一の化学療法施行後、好中球1,000/mm^3^未満が観察された時期 |
| --- | --- |
| 使用量 | ・フィルグラスチム（グラン）：50 μg/m^2^を1日1回皮下投与、又は100 μg/m^2^を1日1回静脈投与  ・ナルトグラスチム（ノイアップ）：1 μg/kgを1日1回皮下投与、又は2 μg/kgを静脈投与  ・レノグラスチム（ノイトロジン）：2 μg/kgを1日1回皮下投与、又は5 μg/kgを1日1回静脈投与 |
| 中止時期 | ・好中球が最低値を示す時期を経過後5,000/mm^3^以上に達した場合は投与を中止する  ・好中球が2,000/mm^3^以上に回復し、感染症が疑われるような反応がなく、本剤に対する反応性から患者の安全が確保できると判断した場合には、本剤の中止、減量を検討する |

2）肝障害：安静臥床を基本とし、肝庇護剤の投与を行う。また、劇症化した場合は特に有効な治療法はないが類薬の場合ステロイド療法、G・I療法、血漿交換、特殊組成アミノ酸輸液が行われている。

3）重篤な腎障害：腎不全に陥った場合は注意深い水分管理、電解質補正、必要な場合は腎透析を行いながら尿量の回復を待つ。尿量が回復した後にはしばしば利尿期になるので重ねて注意する。

4）重篤な腸炎・脱水症状：脱水を伴う場合は十分な輸液を行い、阿片チンキ、リン酸コデインなどの止痢剤を投与する。重症の下痢にはロペラミドの投与も考慮する。下痢の発現は白血球減少を伴うことが多く、下痢の重症化は重症感染症の引き金となるので早期の対応が必要である。

5）悪心・嘔吐：悪心・嘔吐に関しては制吐剤の投与を考慮する。また、予測性の悪心･嘔吐には

ジアゼパムなどのマイナートランキライザーを投与する。

6）口内炎：口内炎に対してはアロプリノールの咳嗽やステロイド剤を投与する。

7）感染症：感染症に対しては、起因菌に対して適切な抗菌スペクトルを有する抗生剤の投与を行う。

# 時点ごとの観察、検査及び評価

研究責任医師又は研究分担医師は、以下の観察・検査・評価スケジュールに従って、データを収集する。

## 観察・検査・評価スケジュール

### 登録時

登録前1か月以内に実施した以下の検査の結果をもとに選択基準及び除外基準の判定を行い、適格であると判断された研究対象者を登録する。なお、同意取得前に通常診療として実施した検査の結果を使用することを可とする。

- 診察（ECOG PS等）
- 血液検査
- CT

### GEM投与前

GEM投与前24時間以内に以下を実施し、化学療法の開始や継続を判断する。

- 診察（ECOG PS等）
- 血液検査（腫瘍マーカー以外）

### 術前治療終了～手術直前

以下の検査等を実施し、手術の適格性を評価する（7.3項参照）。

- 診察（ECOG PS等）
- 血液検査
- CT

### 手術後

以下の評価を行う。

- 手術記録
- 病理所見

### 手術後フォロー（3か月毎）

以下の検査等を実施する。

- 診察
- 血液検査
- CT
- 追跡調査

## スタディーカレンダー

|  | 登録時  （登録前1か月以内） | GEM投与前^a^  （投与前24時間以内） | 術前治療終了～手術直前^b^ | 手術後 | 手術後フォロー  （3か月毎）^c^ |
| --- | --- | --- | --- | --- | --- |
| 許容範囲 |  | 投与前日中 |  |  | ±1か月 |
| 研究対象者の背景 | ◎ |  |  |  |  |
| 診察（ECOG PS等） | ◎ | ◎ | ◎ |  | ◎ |
| 化学療法・放射線療法 |  |  |  |  |  |
| 血液検査（腫瘍マーカー以外） | ◎ | ◎ | ◎ |  | ◎ |
| 血液検査（腫瘍マーカー） | ◎ |  | ◎ |  | ◎ |
| CT | ◎ |  | ◎ |  | ◎ |
| 手術記録 |  |  |  | ◎ |  |
| 病理所見 |  |  |  | ◎ |  |
| 追跡調査 |  |  |  |  |  |
| 有害事象 |  |  |  |  |  |

◎：必須項目

a: 検査実施は必須だが、有害事象がみられた場合のみ症例報告書に記録する

b: この期間中に複数回検査を実施した場合、手術日に近い方のデータを症例報告書に記録する

c: 手術日から3年間は３か月毎に評価する。それ以降は、通常診療の間隔で評価する。

# 観察・検査及び評価の手順

## 同意取得

研究責任医師又は研究分担医師は、本研究で必要な検査の実施前に15.4.1項に記載の手順で研究対象者から同意を取得する。

## 登録及び割付

### 登録及び割付手順

研究責任医師又は研究分担医師は、次の手順に従い研究対象者を登録する。

1. 研究責任医師又は研究分担医師は、同意を取得した患者についてWeb登録システムに必要事項を入力し、登録する。
   Web登録システムへの入力に関しては、研究責任医師又は研究分担医師の指示のもと、研究協力者が代行することも可とする。
2. Web登録システムにて、患者の適格性が判定され、適格である場合には当該研究対象者の治療群が決定される。割付にあたり、治療開始前CA19-9値（<370、≧370 U/mL）を割付因子とした層別ランダム化を適用する。
3. 研究責任医師又は研究分担医師は、Web登録システム上で、登録判定結果及び治療群を確認する。
4. 研究責任医師又は研究分担医師は、本登録後21日以内に当該研究対象者に割付けられた治療を開始する。

### 割付表の作成及び保管

割付責任者が割付手順を作成し、研究対象者の割付情報を管理する。

割付情報は安全な場所に保管され、研究対象者、研究責任医師及び研究分担医師を含め、全ての関係者が入手できないようにする。

## 研究対象者の背景

診察、CT等の結果をもとに下記項目を確認する。

- 性別
- 年齢
- 診断方法
- 組織分類
- TNM分類（UICC第8版）
- 主たる原発部位、主たる原発部位の大きさ
- 身長、体重、体表面積（身長と体重からDuBois式で算出）
- 閉塞性黄疸の有無
- 上記以外の併存症の有無

胆道ドレナージの有無

## ECOG-PS

診察結果をもとにECOG-PSを評価する。

## 化学療法

下記項目を確認する。

- 投与日
- 投与量

## 放射線療法

下記項目を確認する。

- 放射線照射日
- 1週間あたりの総照射線量

## 手術記録

下記項目を確認する。

- 腫瘍径
- 手術日
- 術式
- 合併切除部位
- 郭清リンパ節個数
- 出血量
- 手術時間
- 根治切除の有無
- Clavien Dindo 分類grade3以上の術後合併症の有無、内容
- 再手術の有無、再手術日

## 病理所見

- 腫瘍径
- pTNM分類（UICC第8版）
- 転移陽性リンパ節個数
- 組織学的遺残度
- 組織学的効果（Evans分類）

Evans分類 [10]

| GradeⅠ | Characteristic cytologic changes of malignancy are present, but little (< 10%) or no tumor cells are destroyed. |
| --- | --- |
| GradeⅡa | Destruction of 10%-50% of tumor cells. |
| GradeⅡb | Destruction of 51%-90% of tumor cells. |
| GradeⅢ | Few (< 10%) viable-appearing tumor cells are present. |
| GradeⅣ | No viable tumor cells are present. |

検索と判定は原則として病巣の中心を通る最大かつ面について判定することが望ましい。

## 血液検査

各実施医療機関の通常の手順で下記項目を測定する。

| 腫瘍マーカー | CA19-9、CEA、DUPANⅡ |
| --- | --- |
| その他 | ヘモグロビン、白血球、好中球数、血小板数、総ビリルビン、アルブミン、AST、ALT、ALP、尿素窒素、クレアチニン |

## 後治療

下記項目を確認する。

- 後治療の有無
- 後治療の種類
- 後治療の実施判断日
- 薬物治療名
- 術式
- 総照射線量
- 後治療の開始日、後治療の終了日

## 追跡調査

診察、腫瘍マーカー、CT等の結果をもとに下記項目を確認する。研究対象者が規定visitに来院しない場合には、可能な限り電話等で生死を確認する。

- 生死
- 死亡日又は直近で生存を確認した日
- 死亡理由
- 増悪の有無
- 部位
- 増悪判断日又は直近で無増悪と判断した日
- 増悪と判断した理由

## 研究対象者ごとの中止及び終了の基準

### 研究対象者ごとのプロトコール治療の中止

研究責任医師又は研究分担医師は、研究対象者の登録後に以下のいずれかに該当することが判明した場合、当該研究対象者に対するプロトコール治療を中止する。

プロトコール治療中止後も、9.12.2項に該当する場合を除き、全症例で最終登録症例の登録日の３年後まで経過観察を行う。

1. 研究対象者からプロトコール治療中止の申し出があった場合
2. 病勢の進行が確認された場合
3. 合併症の発症又は増悪により、医師が治療の継続を困難と判断した場合
4. 有害事象により医師が治療の継続を困難と判断した場合
5. 研究対象者が転院した場合
6. 研究対象者が死亡した場合
7. 有害事象により化学療法が3週間以上順延した場合
8. 有害事象により放射線治療が2週間以上順延した場合（GS-RT群のみ）
9. 登録後、不適格症例であることが判明した場合
10. その他、研究責任医師又は研究分担医師がプロトコール治療の継続ができないと判断した場合

### 研究対象者ごとの研究の中止

研究責任医師又は研究分担医師は、研究対象者の登録後に以下の事項に該当することが判明した場合、当該研究対象者に対する研究を中止する。

1. 研究対象者から研究中止の申し出があった場合
2. 不適格症例であることが判明した場合
3. 転居等、研究対象者の都合で必要な観察、検査の今後の実施が不可能であることが判明した場合
4. その他、研究責任医師又は研究分担医師が研究を中止すべきであると判断した場合

### 研究対象者ごとの中止の手順

研究責任医師又は研究分担医師は、中止基準に該当することが判明した場合には、ただちにその旨を研究対象者に説明し、必要に応じ代替治療等の処置を講じる。

研究責任医師又は研究分担医師は、本研究で計画されている観察・検査・評価を可能な範囲で実施し、中止理由とともにその結果を症例報告書に記録する。

### 研究対象者ごとの終了

本研究計画書に記載した計画したすべての観察・検査・評価が完了することをもって、当該研究対象者の終了とする。

# 有害事象

## 有害事象の定義

有害事象とは、研究対象者に生じた、あらゆる好ましくない或いは意図しない徴候（臨床検査値の異常を含む）、症状又は病気のことであり、本研究との因果関係の有無は問わない。研究開始前から認められ、投与後にその症状が増悪した場合も有害事象として扱う。

本研究ではCTCAE v5.0のGrade 3以上の有害事象の情報を症例報告書に記録する。

## 有害事象の収集期間

プロトコール治療の開始日から手術実施までに発生した有害事象の情報を症例報告書に記録する。

## 有害事象の判定

### 自覚症状及び他覚所見の確認

研究責任医師又は研究分担医師は、他覚所見に加えて研究対象者から自覚症状の発現の有無を確認する。

外来の場合は、来院時以外に発現した自覚症状、他覚所見についても研究対象者から聴取する。

## 有害事象の評価

研究責任医師又は研究分担医師は、収集期間中に観察された有害事象について以下を症例報告書に記録する。有害事象が複数観察された場合には、事象ごとに記録する。

1. 有害事象名
2. 発現日
3. 重症度
4. 研究との因果関係

### 有害事象名

各事象を診断名で記載する。その診断名に随伴する徴候（臨床検査値の異常、心電図の異常所見を含む）及び症状は別の有害事象としては記載しない。診断名が不明な場合は、適宜、その徴候又は症状を有害事象として記載する。

### 発現日

有害事象の発現日を以下の規準で判断する。

| 有害事象 | 発現日 |
| --- | --- |
| 徴候、症状、疾患（診断名）の場合 | 研究対象者又は研究責任医師又は研究分担医師が最初に有害事象の徴候、症状に気付いた日を記載する |
| 無症候性の疾患の場合 | 診断のために検査が実施され、診断が確定した日を記載する  検査所見から陳旧性の所見が見られる場合や、発生時期がおおよそ推定できる場合でも、診断が確定した日を記載する |
| 併存疾患の悪化の場合 | 研究対象者又は研究責任医師又は研究分担医師が最初に疾患、症状の悪化に気付いた日を記載する |
| 本研究の対象となる医薬品等を適用開始後の検査で異常となった場合 | 臨床的に問題があると判断される検査値異常が認められた検査日を記載する |
| 本研究の対象となる医薬品等を適用開始する時点の検査で異常が見られ、その後の検査で悪化した場合 | 検査値の推移より医学的判断で明らかな上昇、低下、増加、減少が認められた検査日を記載する |

### 重症度

有害事象の重症度をCTCAE v5.0 日本語訳 JCOG/JSCO版に従って評価する。

### 研究との因果関係

本研究の実施と有害事象との因果関係を次のように分類する。なお、因果関係なしと判定した場合は、判定理由を症例報告書に記録する。

| 関連あり | 時間的に明白な相関関係（適用中止後の経過を含む）がある。又は、原疾患、併存疾患、併用薬・併用療法等の他要因も推定されるが、本研究の対象医薬品等の適用による可能性も考えられる有害事象 |
| --- | --- |
| 関連なし | 本研究の対象医薬品等の適用との時間的に明白な相関関係がない。又は、疾患、併存疾患、併用薬・併用療法等の他要因によると十分に考えられる有害事象 |

## 有害事象が発現した場合の措置

### 研究対象者への処置

有害事象の発現に際しては、研究責任医師及び研究分担医師が適切な救急処置を施し、研究対象者の安全の確保に留意し、必要に応じ専門医師による診断・治療を受けさせることにより解決及び原因究明に努める。

### 有害事象の追跡調査

研究期間中に発現した有害事象は回復するか、又は臨床上必要がないと判断されるまで、可能な限り追跡する。入院あるいは外来、外来の場合の通院頻度及び検査項目等の方法は、該当する有害事象の種類及び程度により、研究責任医師又は研究分担医師が決定する。

## 本研究で予想される有害事象

### GM

GM製剤（ジェムザール）の添付文書（2024年12月改訂（第2版））に記載されている副作用は以下の通り。研究責任医師又は研究分担医師は投与にあたり、最新の添付文書を確認する。

(1) 重大な副作用

1) 骨髄抑制

白血球減少（72.6%、ただし、2000/μL未満の減少は17.5%）、好中球減少（69.2%、ただし、1000/μL未満の減少は32.1%）、血小板減少（41.4%、ただし、5万/μL未満の減少は4.2%）、貧血［ヘモグロビン減少（66.5%、ただし、8.0g/dL未満の減少は13.1%）、赤血球減少（52.6%）］等があらわれることがある。なお、高度な白血球減少に起因したと考えられる敗血症による死亡例が報告されている。

2) 間質性肺炎（1.0%）

間質性肺炎の発症あるいは急性増悪が疑われた場合には、直ちに本剤による治療を中止し、ステロイド治療等の適切な処置を行うこと。間質性肺炎に起因したと考えられる死亡例が報告されている。

3) アナフィラキシー（0.2%）

呼吸困難、血圧低下、発疹等の症状があらわれることがある。

4) 心筋梗塞（0.2%）

5) うっ血性心不全（頻度不明）

6) 肺水腫（頻度不明）

7) 気管支痙攣（頻度不明）

8) 成人呼吸促迫症候群（ARDS）（頻度不明）

9) 腎不全（頻度不明）

10) 溶血性尿毒症症候群（0.2%）

血小板減少、ビリルビン上昇、クレアチニン上昇、BUN上昇、LDH上昇を伴う急速なヘモグロビン減少等の微小血管症性溶血性貧血の兆候が認められた場合には、投与を中止すること。腎不全は投与中止によっても不可逆的であり、透析療法が必要となることもある。

11) 重度の皮膚障害（頻度不明）

中毒性表皮壊死融解症（Toxic Epidermal Necrolysis: TEN）、皮膚粘膜眼症候群（Stevens-Johnson症候群）、紅斑、水疱、落屑等の重度の皮膚障害があらわれることがある。

12) 肝機能障害、黄疸（頻度不明）

AST、ALT、Al-Pの上昇等の重篤な肝機能障害、黄疸があらわれることがある。

13) 白質脳症（可逆性後白質脳症症候群を含む）（頻度不明）

高血圧、痙攣、頭痛、視覚異常、意識障害等の症状が認められた場合には投与を中止し、適切な処置を行うこと。

(2) その他の副作用

|  | 10%以上 | 1～10%未満 | 1%未満 | 頻度不明 |
| --- | --- | --- | --- | --- |
| 循環器 |  | 頻脈、血圧上昇 | 血圧低下、狭心痛、動悸、心室性期外収縮、発作性上室頻拍、心電図異常(ST上昇) |  |
| 呼吸器 |  | 呼吸困難、高炭酸ガス血症^注1)^、低酸素血、咳嗽 | PIE(肺好酸球浸潤)症候群、喘鳴、喀痰、息切れ |  |
| 腎臓 | 総蛋白低下、電解質異常、アルブミン低下 | BUN上昇、蛋白尿、血尿、クレアチニン上昇 | 乏尿 |  |
| 消化器 | 食欲不振、悪心・嘔吐 | 下痢、便秘、口内炎、胃部不快感 | 歯肉炎 |  |
| 肝臓 | AST上昇、ALT上昇、  LDH上昇、Al-P上昇 | ビリルビン上昇、A/G比低下、γ-GTP上昇、ウロビリン尿 |  |  |
| 精神神経系 |  | 頭痛、めまい、不眠、知覚異常^注2)^ | 嗜眠、しびれ |  |
| 皮膚 | 発疹 | 脱毛^注2)^、そう痒感 | 蕁麻疹 |  |
| 注射部位 |  | 注射部位反応(静脈炎、疼痛、紅斑) |  |  |
| 血管障害 |  | 末梢性血管炎^注2)^ | 末梢性壊疽 |  |
| その他 | 疲労感、発熱、血小板増加 | 体重減少、尿糖陽性、好酸球増多、関節痛^注2)^、悪寒、味覚異常^注2)^、鼻出血、倦怠感^注2)^、浮腫、CRP上昇、体重増加、疼痛^注2)^、ほてり、胸部不快感 | 眼底出血、体温低下、耳鳴り、眼脂、無力症、顔面浮腫 | インフルエンザ様症状(倦怠感、無力症、発熱、頭痛、悪寒、筋痛、発汗、鼻炎等)、放射線照射リコール反応 |

注1) 膵癌の臨床試験11例における発現頻度である。

注2) 国内における本剤とパクリタキセルとの併用投与の臨床試験においては30%以上の頻度で認められている。

### S-1

S-1製剤（ティーエスワン）の添付文書（2025年1月改訂（第4版））に記載されている副作用は以下の通り。研究責任医師又は研究分担医師は投与にあたり、最新の添付文書を確認する。

(1) 重大な副作用

1) 骨髄抑制、溶血性貧血

汎血球減少、無顆粒球症（症状：発熱、咽頭痛、倦怠感等）（いずれも頻度不明）、白血球減少（46.7%）、貧血（頻度不明）、血小板減少（15.7%）等の重篤な骨髄抑制、溶血性貧血（頻度不明）があらわれることがある。

2) 播種性血管内凝固症候群（DIC）（0.4%）

血小板数、血清FDP値、血漿フィブリノゲン濃度等の血液検査に異常が認められた場合には投与を中止し、適切な処置を行うこと。

3) 劇症肝炎等の重篤な肝障害

劇症肝炎等の重篤な肝障害（B型肝炎ウイルスの再活性化によるものを含む）（頻度不明）があらわれることがある。

4) 脱水症状

激しい下痢があらわれ、脱水症状（頻度不明）まで至ることがあるので、このような症状があらわれた場合には投与を中止し、補液等の適切な処置を行うこと。

5) 重篤な腸炎（0.5%）

出血性腸炎、虚血性腸炎、壊死性腸炎等があらわれることがあるので、激しい腹痛・下痢等の症状があらわれた場合には投与を中止し、適切な処置を行うこと。

6) 間質性肺炎

間質性肺炎（0.3%）注1)（初期症状：咳嗽、息切れ、呼吸困難、発熱等）があらわれることがあるので、異常が認められた場合には投与を中止し、胸部X線等の検査を行い、ステロイド治療等の適切な処置を行うこと。

7) 心筋梗塞、狭心症、不整脈、心不全

心筋梗塞、狭心症、不整脈（心室頻拍等を含む）、心不全（いずれも頻度不明）があらわれることがあるので、胸痛、失神、動悸、心電図異常、息切れ等が認められた場合には投与を中止し、適切な処置を行うこと。

8) 重篤な口内炎（頻度不明）、消化管潰瘍（0.5%）、消化管出血（0.3%）、消化管穿孔（頻度不明）

異常が認められた場合には投与を中止し、腹部X線等の必要な検査を行い、適切な処置を行うこと。

9) 急性腎障害、ネフローゼ症候群（頻度不明）

10) 中毒性表皮壊死融解症（Toxic Epidermal Necrolysis：TEN）、皮膚粘膜眼症候群（Stevens-Johnson症候群）（いずれも頻度不明）

11) 白質脳症等を含む精神神経障害

白質脳症（意識障害、小脳失調、認知症様症状等を主症状とする）や意識障害、失見当識、傾眠、記憶力低下、錐体外路症状、言語障害、四肢麻痺、歩行障害、尿失禁、知覚障害（いずれも頻度不明）等があらわれることがある。

12) 急性膵炎（頻度不明）

腹痛、血清アミラーゼ値の上昇等があらわれた場合には投与を中止し、適切な処置を行うこと。

13) 横紋筋融解症

筋肉痛、脱力感、CK上昇、血中及び尿中ミオグロビン上昇を特徴とする横紋筋融解症（頻度不明）があらわれることがある。また、横紋筋融解症による急性腎障害の発症に注意すること。

14) 嗅覚脱失

嗅覚障害（0.1%）があらわれ、嗅覚脱失（頻度不明）まで至ることがある。

15) 涙道閉塞（頻度不明）

外科的処置に至った例が報告されている。流涙等の症状があらわれた場合には、眼科的検査を実施するなど適切な処置を行うこと。

16) 肝硬変（プロトロンビン時間延長、アルブミン低下、コリンエステラーゼ低下等）（頻度不明）

(2) その他の副作用

|  | 5%以上 | 0.1～5%未満 | 頻度不明 |
| --- | --- | --- | --- |
| 血液 | 白血球減少、好中球減少、血小板減少、赤血球減少、ヘモグロビン減少、ヘマトクリット値減少、リンパ球減少 | 出血傾向（皮下出血斑、鼻出血、凝固因子異常）、好酸球増多、白血球増多 |  |
| 肝臓 | AST上昇、ALT上昇、ビリルビン上昇、Al-P上昇 | 黄疸、尿ウロビリノーゲン陽性 |  |
| 腎臓 |  | BUN上昇、クレアチニン上昇、蛋白尿、血尿 |  |
| 消化器 | 食欲不振、悪心・嘔吐、下痢、口内炎、味覚異常 | 腸管閉塞、イレウス、腹痛、腹部膨満感、心窩部痛、胃炎、腹鳴、白色便、便秘、口角炎、口唇炎、舌炎、口渇 |  |
| 皮膚 | 色素沈着 | 紅斑、落屑、潮紅、水疱、手足症候群^注2)^、皮膚潰瘍、皮膚炎、脱毛、爪の異常、爪囲炎、単純疱疹、皮膚の乾燥・荒れ | 光線過敏症、DLE様皮疹 |
| 過敏症 | 発疹 | そう痒 |  |
| 精神神経系 | 全身倦怠感 | しびれ、頭痛、頭重感、めまい | ふらつき、末梢性ニューロパチー |
| 循環器 |  | 血圧低下、血圧上昇、心電図異常、レイノー症状 | 動悸 |
| 眼 |  | 流涙^注3)^、結膜炎、角膜炎、角膜びらん、眼痛、視力低下、眼乾燥 | 角膜潰瘍、角膜混濁、輪部幹細胞欠乏 |
| その他 | LDH上昇、総蛋白減少、アルブミン低下 | 発熱、全身熱感、鼻炎、咽頭炎、痰、糖尿、血糖値上昇、浮腫、筋肉痛、CK上昇、関節痛、電解質異常（血清ナトリウム上昇、血清ナトリウム低下、血清カリウム上昇、血清カリウム低下、血清カルシウム上昇、血清カルシウム低下、血清クロール上昇、血清クロール低下）、体重減少 | 血清アミラーゼ値上昇 |

発現頻度は承認時までの単独投与による臨床試験から算出した。

注1) 製造販売後調査において実施した非小細胞肺癌使用成績調査では間質性肺炎は0.7%（11/1669例）、放射線性肺臓炎・呼吸困難・呼吸不全等の肺障害は0.7%（12/1669例）であった。

注2) 前治療有乳癌においては、手足症候群21.8%と副作用発現率が高かった。

注3) 製造販売後に実施した切除不能又は再発胃癌症例を対象とした臨床試験のTS-1単独投与においては、流涙16.0%と副作用発現率が高かった。

### 放射線療法

膵癌に対する放射線療法で見られる副作用として一般的に知られているものは以下の通りであり、またこれらの副作用が治療から数年経過後に出現することもまれにある。

骨髄抑制、嘔気・嘔吐、食欲低下、消化管（胃、十二指腸、小腸、大腸等）の潰瘍・出血・狭窄、腎機能・肝機能障害、皮膚障害等

# 疾病等

## 疾病等の定義

疾病等とは、本研究の実施に起因するものと疑われる疾病、障害若しくは死亡又は感染症に加え、臨床検査値の異常や諸症状を含む。

## 疾病等の認定臨床研究審査委員会等への報告手順

疾病等が発生した場合は、担当医は速やかに研究責任医師に伝える。研究責任医師は、以下の各号に規定する期間内にそれまでに判明している範囲で第１報として統括管理者及び実施医療機関の管理者に報告する。

統括管理者は、次に掲げる事項を知ったときは、それぞれに定める期間内に認定臨床研究審査委員会に報告するとともに、他の研究責任医師にその旨を通知する。通知を受けた他の研究責任医師は、速やかにその内容を実施医療機関の管理者に報告する。

1．次のうち、本研究の実施によるものと疑われるものであって予測できないもの：7日

イ．死亡

ロ．死亡につながる恐れのある疾病等

2．次に掲げる事項：15日

イ．次のうち、本研究の実施によるものと疑われるもの（1に掲げるものを除く）

（１）死亡

（２）死亡につながる恐れのある疾病等

ロ．次のうち、本研究の実施によるものと疑われるものであって予測できないもの

（１）治療のために医療機関への入院又は入院期間の延長が必要とされる疾病等

（２）障害

（３）障害につながるおそれのある疾病等

（４）（１）から（３）まで並びに死亡及び死亡につながるおそれのある疾病等に準じて重篤である疾病等

（５）後世代における先天性の疾病又は異常

次のうち、本研究の実施によるものと疑われるもの（前号ロに掲げるもの及び効果安全性評価委員会が設置された特定臨床研究において発生したものを除く。）：30日

イ．治療のために医療機関への入院又は入院期間の延長が必要とされる疾病等

ロ．障害

ハ．障害につながるおそれのある疾病等

ニ．イからハまで並びに死亡及び死亡につながるおそれのある疾病等に準じて重篤である疾病等

ホ．後世代における先天性の疾病又は異常

4．本研究の実施によるものと疑われるもの（上記1～3以外）：法第十七条第一項の規定による認定臨床研究審査委員会への定期報告を行うとき

●認定臨床研究審査委員会への報告対象となる疾病等

| 疾病等 | 予測できないもの | 予測できるもの |
| --- | --- | --- |
| a.　死亡 | 7日 | 15日 |
| b.　死亡につながるおそれのある疾病等 | 7日 | 15日 |
| c.　治療のために医療機関への入院又は入院期間の延長が必要とされる疾病等 | 15日 | 30日 |
| d.　障害 | 15日 | 30日 |
| e.　障害につながるおそれのある疾病等 | 15日 | 30日 |
| f.　cからeまで並びに死亡及び死亡につながるおそれのある疾病等に準じて重篤である疾病等 | 15日 | 30日 |
| g.　後世代における先天性の疾病又は異常 | 15日 | 30日 |
| h.　その他の疾病等 | 定期報告 | 定期報告 |

研究責任医師又は研究分担医師は、転帰の変更等、疾病等の報告内容に変更が生じた場合、実施医療機関の管理者及び統括管理者に通知し、統括管理者は認定臨床研究審査委員会に報告する。

## 重篤な疾病等の厚生労働大臣への報告手順

統括管理者は、11.2項の1及び2のロの事項を知った時には、同項の表に定める期間内に、jRCTの疾病等報告画面より厚生労働大臣に報告する。

# データマネジメント

## データマネジメント計画

データマネジメント担当者は症例報告書をもとに、データベースの入力、データクリーニング、クエリの作成及び統計解析のためのデータセット作成を行う。欠測値、未記入値、誤記が疑われる異常値等に関して、データマネジメント担当者は、研究責任医師又は研究分担医師に問い合わせする。研究責任医師又は研究分担医師による回答又はデータ修正の内容を確認する。

## 中央モニタリング

モニタリング手順書に従って実施する。

## 症例報告書

研究責任医師又は研究分担医師は、同意を取得したすべての研究対象者について症例報告書を作成する。症例報告書としてEDCシステムを使用する。

データマネジメント担当者は、EDCシステムの使用に際し、研究責任医師及び研究分担医師を対象にトレーニングを提供する。

研究責任医師又は研究分担医師は、症例報告書の作成にあたり、EDCシステムにデータを直接記録する。

症例報告書の変更又は修正は、変更又は修正前後の情報、変更又は修正者、変更又は修正日及びその理由を記録した監査証跡として記録される。

研究責任医師は症例報告書が正確かつ完全に作成されているかを確認し、症例報告書の該当箇所に署名（電子署名）する。研究責任医師は、症例報告書に記録された全データに関する正確性と信頼性について全責任を負う。

## 症例報告書に直接記録されるデータ

以下は症例報告書に直接記録されたデータが原資料である。

・研究と有害事象との因果関係

・研究責任医師又は研究分担医師のコメント

・研究対象者ごとのプロトコール治療の中止の理由

・研究対象者ごとの中止の理由

・その他、診療録に記録されないデータ

# 統計的事項

## 解析集団の定義

本研究では、全登録例うち化学療法又は化学放射線療法を少なくとも1 回以上投与され、1時点以上の評価を実施した研究対象者による集団を解析対象集団とする。

## データの取り扱い

データの取扱いは以下のとおりとする。なお、統括管理者及び統計解析責任者が協議の上、データ固定前にすべての研究対象者に関するデータの解析上の取扱いを決定する。

### 規定された許容範囲外の測定値の取扱い

評価項目の測定許容範囲から逸脱したデータに関しては、統括管理者及び統計解析責任者が協議の上でその採否を決定する。なお、該当範囲内に複数のデータが存在する場合には、規定評価日との日数差の絶対値を算出し、絶対値が最小のものをその評価時期のデータとして採用する。なお、絶対値が同じ場合には個別（評価項目別）に検討する。手術後フォロー期のデータは本取扱いの対象外とする。

### 外れ値の取扱い

外れ値の取扱いは解析前に検討し決定する。変数によっては、適当な変数変換又は外れ値に大きく影響されない統計手法の適用を検討する。

### 欠測値の取扱い

欠測したデータについては補完しない。

### 追跡不能例の取り扱い

追跡不能となった研究対象者は、全生存期間又は無増悪生存期間の解析では、最終確認生存日又は増悪がないことが確認された最終日をもって打ち切りとして扱う。無増悪生存期間以外の副次的評価項目については、追跡不能例の取り扱い方法は中止時期によって異なる。切除率、R0切除率、術前治療の組織学的効果については、術前に追跡不能となった患者は解析から除外し、術中に追跡不能となった研究対象者は非切除例とみなす。術後に追跡不能となった研究対象者は解析に含める。グレード3以上の有害事象については、追跡不能となった研究対象者を含む全例を解析に含める。

## 解析方法

以下における治療群は、実際に投与された治療法に基づいて各研究対象者に対して決定された群とする。また、解析対象について、特に記載していない場合は、13.1.で定義した「解析対象集団」を用いる。有意水準は5%とする。副次評価項目での多重性については考慮しない。

解析にはJMP（解析時点で最新のバージョン）を用いる。

### 研究対象者の背景

解析項目　　：研究対象者の背景

解析方法　　：人口統計学的及びその他の特性について、治療群別に記述統計量を用いて要約する。連続変数については平均値、標準偏差、最小値、中央値、最大値を算出する。カテゴリ変数については頻度及び割合を算出する。

### 主要評価項目

解析項目　　：全生存期間

解析方法　　：全生存期間は、割付日を起算日とし、あらゆる原因による死亡日までの期間と定義する。観察期間中に死亡が観察されなかった生存例については、最終生存確認日をもって打ち切りとする。

主たる解析として、Kaplan-Meier法を用いて治療群ごとに生存曲線を作成する。Kaplan-Meier生存関数に基づき、3年生存割合と中央生存期間を推定する。この際、時点生存割合の95%信頼区間はGreenwoodの公式ならびに補対数対数変換、中央生存期間の95%信頼区間はBrookmeyer-Crowley法を用いることとする。そして、ログランク検定により生存曲線の群間比較を行う。さらに、治療開始前CA19-9値（<370、≧370 U/mL）を調整した、Cox比例ハザードモデルによる化学療法群に対する化学放射線療法群のハザード比の推定と層別ログランク検定を実施する。比例ハザード性についてはSchoenfeld残差を用いて評価する。

補足的解析として、治療群を割付された治療法に基づいて決定された群とした場合の「解析対象集団」を対象として、上記と同様の解析を行う。

サブグループ解析として、背景因子（性別、年齢等）、ECOG-PS、手術記録（腫瘍径、術式等）等で定義されるそれぞれのサブグループに対して、主たる解析と同様に調整ハザード比を推定し、95%信頼区間を算出する。また、フォレストプロットを作成する。

### 副次評価項目

解析項目　　：切除率

解析方法　　：切除率は、解析対象集団における切除症例数の割合と定義する。

治療群ごとに切除率の点推定値及び95%信頼区間を算出する。また、治療開始前CA19-9値（<370、≧370 U/mL）を調整したロジスティック回帰モデルによる切除率のオッズ比の点推定値、95%信頼区間、及び両側p値を算出する。

解析項目　　：R0切除率

解析対象集団：解析対象集団のうち切除症例

解析方法　　：R0切除率は、解析対象集団におけるR0切除症例数の割合と定義する。

治療群ごとにR0切除率の点推定値及び95%信頼区間を算出する。また、治療開始前CA19-9値（<370、≧370 U/mL）を調整したロジスティック回帰モデルによるR0切除率のオッズ比の点推定値、95%信頼区間、及び両側p値を算出する。

解析項目　　：術前治療の組織学的効果

解析対象集団：解析対象集団のうち切除症例

解析方法　　：治療群ごとにEvans分類の各Gradeの割合に対する点推定値及び95%信頼区間を算出する。また、帰無仮説「Evans分類のGradeの分布は化学放射線療法群と化学療法で等しい」に対するカイ二乗検定を行い、両側p値を算出する。さらに、Evans分類をI+IIaとIIb+III+IVに分け、治療開始前CA19-9値（<370、≧370 U/mL）を調整したロジスティック回帰モデルによるオッズ比の点推定値、95%信頼区間、及び両側p値を算出する。

解析項目　　：無増悪生存期間

解析方法　　：無増悪生存期間は、割付日を起算日とし、増悪と判断された日又はあらゆる原因による死亡日のうち早い方までの期間と定義する。観察期間中に増悪と判断されていない生存例については、増悪がないことが確認された最終日をもって打ち切りとする。

Kaplan-Meier法を用いて治療群ごとに生存曲線を作成する。Kaplan-Meier生存関数に基づき、3年無増悪割合と中央無増悪期間を推定する。この際、時点無増悪割合の95%信頼区間はGreenwoodの公式ならびに補対数対数変換、中央無増悪期間の95%信頼区間はBrookmeyer-Crowley法を用いることとする。そして、ログランク検定により生存曲線の群間比較を行う。さらに、治療開始前CA19-9値（<370、≧370 U/mL）を調整した、Cox比例ハザードモデルによる化学療法群に対する化学放射線療法群のハザード比の推定と層別ログランク検定を実施する。

解析項目　　：Grade3以上の有害事象

解析方法　　：治療群別に、Grade3以上の有害事象の発生割合の点推定値及び 95%信頼区間を算出する。また、治療開始前CA19-9値（<370、≧370 U/mL）を調整したロジスティック回帰モデルによるオッズ比の点推定値、95%信頼区間、及び両側p値を算出する。

有害事象の事象別、重症度別に発現例数及び発現割合を治療群別に集計する。研究との因果関係が否定できない有害事象についても同様の集計を行う。

## 中間解析及び早期中止に関する基準

本研究では中間解析は実施しない。

## 統計解析計画の変更

統括管理者は、本研究開始後に解析の変更や追加が生じた場合、その妥当性及び本研究の評価への影響を検討し、統計解析責任者と協議し、研究計画書を改訂し、解析計画の変更に至った経緯を本研究の総括報告書において説明する。

# 品質管理及び品質保証

## 品質管理方針

本研究は臨床研究法及び同法施行規則を遵守して実施する。したがって、これらの法令が要求する事項を満たすよう品質管理方針を定める。

## 品質目標

臨床研究法により要求される文書を確実に作成、保管するとともに、研究対象者の保護に関する事項を遵守する。主要評価項目が解析できる研究対象者数として10%の脱落（逸脱を含む）を許容する。

## モニタリング

統括管理者は、臨床研究に対する信頼性の確保及び臨床研究の対象者の保護の観点から臨床研究が適正に行われていることを確保するため、本研究の進捗状況並びに本研究が法規則及び研究計画書に従って行われているかどうかについて、モニタリング担当者を指名し、モニタリング手順書を作成したうえでモニタリングを実施させる。

モニタリング担当者は、モニタリング手順書に従いモニタリングを実施し、その記録（モニタリング報告書）を作成する。

研究責任医師又は実施医療機関の管理者は、モニタリング担当者が、原資料を閲覧できることを保証する。

## 規制当局等による調査への対応

研究責任医師又は実施医療機関の管理者は、認定臨床研究審査委員会又はその指名する者並びに厚生労働大臣又はその指名する者が調査を要求した場合にはそれを受け入れ、原資料及びその他必要な資料を閲覧できることを保証する。

## 不適合

### 不適合の定義

不適合とは、臨床研究法施行規則、研究計画書、手順書等の不遵守及び研究データの改ざん、ねつ造等をいう。不適合を管理するにあたっては、以下の手順に基づき、対応する。

### 不適合の管理手順

研究責任医師は、本研究において判明した不適合をすべて記録する。

研究分担医師は、不適合であることを知ったときは、速やかに研究責任医師に報告する。

研究責任医師は、不適合であることを知ったときは、速やかに統括管理者及び実施医療機関の管理者に報告する。

統括管理者は、不適合の発生について速やかに上記の報告をした研究責任医師以外の研究責任医師に情報提供するとともに、不適合の発生状況及びその後の対応について、認定臨床研究審査委員会に定期報告する。

### 重大な不適合

統括管理者は、特に重大な不適合が判明した場合は、すみやかに認定臨床研究審査委員会の意見を聴く。

特に重大な不適合とは、臨床研究の研究対象者の人権や安全性及び研究の進捗や結果の信頼性に影響を及ぼすものをいう。例えば、選択・除外基準や中止基準、併用禁止療法等の不遵守をいい、臨床研究の研究対象者の緊急の危険を回避するためその他医療上やむを得ない理由により研究計画書に従わなかったものについては含まない。下記に例示するような場合は、研究の内容にかかわらず、当該重大な不適合に当てはまると考えられる。

① 説明同意を取得していない場合

② 実施医療機関の管理者の許可を取得していない場合

③ 認定臨床研究審査委員会の意見を聴いていない場合

④ 研究計画からの逸脱によって研究対象者に健康被害が生じた場合

⑤ 研究データの改ざん又はねつ造があった場合

⑥ その他、認定臨床研究審査委員会が重大な不適合と判断した場合

統括管理者は認定臨床研究審査委員会に意見を聴いた際の資料をjRCTに掲載する。

# 倫理的配慮

## 遵守すべき諸規則

本研究は、ヘルシンキ宣言に基づいた倫理原則に則り、臨床研究法、同法施行規則及びその他関連通知に従って実施する。研究責任医師及び研究分担医師は、本研究計画書を遵守してプロトコール治療を実施する。

## 認定臨床研究審査委員会及び実施医療機関の管理者の承認

本研究を実施することの適否について認定臨床研究審査委員会が審査し承認を得て、実施医療機関の管理者による承認を得た後、jRCT公表後に実施する。

## 本研究における研究対象者の費用負担

本研究は通常診療範囲内で実施するため、研究参加に伴い研究対象者の金銭的負担が増えることはない。

研究対象者に対する謝金等の支払いは行わない。

## 同意説明文書及び研究対象者の同意

### 同意取得手順

統括管理者は、研究対象者の同意を得るに際し、同意説明文書を作成し、認定臨床研究審査委員会の承認を得る。

研究責任医師又は研究分担医師は、本研究に関して認定臨床研究審査委員会で承認された同意説明文書に基づいて研究対象者に説明し、十分に考える時間を研究対象者に与え、研究対象者が本研究の内容を十分に理解したことを確認したうえで、本研究への参加を依頼する。

研究対象者本人が同意した場合には、所定の同意書を用いて研究対象者本人による署名を取得する。

研究責任医師又は研究分担医師は、当該同意書に同意に関する説明を実施した医師の氏名、説明日及び同意取得日の記載があることを確認する。

研究対象者は、本研究の参加について同意した後であっても、いつでも研究対象者の自由意志で同意を撤回することができる。同意を撤回する場合には、可能な限り研究対象者は所定の同意撤回書に署名して、研究責任医師又は研究分担医師に提出する。

同意書、同意撤回書のコピーを研究対象者本人に手渡し、同意書、同意撤回書の原本は実施医療機関で定められた方法で適切に保管する。

研究責任医師又は研究分担医師は、すでに同意を取得している研究対象者の研究参加の意思に影響を与えるような情報に関して同意説明文書が改訂された場合は、認定臨床研究審査委員会で審査を受け承認された同意説明文書（改訂版）を用いて、研究対象者に説明し再同意取得をする。

### 同意説明文書に記載すべき事項

同意説明文書には、以下の内容を含むものとする。

1. 研究の名称及び当該研究の実施について実施医療機関の管理者の承認を受けていること及び厚生労働大臣に実施計画を提出していること
2. 統括管理者の氏名又は名称、研究責任医師の氏名及び職名並びに実施医療機関の名称
3. 本研究の目的及び意義
4. 医薬品等の概要
5. 本研究の方法（研究対象者から取得された試料・情報の利用目的を含む）及び期間
6. 研究対象者として選定された理由
7. 研究対象者に生じる負担並びに予測されるリスク及び利益
8. 本研究が実施又は継続されることに同意した場合であっても随時これを撤回できる旨
9. 本研究が実施又は継続されることに同意しないこと又は同意を撤回することによって研究対象者等が不利益な取扱いを受けない旨
10. 本研究に関する情報公開の方法
11. 研究対象者等の求めに応じて、他の研究対象者等の個人情報等の保護及び当該研究の独創性の確保に支障がない範囲内で研究計画書及び本研究の方法に関する資料を入手又は閲覧できる旨並びにその入手又は閲覧の方法
12. 個人情報等の取扱い（特定の個人を識別することができないようにする場合にはその方法を含む）
13. 試料・情報の保管及び廃棄の方法
14. 本研究の資金源等、研究機関の研究に係る利益相反及び個人の収益等、研究者等の研究に係る利益相反に関する状況
15. 研究対象者等及びその関係者からの相談等、苦情及び問合せへの対応
16. 研究対象者等への経済的負担又は謝礼について
17. 他の治療方法の有無及び内容並びに他の治療法により予期される利益及び不利益に関する事項
18. 本研究によって生じた健康被害に対する補償の有無及びその内容
19. 特定臨床研究の審査意見業務を行う認定臨床研究審査委員会における審査事項その他当該特定臨床研究に係る認定臨床研究審査委員会に関する事項
20. その他特定臨床研究の実施に関し必要な事項

## 研究対象者からの相談窓口

研究対象者及びその関係者からの質問は、研究責任医師又は研究分担医師が対応する。

## 研究対象者の予想される利益及び不利益

### 予想される利益

GS療法は通常診療で実施されている治療であるため、通常診療と同等の利益が得られる。GS-RT群の場合、化学療法の投与パターンは若干異なるものの、GS療法を実施するため、通常診療とほぼ同等の利益は得られる。本研究はレトロスペクティブな比較で示唆されたGS療法に放射線療法を併用することで切除可能膵癌に対する治療効果が高まる可能性をランダム化比較試験で検証するものであり、本研究によって切除可能膵癌に対するGS-RT療法の有効性や安全性が明らかになれば、今後の切除可能膵癌患者に新たな治療選択肢を提供することができる。

### 予想される不利益

本研究で実施する各種検査は通常診療の範囲内であり、研究参加に伴い研究対象者の負担が増えることはない。GS療法は通常診療で実施されている治療であるため、通常診療を超える不利益はないが、GS-RT群の場合、追加される放射線療法により、10.6項に記載した副作用が発現する可能性がある。なお、GS群と比較して、わずかながらS-1の投与量が少ないことが化学療法の効果を減弱させる可能性があるが、GS-RT群で行われる術前治療全体から考えると、この差はわずかなものであると考えられることから、その可能性は低いと考えられる。

### 利益と不利益の総合評価及び不利益を最小化する対策

GEM、S-1及び放射線療法ともに副作用の発現状況や副作用への対処法の情報が集積されており、不利益が利益を上回る可能性は低い。なお、本研究に参加する前に研究対象者に十分に説明したうえで、その意思を確認する。

## 研究対象者の秘密保持（個人情報の保護）

本研究の実施に係る生データ類及び同意書等を取扱う際は、研究対象者の秘密保護に十分配慮する。同意を取得した研究対象者に対して研究対象者識別コードを付与する。研究対象者識別コードは、イニシャルやカルテID等のような特定の個人を識別できる情報とは無関係の数字記号等で構成され、症例報告書等の本研究に関する書類を作成する際には研究対象者識別コードを使用することで特定の個人を識別することができないようにする。研究責任医師は、研究対象者の氏名等の個人情報が外部に漏洩することがないよう対応表等の保管管理を厳重に行う。研究の結果を公表する際は、研究対象者を特定できる情報を含まないようにする。

# 健康被害に対する補償

研究責任医師、研究分担医師及び実施医療機関は、本研究の実施に起因して研究対象者に健康被害が発生した場合には、研究対象者がただちに適切な診断、治療及び必要な措置を受けることができるよう医療の提供その他必要な措置を講じる。

治療に要する費用は、通常の保険診療と同様に扱うものとし、臨床研究保険には加入せず、特別な補償は行わない。

# 臨床研究全体の中止又は終了

## 中止の基準

統括管理者は、下記に該当する場合、必要に応じて研究を中止又は中断する。

1. 予想される有害事象（疾病等）が計画時の想定を著しく超える場合等、研究対象者の安全性又は本研究の実施に悪影響を及ぼす可能性のある新たな重大な情報を入手した場合
2. 研究対象者の登録が計画と比較して著しく遅い場合等、目標とする研究対象者数を達成することが極めて困難であると判断される場合
3. 認定臨床研究審査委員会から本研究を中止すべき旨の意見を受けた場合
4. その他、本研究の中止又は中断を必要とする状況が発生した場合

## 中止の手順

統括管理者は、中止基準に該当することが判明し、本研究全体の中止を決定した場合、速やかに研究責任医師に通知する。

研究責任医師又は研究分担医師は、研究対象者に研究中止について説明し、可能な範囲で本研究において計画されている観察・検査・評価を実施するとともに必要に応じて代替治療等の医療上の処置を講じる。

統括管理者は、本研究全体の中止を決定した日から10日以内に認定臨床研究審査委員会に中止通知書を提出するとともに、厚生労働大臣に届け出る。

## 終了の基準

統括管理者が、総括報告書の概要をjRCTに記録することにより公表した日を本研究が終了した日とする。

# 研究の情報公開及び結果公表

## 研究の登録

統括管理者は、本研究の実施に先立ち、jRCTに登録する。研究計画書の変更及び研究の進捗に応じて適宜更新する。

## 研究結果の公表

### 主要評価項目報告書

統括管理者は、主要評価項目に係るデータの収集を行うための期間が終了した日から原則として１年以内に主要評価項目報告書を作成し、認定臨床研究審査委員会に意見を聴き、研究責任医師に通知する。研究責任医師は当該通知の内容を実施医療機関の管理者に報告する。統括管理者は、認定臨床研究審査委員会が意見を述べた日から起算して1月以内にjRCTに記録することで公表する。

なお、本研究では主要評価項目報告書の作成が総括報告書作成と同時期になる予定であるため、総括報告書の作成により主要評価項目報告書を作成したものとみなす。

### 総括報告書

統括管理者は、すべての評価項目に係るデータの収集を行うための期間が終了した日から原則として１年以内に総括報告書及びその概要を作成する。

総括報告書には少なくとも以下の事項を含めて作成する。

（１）臨床研究の対象者の背景情報（年齢、性別等）

（２）臨床研究のデザインに応じた進行状況に関する情報（対象者数の推移等）

（３）疾病等の発生状況のまとめ

（４）主要評価項目及び副次評価項目のデータ解析及び結果

統括管理者は、総括報告書及びその概要について認定臨床研究審査委員会に意見を聴き、研究責任医師に通知する。研究責任医師は当該通知の内容を実施医療機関の管理者に報告する。統括管理者は、認定臨床研究審査委員会が意見を述べた日から起算して1月以内に、当該総括報告書の概要、研究計画書及び統計解析計画書（作成した場合）をjRCTに記録することで公表する。

### 学会等の公表

本研究から得られた成果について、速やかに学会発表や論文投稿による公表を行う。公表する際は、研究対象者等及びその関係者の人権や権利利益の保護のために必要な措置を講じた上で公表する。学会発表者及び論文著者は、本研究への貢献度に基づき決定する。

# 変更管理

## 認定臨床研究審査委員会で承認された書類の変更

認定臨床研究審査委員会で承認された書類から変更が生じた場合、統括管理者は、認定臨床研究審査委員会に変更申請する。認定臨床研究審査委員会から意見が述べられた場合には、速やかに、その意見の内容について研究責任医師に通知する。研究責任医師は、速やかにその内容を実施医療機関の管理者に報告する。

## 実施計画の変更

統括管理者は、実施計画を変更（厚生労働省令で定める軽微な変更を除く。）するときは、当該実施計画に記載されている認定臨床研究審査委員会の意見を聴き、あらかじめ変更後の実施計画及び様式第二による届書を提出して行う。

## 実施計画の軽微な変更

統括管理者は、実施計画について、臨床研究法施行規則第四十二条に則り、軽微な変更をしたときは、その変更の日から 10 日以内に、その内容を、当該実施計画に記載されている認定臨床研究審査委員会に通知するとともに、厚生労働大臣に届け出る。

# 利益相反

## 本研究に関する資金源

本研究は、**********の研究費を財源として実施する。特定の企業・団体から資金援助は受けていない。

## 利益相反管理

本研究は、各々の実施医療機関において事実確認を受けた上で、利益相反管理計画を作成し、認定臨床研究審査委員会の意見を聴き適切な管理を行う。

本研究の利益相反状況は別紙1のとおり。

# 認定臨床研究審査委員会、厚生労働大臣に対する定期報告

統括管理者は、臨床研究の実施状況について、次に掲げる事項について、実施医療機関の管理者に報告した上で、認定臨床研究審査委員会、厚生労働大臣に報告する。

統括管理者は認定臨床研究審査委員会に定期報告を行ったことを速やかに研究責任医師に通知する。研究責任医師は、速やかに、その内容を実施医療機関の管理者に報告する。

## 認定臨床研究審査委員会に対する定期報告

### 定期報告における報告事項

（１）本研究に参加した研究対象者の数

（２）本研究に係る疾病等の発生状況及びその後の経過

（３）本研究に係るこの省令又は研究計画書に対する不適合の発生状況及びその後の対応

（４）本研究の安全性及び科学的妥当性についての評価

（５）本研究に対する医薬品等製造販売業者等の関与に関する事項

### 定期報告の時期

認定臨床研究審査委員会への定期報告は、原則として、実施計画を厚生労働大臣に提出した日から起算して、１年ごとに、当該期間満了後２月以内に行う。

## 厚生労働大臣に対する定期報告

### 定期報告における報告事項

（１）実施計画に記載されている認定臨床研究審査委員会の名称

（２）認定臨床研究審査委員会による本研究の継続の適否

（３）本研究に参加した研究対象者の数

### 定期報告の時期

厚生労働大臣への定期報告は、認定臨床研究審査委員会が意見を述べた日から起算して、１月以内に行う。

# 資料及び記録等の保管並びに廃棄方法

## 原資料の保管

「原資料」とは、研究対象者に対する医薬品等の適用及び診療により得られた臨床所見、観察その他の活動に関する元の記録やデータをいう。

研究責任医師又は実施医療機関の管理者は、認定臨床研究審査委員会及び規制当局又はその指名する者による調査又は監査のため、原資料及び本研究固有の文書を含む以下の資料を保管する。

これらの資料には、研究対象者識別コードリスト、医療記録、署名・日付入り同意書原本、監査証跡を含む電子症例報告書の電子的コピー等が該当する。

また、研究責任医師又は実施医療機関の管理者は、保管すべき必須文書を、本研究の中止又は終了後少なくとも5年が経過した日までの期間、保存する。

研究責任医師は上記の文書、記録等の修正を行う場合は、修正者の氏名及び修正を行った年月日を記録し、修正した記録とともに保存する。

## 法で定める記録文書の保管

統括管理者は、本研究の審議に関する記録及び資料等は、漏えい、混交、盗難、紛失等が起こらないよう、本研究の中止又は終了後少なくとも5年が経過した日までの期間、適切に保管する。本研究で保管する文書は下記のとおり。

（１）研究対象者を特定する事項を記載した文書

（２）研究対象者に対する診療及び検査に関する事項を記載した文書

（３）本研究への参加に関する事項を記載した文書

（４）研究対象者への研究対象とした医薬品等の投与に関する事項を記載した文書

（５）認定臨床研究審査委員会から受領した本研究に関する審査意見業務に係る事項を記載した文書

（６）研究計画書、実施計画、研究対象者に対する説明及びその同意に係る文書

（７）総括報告書、その他の臨床研究法施行規則の規定により統括管理者が作成した文書（又は写）

（８）モニタリングに関する文書

（９）上記（１）～（４）を除く原資料等

（10）本研究の実施に係る契約書

（11）本研究に用いる医薬品等の概要を記載した文書

（12）上記のほか本研究を実施するために必要な文書

なお、統括管理者及び研究責任医師は、これらの記録の修正をする場合は、修正者の氏名及び修正した年月日を記録し、修正した記録とともに保存する。

## 試料の保管

「試料」とは、本研究計画書に従って収集された血液、組織等の生体資料のことをいう。研究責任医師又は実施医療機関の管理者は、本研究計画書又は試料等の保管手順書に従って、試料を適切に保管する。

## 試料・情報の二次利用について

本研究で得られた試料及び情報を異なる研究目的で使用する可能性がある（二次利用）。その旨を同意・説明文書に記載し、研究対象者に説明したうえで同意を取得する。二次利用を行う場合には、必要に応じ新たに研究計画書を作成し、当該研究に意見を聴くべき倫理審査委員会で承認を得た後に実施する。

## 廃棄の手順及び方法

研究責任医師は、本研究において研究対象者から取得した試料及び情報、本研究の審議に関する記録及び資料を廃棄する場合は、特定の個人を識別することができないように必要な措置をとる。

実施医療機関の管理者は、研究責任医師から保存すべき試料、情報、記録及び資料等について、その保存の必要がなくなったことを通知されるまで保存する。

# 研究結果の帰属

本研究の結果として、特許権等の知的財産権が生じた場合、その権利は**********に帰属する。

# 実施体制

## 統括管理者

| 氏名 | ********** |
| --- | --- |
| 所属 | ********** |
| 役職 | ********** |
| 所在地 | ********** |
| 電話番号 | ********** |

## 研究責任医師

別紙2（実施医療機関及び研究責任医師一覧）参照

## 割付責任者

| 氏名 | ********** |
| --- | --- |
| 所属 | ********** |
| 役職 | ********** |
| 所在地 | ********** |
| 電話番号 | ********** |

## 統計解析担当責任者

| 氏名 | ********** |
| --- | --- |
| 所属 | ********** |
| 役職 | ********** |
| 所在地 | ********** |
| 電話番号 | ********** |

## データマネジメント担当責任者

| 氏名 | ********** |
| --- | --- |
| 所属 | ********** |
| 役職 | ********** |
| 所在地 | ********** |
| 電話番号 | ********** |

## モニタリング担当責任者

| 氏名 | ********** |
| --- | --- |
| 所属 | ********** |
| 役職 | ********** |
| 所在地 | ********** |
| 電話番号 | ********** |

## 研究事務局（責任者）

| 氏名 | ********** |
| --- | --- |
| 所属 | ********** |
| 役職 | ********** |
| 所在地 | ********** |
| 電話番号 | ********** |

# 引用文献

1. **国立研究開発法人国立がん研究センター.** がん情報サービス. (オンライン) (引用日: 2024年12月11日.) https://hbcr-survival.ganjoho.jp/graph?year=2014-2015&elapsed=5&type=c11#h-title.

2. **一般財団法人 日本消化器病学会.** 消化器難治癌シリーズ　膵癌.

3. **日本膵臓学会　膵癌診療ガイドライン改訂委員会.** 膵癌診療ガイドライン（2022年版）. 金原出版株式会社.

4. **Unno M, Motoi F, Matsuyama Y, et al.** Randomized phase II/III trial of neoadjuvant chemotherapy with gemcitabine and S-1 versus upfront surgery for resectable pancreatic cancer (Prep-02/JSAP-05). J Clin Oncol 2019;37 (4_suppl):189.

5. **Eguchi H, Takeda Y, Takahashi H, et al.** A prospective, open-label, multicenter phase 2 trial of neoadjuvant therapy using full-dose gemcitabine and S-1 concurrent with radiation for resectable pancreatic ductal adenocarcinoma. Ann Surg Oncol. 2019;26:4498-4505.

6. **Yamada D, Kobayashi S, Takahashi H, et al.** Results of a randomized clinical study of gemcitabine plus nab-paclitaxel versus gemcitabine plus S-1 as neoadjuvant chemotherapy for resectable and borderline resectable pancreatic ductal adenocarcinoma (RCT, CSGO-HBP-015). Ann Surg Oncol. 2024;31:4621-33.

7. **Ren Q, Kao V, Grem JL.** Cytotoxicity and DNA fragmentation associated with sequential gemcitabine and 5-fluoro-2'-deoxyuridine in HT-29 colon cancer cells. Clin Cancer Res. 1998;4:2811-8.

8. **Hidalgo M, Castellano D, Paz-Ares L, et al.** Phase I-II study of gemcitabine and fluorouracil as a continuous infusion in patients with pancreatic cancer. J Clin Oncol. 1999;17:585-92.

9. **Di Costanzo F, Carlini P, Doni L, et al.** Gemcitabine with or without continuous infusion 5-FU in advanced pancreatic cancer: a randomised phase II trial of the Italian oncology group for clinical research (GOIRC). Br J Cancer. 2005;93:185-9.

10. **Evans DB, Rich TA, Byrd DR, et al.** Preoperative chemoradiation and pancreaticoduodenectomy for adenocarcinoma of the pancreas. Arch Surg. 1992;127:1335-9.

**別紙1（利益相反事項）**

研究名称：切除可能膵癌に対する化学放射線療法と化学療法のランダム化比較試験

統括管理者：**********

本研究に関与する製薬企業等についてのCOI（研究に対する関与）：該当なし

本研究に関与する対象薬剤製薬企業等と、統括管理者・研究責任医師・研究分担医師等との開示すべきCOI：該当なし

**別紙2（実施医療機関及び研究責任医師一覧）**

|  | 実施医療機関 | | 研究責任医師 | |
| --- | --- | --- | --- | --- |
|  | 医療機関名・所属 | 所在地・電話番号 | 氏名 | 職名 |
| 1 | ********** | ********** | ********** | ********** |
| 2 | ********** | ********** | ********** | ********** |
| 3 | ********** | ********** | ********** | ********** |
| 4 | ********** | ********** | ********** | ********** |
| 5 | ********** | ********** | ********** | ********** |
| 6 | ********** | ********** | ********** | ********** |
| 7 | ********** | ********** | ********** | ********** |
| 8 | ********** | ********** | ********** | ********** |
| 9 | ********** | ********** | ********** | ********** |
| 10 | ********** | ********** | ********** | ********** |
| 11 | ********** | ********** | ********** | ********** |
| 12 | ********** | ********** | ********** | ********** |
| 13 | ********** | ********** | ********** | ********** |
| 14 | ********** | ********** | ********** | ********** |
| 15 | ********** | ********** | ********** | ********** |

第1版（2025年6月30日作成）
